# The cell behavior ontology: describing the intrinsic biological behaviors of real and model cells seen as active agents

**DOI:** 10.1093/bioinformatics/btu210

**Published:** 2014-04-22

**Authors:** James P. Sluka, Abbas Shirinifard, Maciej Swat, Alin Cosmanescu, Randy W. Heiland, James A. Glazier

**Affiliations:** Department of Physics, Biocomplexity Institute, Indiana University, Bloomington, IN 47405, USA

## Abstract

**Motivation:** Currently, there are no ontologies capable of describing both the spatial organization of groups of cells and the behaviors of those cells. The lack of a formalized method for describing the spatiality and intrinsic biological behaviors of cells makes it difficult to adequately describe cells, tissues and organs as spatial objects in living tissues, *in vitro* assays and in computational models of tissues.

**Results:** We have developed an OWL-2 ontology to describe the intrinsic physical and biological characteristics of cells and tissues. The Cell Behavior Ontology (CBO) provides a basis for describing the spatial and observable behaviors of cells and extracellular components suitable for describing *in vivo*, *in vitro* and in silico multicell systems. Using the CBO, a modeler can create a meta-model of a simulation of a biological model and link that meta-model to experiment or simulation results. Annotation of a multicell model and its computational representation, using the CBO, makes the statement of the underlying biology explicit. The formal representation of such biological abstraction facilitates the validation, falsification, discovery, sharing and reuse of both models and experimental data.

**Availability and implementation:** The CBO, developed using Protégé 4, is available at http://cbo.biocomplexity.indiana.edu/cbo/ and at BioPortal (http://bioportal.bioontology.org/ontologies/CBO).

**Contact:** jsluka@indiana.edu or Glazier@indiana.edu

**Supplementary information:** Supplementary data are available at *Bioinformatics* online.

## 1 INTRODUCTION

**Fig. 1.**
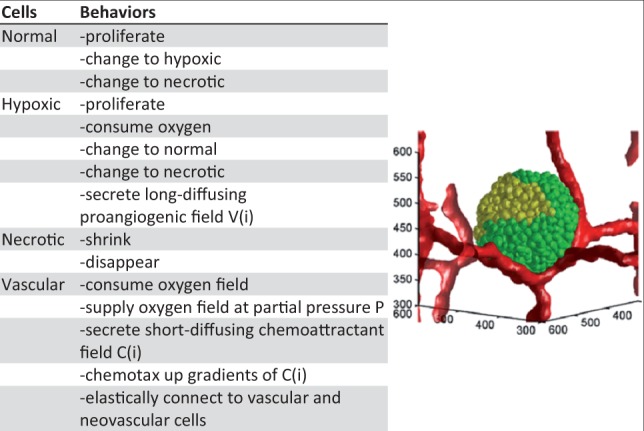
Left: section of a table from a typical publication of a multicell biological model (a model of tumor growth with angiogenesis) detailing some of the cell types and their processes and behaviors within the simulation (Shirinifard *et al.*, 2009). Right: simulation screenshot showing tumor cells (‘Normal’ = green and ‘Hypoxic’ = yellow) and vasculature (red). The microtumor contains thousands of cells. Axes are labeled in micrometer

Although all biological research requires the use of abstract models, currently no standard method exists for describing the biological content of microscope images of tissues, *in vitro* experiments or in silico simulations of the tissue dynamics of multicellular systems. In the in silico modeling domain, each modeling tool, e.g. CompuCell3D (Swat *et al.*, 2012), CHASTE (Dunn *et al.*, 2012) and OpenAlea (Pradal *et al.*, 2008), uses its own model-description language. (We include a glossary of terms, with hyperlinks, in the Supplementary Material.) These model-description languages focus on representing the computational implementations (simulations) of mathematical multicell models and depend on the specific computational methodologies the modeling tools use to implement objects and their dynamic processes. As a result, these languages do not adequately document the biology that the computational model seeks to describe, i.e. you cannot recreate the biological model from the simulation code. Publications of computational models usually include a prose description of the biological model in the body of the paper, with tables listing key biological components and processes. Figure 1 shows such a table from a publication describing a computational model of vascular growth during tumorigenesis (Shirinifard *et al.*, 2009). This *ad hoc* approach to biological model description and distribution has several drawbacks. First, the entity names (cells, molecules) and process names (oxygen consumption, cell proliferation) are not standardized, which impedes retrieval of the model in a search, and perhaps, the understanding and correct reuse of model components once found (not portable, consistent, retrievable or reusable). For example, the use of the word ‘proliferate’ to describe cell growth and division in Figure 1 means that searches using ‘mitosis’ as a query would not locate the model or the publication (not retrievable). In addition, some journals reproduce tables in papers as images, preventing a search engine from indexing the words within the table. Second, the human-readable description of the model in the publication resides separately from its computational instantiation rather than within the executable code that the authors ran to generate their results (not retrievable). Frequently, labels in the executable code such as cell-type names are inconsistent with those in the text of the publication (not consistent). Physical separation and inconsistencies both obscure the relationship between the underlying biological model that the authors intended to represent and the computational methods they used to instantiate the model.

This article presents ontological techniques to reduce these difficulties in the description of multicellular experiments and biological models. These tools allow developers of biological models to directly embed, or link to, a semantic description of the biologic model based on a new multicell-specific ontology. When used in conjunction with existing reference (naming authority) ontologies, such as the Gene Ontology (GO), ChEBI, Foundational Model of Anatomy (FMA), developers can create fully specified biological models, which will greatly enhance others’ ability to locate, understand, validate and redeploy these biological models.

### 1.1 Bio-ontologies

An *ontology* is a formalized description of a particular view of reality. They include a precisely defined set of *objects* (*classes*), a hierarchical *taxonomy* that organizes the individual objects into subsets, a set of *relationships* that may apply between objects and the minimal *syntax* needed to express the ontology's structure. In the past decade, a large number of biologically focused ontologies, *bio-ontologies*, have attempted to precisely define previously tacitly defined aspects of biology. Many bio-ontologies are available under the Open Bio-Ontologies (*OBO*) framework (Smith *et al.*, 2007) as well as via BioPortal (Whetzel *et al.*, 2011) maintained by the National Center for Biomedical Ontologies (Musen *et al.*, 2011). We can classify existing bio-ontologies as either *reference* or *application* ontologies. Reference ontologies primarily provide controlled vocabularies with sets of hierarchical relations. The *GO* (Ashburner *et al.*, 2000) is the most successful and widely used reference bio-ontology. GO provides three top-level term classes: *biological processes*, *cellular components* and *molecular functions*. GO serves as a ‘naming authority’ resource that classifies biological concepts into these three categories. GO has greatly facilitated the large-scale organization and cataloging of biological data at the cellular, subcellular and genetic levels (Huang *et al.*, 2009).

Application ontologies often describe the details of processes, typically using fewer classes and more complex set of relations, than reference ontologies. For example, the Systems Biology Ontology (*SBO*) (Courtot *et al.*, 2011) provides a controlled set of classes to describe the structure and dynamics of networks of biochemical reactions, or any process described by a set of coupled equations. SBO and the corresponding markup language specification, Systems Biology Markup Language (*SBML*), allow reaction models to be specified completely and are *machine implementable*, i.e. serve as executable programs (*code*). Adequately annotated, via links to suitable reference ontologies, an SBO/SBML model is also machine searchable; particularly when the model resides in an online repository such as the BioModels Database. The Ontology of Physics for Biology (*OPB*; Cook *et al.*, 2011) is another application bio-ontology that provides rigorous definitions of physical quantities (pressure, force, energy, etc.) and the mathematics that govern their relationships.

## 2 THE GOALS AND SCOPE OF CELL BEHAVIOR ONTOLOGY

We designed the Cell Behavior Ontology (CBO) to facilitate (i) the consistent and explicit definition of the biology relevant to multicell experiments (biological models), (ii) link this biology to a quantitative computational representation, (iii) automated searching of such models and (iv) analysis of such models for submodels. CBO annotation makes explicit the biology that a model represents to facilitate validation, falsification, searching, sharing and reuse of all model levels. Such sharing and reuse are key features of the US Department of Health and Human Services' Responsible Conduct of Research statement (Steneck *et al.*, 2007), which funding agencies around the world endorse.

The CBO focuses on cell and tissue biology, in particular, on the cell and non-cellular tissue components as semiautonomous agents with the characteristics of physical objects. For example, at a particular instant in time, a cell occupies space and has volume and a location. A cell interacts with other cells and non-cellular components of its local environment. The behavior of a cell over time (dynamics) is a function of both the state of its ‘internal machinery’ (e.g. the cell's type, cytoskeletal structure, DNA packing and internal metabolic processes) and the local environment. Because cells and tissues exist and change in time and space, the CBO must be able to describe the duration and extent of both the processes and spatiotemporal aspects of individual cells. The behaviors of larger aggregates of cells, for example tissues or organs, *emerge* from the interacting behaviors of the constituent cells, non-cellular components, such as the extracellular matrix, and external boundary conditions such as nutrient sources.

The CBO encompasses length scales that range from subcellular to cell aggregates (micrometers to millimeters) and time scales that range from seconds to decades. The CBO is phylum neutral; it should encompass viruses, prokaryotes, eukaryotes, plant and animal cells, as well as specialized cells such as erythrocytes.

The initial release of the CBO assumes spatial objects are 3D and exist in a standard Cartesian coordinate system. Two-dimensional multicell models present challenges to the development of a common ontological description because consistence with the physical world is often not possible. For example, the 3D concept of the volume unit as a length cubed corresponds to the area unit of length squared in a 2D model. This change of units affects many common parameters, such as concentrations, whose units change from mass/volume to mass/area in 2D models. To avoid this clash of units in different model dimensionalities, the CBO assumes that a model in one- or two-dimensions has an implied length of one along the unspecified axis(es).

As an application and process-oriented ontology, CBO resembles ontologies such as OPB and SBO more than reference ontologies such as the *FMA* (Rosse and Mejino, 2003) or GO.

## 3 DEVELOPMENT OF THE CBO

We developed the basic classes and structure of the CBO during six workshops held between 2009–2012 in (i) Bloomington, Indiana, USA, (ii) Bethesda, Maryland, USA, (iii) Heidelberg, Germany and (iv) Edinburgh, Scotland. These workshops included dozens of researchers in fields ranging from molecular biology to computational biology to ontology development and identified the core concepts the ontology needed to cover.

**Choice of Ontology Language:** Defining the CBO required us to choose an underlying ontology language. Many bio-ontologies use the *OBO* language (Smith *et al.*, 2007), so creating the CBO in OBO style would have provided interoperability with existing OBO resources. However, we decided to use Web Ontology Language (OWL) version 2 as the underlying ontology language for the CBO because it provides greater support for semantic reasoning (Lister *et al.*, 2010), searching and more flexible distribution options. We constructed the CBO using Protégé 4.

**Fig. 2.**
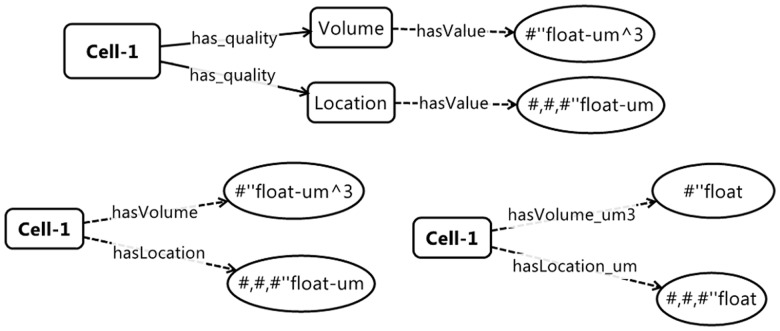
Examples of class-rich relation-poor (upper) versus class-poor relation-rich (lower) representation of some basic qualities of an individual cell (‘Cell-1’). Rounded rectangles represent a class or individual; ovals represent data values. OWL recognizes three fundamental property (relationship) types—*object properties* that link classes and/or individuals to each other, *data properties* that link classes or individuals with data values and *annotation properties* that link classes or individuals with annotations. Here, relations indicated with solid lines are OWL object properties, and dashed line relations are OWL data properties. The CBO uses the class-rich (upper) paradigm

**Class-rich versus Property-rich Formalism:** Developing an ontology requires partitioning concepts between classes and properties. The optimal partitioning approach largely depends on the anticipated uses of the ontology, as the same conceptual structure can be represented with many classes and few properties or fewer classes and more relationships (see Fig. 2). SBO contains >500 classes but only one explicit property, *isA* (much of SBO's property information is specified in SBML). GO includes ∼35 000 classes and five properties. Because CBO is an application- and process-oriented ontology, which must be able to specify spatiotemporal objects, and because classes are easier to embody and extend computationally than are properties, we decided on a class-rich properties-limited structure. The CBO's properties list is based on the OBO Relationship Ontology (*RO*; Hoehndorf *et al.*, 2010) to which we have added a few additional CBO-specific properties (relationships). The full list of properties is available in the CBO OWL file and the Supplementary Material.

### 3.1 The CBO core

To ensure that the CBO is interoperable with existing ontologies, we reuse (or link to) existing ontologies whenever they provide appropriate classes or concepts.

**Underlying Foundational Ontology:** As a basic underlying ontology, CBO uses the Basic Formal Ontology (*BFO*; Grenon and Smith, 2004). BFO provides a foundational set of classes suitable for describing biological systems. BFO consists of two high-level domains, *Snap* and *Span*. The Snap (*continuant*) domain represents physical *objects* and object *qualities* that persist over time. The Span (*occurrent*) domain represents time as a series of linked *instances* and represents processes that occur over time.

Because classes and relationships in the CBO must map to instances of computable code, the initial CBO has a fairly limited number of both classes and relationships. Many bio-ontologies have too many classes to be practical for defining computational instantiations of biological processes; for example, GO currently contains nearly 35 000 classes. Where possible, we have mapped CBO classes to high-level GO biological process classes such as ‘cell–cell adhesion’ and not to lower-level (finer grained) GO classes such as ‘homotypic cell–cell adhesion’. Many cell phenotypic qualities, such as volume, map to classes in the Phenotypic Quality Ontology (*PATO*; Gkoutos *et al.*, 2004). Because PATO contains 2300 classes, CBO includes only those classes that describe cells as spatial objects. The CBO also uses the Unit Ontology (*UO*).

In a future release, the CBO will support the *OPB* (Cook *et al.*, 2011), which supports the mathematical description of biological forces and energetics, and the tools to describe those entities in computational models.

In the following discussions, we will indicate CBO classes in **bold** face, meta-model classes in **underlined bold** face, relations in *italics*, and individuals in monospaced fonts.

**Relationships (Properties):** OWL recognizes three types of relationships (‘properties’ in OWL): *Object Properties* (e.g. subClassOf), *Data Properties*, which link an object to a particular value, and *Annotation Properties*, which link an object or property to an annotation. For its basic set of Object Properties, CBO uses the *OBO-RO* (Smith *et al.*, 2005). OBO-RO provides a set of common relationships used in many existing bio-ontologies. In addition, the CBO includes a has_Quality relationship, which is missing in some version of OBO-RO.

The CBO includes a small set of data properties that computational multicell models need. One type of data property common to computational models describes object qualities that have both a *current value* and a *target value*. For example, in many modeling paradigms, a particular cell at a particular instant has both an *actual volume* and a *target volume* and both relate to the cell's ‘volume’ class. Therefore, we include two OWL data properties, ‘*hasIntValueTarget*’ and ‘*hasFloatValueTarget*’, which are parallel to ‘*hasIntValue*’ and ‘*hasFloatValue*’ data properties. For example, an individual cell (an instance of class ‘cell’) may have relationships to two instances of class ‘volume’—one via a ‘hasFloatValue’ data property and the other via a ‘hasFloatValueTarget’ data property. The complete list of relations in the CBO is given in the Supplementary Material.

### 3.2 High-level objects

**Fig. 3.**
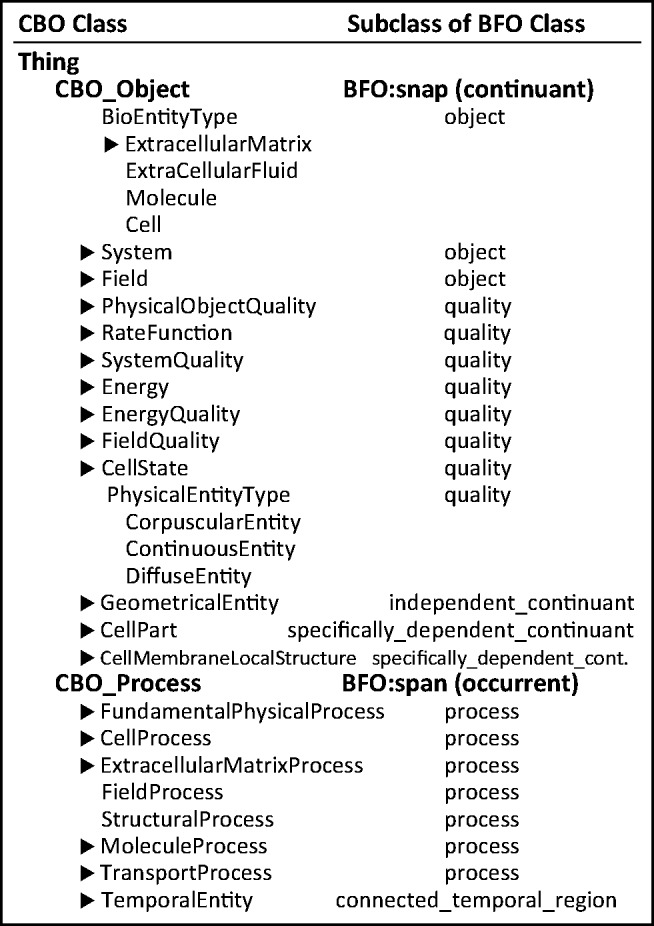
High-level classes in the CBO (left column) and corresponding BFO classes (right column). **CBO_Object** (BFO:snap) describes the physical entities and entity qualities of a biological model. **CBO_Process** describes the processes in which **CBO_Object**(s) participate. Classes marked with ▸ have additional subclasses that are not shown

The initial version of the CBO has just over 240 classes. Figure 3 shows the top-level classes in the CBO. The two main classes **CBO_Object** and **CBO_Process** correspond to **BFO:snap** (continuant) and **BFO:span** (occurrent), respectively.

### 3.3 Physical objects

**CBO_Object** (BFO-independent and BFO-dependent continuants) includes physical objects such as cells and object qualities such as volume and location. The **CBO_Object** class describes biological entities and their computational representations. The **CBO_Object:PhysicalEntityType** class describes how a particular object occupies space. The **PhysicalEntityType:CorpuscularEntity** describes spatially exclusive entities with explicit boundaries. Often, a **CBO_Object:Cell** (a biological descriptor) would also be of type **PhysicalEntityType:CorpuscularEntity.** The **CopuscularEntity** class states that the cell has a defined boundary and is spatially exclusive (no other spatially exclusive object can occupy the same space at the same time). This class describes how the cell is modeled in a particular meta-model. The **PhysicalEntityType:ContinuousEntity** describes physical objects that lack bona fide (intrinsic or visible) boundaries and are often treated as portions, e.g. a portion of the extracellular medium or a portion of blood. The **PhysicalEntityType:DiffuseEntity** describes non-spatially exclusive physical objects that can ‘overlay’ objects of the other two types. A **DiffuseEntity** description is used to describe how fields (chemical, magnetic, electrical, etc.) superimpose on spatially exclusive objects such as cells.

**Fig. 4.**
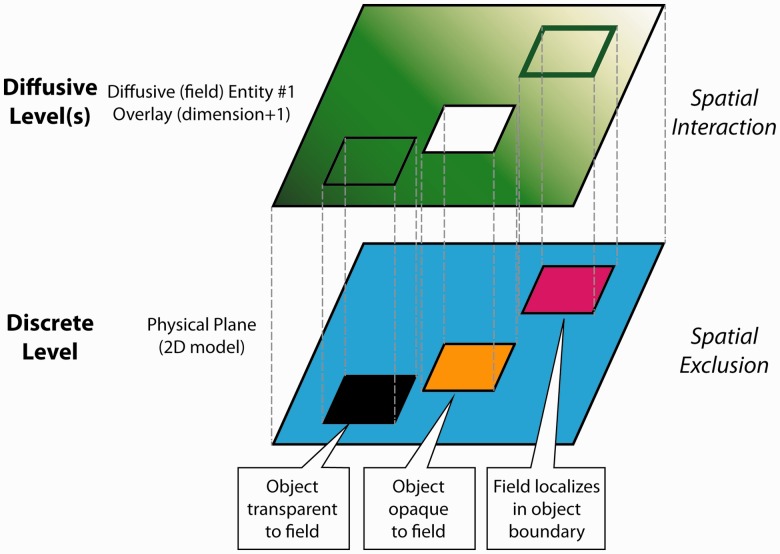
The lower panel shows three CBO discrete physical objects (**CBO:CorpuscularEntity**) embedded in a blue field of medium (**CBO:ContinuousEntity** with the additional quality of a **CBO:PhysicalObjectQuality:Fill**). These objects are spatially exclusive. No two objects of these types can occupy the same region of space at the same time. Here we represent the physical space in 2D as a plane containing three rectangular objects embedded in the blue fill. A **CBO: Field** is not spatially exclusive and by default can overlay any other object (upper 2D plane). A **CBO:Field** can interact with the discrete level. For example, specific discrete objects may be opaque to a given field

Fields are a special class of physical objects and are the primary way that the CBO describes distributions of molecules (see Fig. 4). Because the sizes of most molecules are well below the spatial scale covered by the CBO, terms for describing individual molecules are not included. Instead, the CBO treats them as fields containing position-dependent concentrations (or other ‘*field strength*’ values) and processes that describe how the fields evolve (diffuse, advect, react, decay) over time. In addition, **CBO_Process:FundamentalPhysicalProcess:BarrierCrossing** describes how bona fide boundaries, such as cell membranes, affect field diffusion.

In a typical CBO model, each cell is an OWL instance (individual) of the class **Cell**. The instance has an associated list of model-specific qualities (volume, location, etc. if the cell is modeled as a **CorpuscularEntity**) that it inherits from the prototype class for that cell type. That is, each quality in an individual cell's prototype class is understood to be instantiated for every instance of that cell type. The actual values of the cell's qualities are defined for each individual time instance for which the cell exists.

### 3.4 Processes

The **CBO_Process** branch of the CBO describes existential processes of cells such as cell–cell adhesion, cell division and cell death as well as existential processes of other model components such as fields or portions of extracellular matrix. **CellProcess**, **ExtracellularMatrixProcess** and **MoleculeProcess** describe common biological processes for the corresponding physical object classes. The **FundamentalPhysicalProcess** class includes existential and dynamic processes such as object creation, destruction and movement. Processes typically involve participation of objects from the **CBO_Object** branch such as cells or fields. Processes are applied to individuals based on class membership and the properties of the process. A process is defined for a model-specific cell *prototype* class (or classes), which is typically a subclass of **CBO_Object: BioEntityType**. All instances (individuals) of a prototype class inherit the processes and process qualities of the prototype class.

Similarly, **CBO_Object:ContinuousEntity** (portions) and **CBO_Object:DiffuseEntity** (fields) may also have process lists (such as their diffusion process), and the process lists apply to all individual portions of a **ContinuousEntity** or to all points in the field for a **DiffuseEntity**. This inheritance structure is consistent with the approaches of most computational multicell models.

In addition, certain process are understood as conditionally occurring, i.e. they occur between individuals of the prototype classes subject to meeting a specified set of restrictions determined dynamically at a given instant. For example, the **CBO_Process:CellCellAdhesion** process is understood to occur between two cell instances, at a particular time, if and only if those two cell instances are actually in *contact* at that time.

### 3.5 Description of spatiality in multicell models

Existing bio-ontologies do not describe spatiality effectively for the purpose of multicell models. Neither FieldML (Christie *et al.*, 2009) nor a proposed spatiality extension to SBO/SBML (http://sbml.org/Community/Wiki/SBML_Level_3_Proposals/Spatial_Geometries_and_Spatial_Processes) provide methods to describe adequately the spatiality of multicell experiments and models. Describing the geometry of individual cells and the position-dependent magnitude of fields using OWL classes for voxel (or pixel) or cell-center lists would be extremely verbose and would replicate that verbosity for every time step (snapshot) during an experiment or simulation. Unlike many bio-ontologies, the CBO needs to describe a large number of ‘individuals’, where an individual, for example a particular cell, has a set of qualities with unique values. In knowledge domains with large numbers of individuals, particularly when the characteristics and number of individuals is highly dynamic, it is common to define the individuals externally to the ontology. In many cases, a relational database is used to store the list of individuals and their time-dependent qualities. In the CBO, individuals and spatial definitions are, in general, external to the ontological description of the model. As an experiment is run, the list of individuals (such as cells), along with the spatiality of the individuals, is not added into the OWL CBO ontology but instead described in a series of external files. The OWL CBO file describes the components of the model, such as cell types and fields, along with the initial state of the system. The state of the model at any later time is described using a combination of the OWL CBO meta-model and the external file(s).

We describe the spatiality of CBO models using the Visualization Tool Kit (*VTK*) *legacy 3D model-description file formats*. For grid-based segmented microscopic images or computational models of cells like the GGH approach (Swat *et al.*, 2009), which represents biological cells and extracellular components as collections of voxels, the VTK file format for a lattice (STRUCTURED_POINTS) lists the voxels belonging to individual cells (or model components that are treated like generalized cells such as portions of serum) and the voxel-based values of fields. For center models, which represent cells as spheres with center positions and an interaction radii, the VTK file format POLYDATA describes the coordinates of the centers and radii of spatial objects. Fields can be represented in both cases using a discretized VTK matrix for each field. The Supplementary Material provides examples of these formats.

VTK file formats have a number of important advantages. Existing open-source code libraries can read, write, visualize and animate VTK datasets. In addition, existing multicell modeling tools, such as Compucell3D, OpenAlea and CHASTE, already support input and output of VTK files. The CBO allows linking of a CBO-defined meta-model to a particular VTK data file (or set of VTK files) by adding a link to the VTK file(s) to the CBO model. A **CBO: TemporalInterval** value provides the *multiplier* of the dataset's time coordinate (typically encoded in the VTK file name). A *hasVTKTypeID* (an OWL data property) for each cell-prototype class in the model specifies the identifier that the VTK file uses for members of that object class. For additional details, see the Supplementary Material.

## 4 CBO USE CASES

As a use case, we present a CBO description of part of a published model of tumor growth with angiogenesis (Shirinifard *et al.*, 2009; see Fig. 1). This 3D model is defined on a 180 × 180 × 180 voxel lattice that includes 5.8 million lattice points for every ‘snapshot’ in the simulation. The model includes the cell types ‘tumor’ (in the publication called ‘normal’) with two subphenotypes ‘hypoxic’ and ‘necrotic’ and vascular endothelial cells with two subphenotypes of ‘inactive’ and ‘active’. The 3D model includes several thousand cells along with two diffusible fields, the autonomous cellular processes of growth, division and necrosis, cell-type-specific processes for cell–cell adhesion, secretion and consumption of two diffusing molecular signals, a diffusing nutrient field and a cell-field process of chemotaxis. The model evolves over time producing a series of ‘snapshots’ with the number of tumor and endothelial cells changing and the cells and fields organizing into a complex spatial structure as an emergent property of the model.

Here we use the CBO to create a meta-model of the tumor growth with angiogenesis multicell model. *This meta-model is a new ontology that is based on the CBO and extended to include the objects and processes specific to this particular model.*

**Fig. 5.**
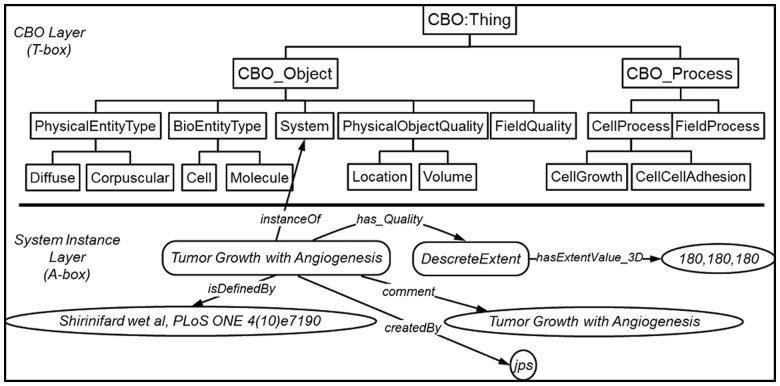
Use case definition of the model of Figure 1. Here we define the spatiotemporal domain including its extents, name and a literature reference. The upper portion of the figure shows the top level and a few lower levels of the CBO (T-Box features). The lower portion of the figure shows the beginning of the definition of the meta-model. Individual instances (A-Box features) and data values particular to this meta-model are defined. OWL Classes are denoted as rectangles, individuals as rectangles with rounded corners and data values as ovals. Unlabeled relations are isA. T-Box features describe relationships between classes, for example ‘System’ ‘isA’ ‘CBO_Object’. A-Box features describe Instances of Classes. For example, a particular cell is an ‘instanceOf’ the class ‘Cell’. T Box statements are the core components of a particular ontology and are permanent parts of that ontology. A-Box statements are more dynamic and typically describe aspects of a particular model instantiated using instances of the classes defined in the T-Box

**Define the spatiotemporal domain (****Fig. 5****):** The first step in describing the multicell computational model using the CBO is to describe the basic ‘universe’ that the simulation represents. This description includes both a description of the overall biology being represented (e.g. GO:angiogenesis) and characteristics of the model domain such as the model spatial and temporal extents, the behavior at the edges of the domain, for example a ‘wall’ versus a periodic boundary, and the modeling platform (e.g. **ComputationalPlatform:Compucell3D**). In Figure 5, the spatiotemporal domain is defined as a regular grid (**CBO:DiscreteExtent**) of 180^3^ voxels. Not shown in Figure 5 is the definition of the lattice length unit, which is defined by an individual of the class **CBO:SystemPixelDistanceScale** that *hasFloatValue* ‘4.44’ and *hasUnit* ‘**UO:micrometer**’ (each voxel in the model has an edge length of 4.44 µm).

**Fig. 6.**
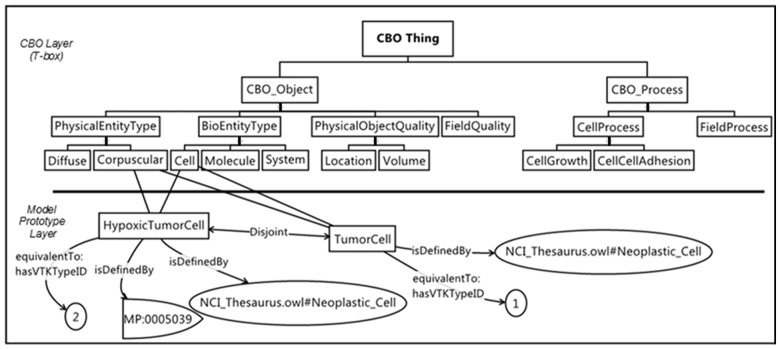
The prototype cell classes, specific to this model, **HypoxicTumorCell** and **TumorCell** are subclasses of both **CBO:Corpuscular** and CBO:Cell classes. We also show links to reference ontologies. MP:0005039 is the Mammalian Phenotype ‘hypoxia’. The equivalentTo:hasVTKTypeID relation links a cell prototype to a unique cell-type identifier for use in VTK files. For example, any cell in a VTK file with TypeID=‘1’ is an instance of the class **TumorCell**. As in Figure 5, the upper part of the figure shows a portion of the CBO, whereas the model-prototype layer below is part of a new ontology describing a particular model

**Define prototype cell classes (****Fig. 6****):** We next define prototype cell classes. We annotate these using appropriate reference ontologies such as the Cell Ontology (Meehan *et al.*, 2011). The prototype cell classes constitute a second ontological layer below the CBO layer, as they involve the creation of new OWL classes that extend the CBO's T-Box. In this example, we create the new meta-model-specific classes **TumorCell** and **HypoxicTumorCell**. These two classes are subclasses of both **CBO_Object:BioEntityType:Cell** (their biological object type) and **CBO_Object:PhysicalEntityType:Corpuscular** (because the model represents them as corpuscular entities). In the CBO-defined meta-model, the new classes for the prototype cells have inheritance from higher-level CBO objects. In particular, they participate in relationships that define both the biological concept (*cell*) and the representation in the model (*corpuscular entity*). Additional relationships (inheritances) such as spatiotemporal domain and 3D representation are implied. In this example, the *system* is described as a regular grid (**DiscreteExtent** in Fig. 5).

**Fig. 7.**
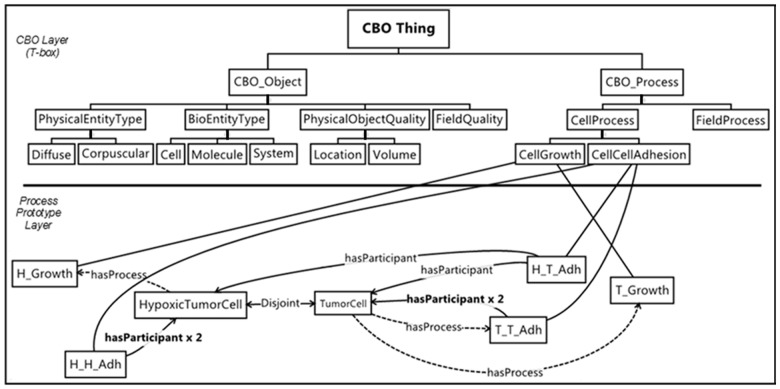
Definition of processes for cell growth and cell–cell adhesion relative to prototype cell classes. For example, the prototype HypoxicTumorCell class has a ***hasProcess*** relationship to the H_Growth class, which is a subclass of CBO_Process:CellGrowth

**Define processes for prototype classes (****Fig. 7****):** The processes instantiated in the model involve prototype classes and are subclasses of the relevant **CBO_Process** classes. We annotate process classes using appropriate reference ontologies (e.g. GO:CellCellAdhesion). For example, the prototype cell class **TumorCell** has a *participates_in* relationship to an instance named T_Growth (an instance of **CBO_Process: … :CellGrowth**). A process in which two individuals participate, such as cell–cell adhesion, requires prototype classes for each possible pairwise interaction, for example, H_T_Adh (an instance of **CBO_Process: … :CellCellAdhesion**) for adhesion processes between instances of **HypoxicTumorCell** and **TumorCell**. Alternative ways to describe the *participates_in*, and *process_of*, adhesion between cells include basing the adhesion on cellular qualities associated with the surfaces of the cells, such as the concentration of membrane-bound adhesion molecules.

Figure 7 shows selected processes; additional processes are defined in the complete model (see Supplementary Material). Figure 7 also defines a process for the death (**CBO:CellDeath**) of individuals of the class **HypoxicTumorCell** as well as a pair of processes that describe the interconversion (**CBO:PhenotypicChange)** between individuals of the **TumorCell** and **HypoxicTumorCell** classes**.**

**Fig. 8.**
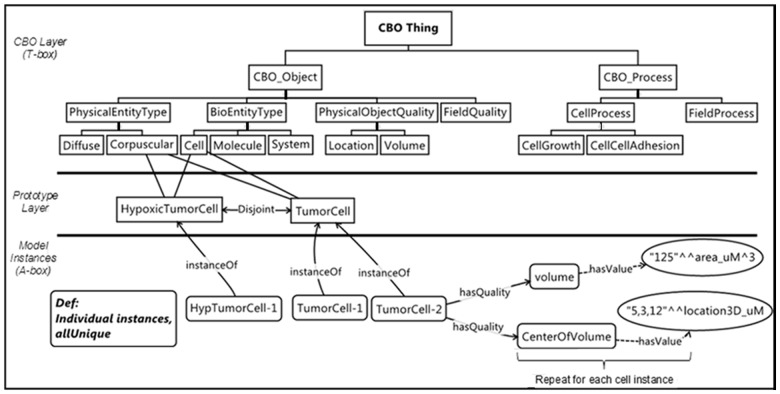
Individual cells are instances (**OWL:individual**) of prototype cell classes. Individual cells inherit the processes of the prototype cell classes. In practice, we declare cell instances and qualities such as location and volume (bottom portion of the figure) external to the OWL file, using data in the VTK file(s)

**Define individual cell instances (****Fig. 8****):** We next use the cell prototypes to define the individual cell instances present in an experiment or model. We could explicitly define these instances in the CBO meta-model file, including their spatial structure, to specify the initial state of the model; however, it is more straightforward to describe the initial and subsequent configurations via the VTK auxiliary files.

The completed OWL meta-model is a new ontology that imports the CBO and other reference ontologies (RO, UO) and specifications (RDF, OWL) into a meta-model encapsulating the biology in the model. The meta-model builds a semantic description of the biology being modeled using reusable components. For example, other tumor growth models could reuse the cell prototype **TumorCell**.

When an executable-code simulation of a CBO-defined meta-model runs, it creates a series of snapshots as the simulation evolves over time. The number of individuals, the classes (prototypes) of the individuals (e.g. cells) and the qualities (location, volume, etc.) change over time. The CBO-defined model contains links to a set of VTK files that contain simulation snapshots. A particular VTK file represents a particular time point from the simulation. The VTK file name and the VTK file's associated CBO meta-model file code the time represented.

The CBO meta-model file specifies VTK file characteristics. For example, for the model of tumor growth with angiogenesis, the meta-model file links to the VTK files as [in Manchester Syntax (Manchester Syntax is more human-friendly than is RDF/XML Syntax, see www.webont.org/owled/2008dc/papers/owled2008dc_paper_11.pdf)]:

 Individual: TumorGrowthWithAngiogenesis

  Types: System

  Facts:

   has_Quality use_case_1_extent,

   has_Quality systemPix2DistSc,

   has_Quality

     TumorGrowthWithAngiogenesis_VTK_InitialFile,

   has_Quality

     TumorGrowthWithAgniogenesis_VTK_FileTimeStep,

   has_Quality tumorGrowthWithAngiogenesis_VTK_                          FileRoot

 Individual: tumorGrowthWithAngiogenesis_VTK_                          FileRoot

  Types: CBO:VTK_FileRoot

  Facts: hasStringValue “use_case_1"

 Individual: TumorGrowthWithAngiogenesis_VTK_                       InitialFile

  Types: CBO:VTK_InitialCondition

  Facts: hasStringValue “use_case_1_0000000000.vtk"

 Individual:

  TumorGrowthWithAgniogenesis_VTK_FileTimeStep

   Types: CBO:VTK_FileTimeStep

   Facts: CBO:hasFloatValue “1",

           CBO:hasUnit “minute"

The above identifies the file root name (**VTK_FileRoot**) ‘use_case_1’, for the set of VTK files. The VTK file that contains the initial conditions (**VTK_InitialCondition**) for the simulation is ‘use_case_1_0000000000.vtk’. The scale factor to convert the numeric part of the VTK file name into a time coordinate (**VTK_FileTimeStep**) is ‘1’ with the units of ‘minute’. For the above example, a VTK file named ‘use_case_1_0000000600.vtk’ would contain the configuration of the objects and fields in the simulation at a simulated time of 600 min.

### 4.1 Representative uses of CBO annotation of models

The CBO has multiple uses. The use case above created a meta-model in OWL-2 describing the objects, processes and links to simulation snapshots (spatial specifications of the model at specific simulated times). The Supplementary Material includes sample files describing the tumor growth with angiogenesis model, including an OWL/XML format file containing the complete CBO with the model-specific classes and instances described above, the initial model configuration VTK file and VTK files describing the model at later instants in a typical simulation.

The CBO can also serve as a reference (naming authority) ontology, providing a controlled vocabulary to annotate experiments or models. In Supplementary Table S3 of the Supplementary Material, we have annotated the objects and processes of the angiogenesis model in Figure 1 using the CBO. This annotation regularizes the description of the components of the model. If we include these annotations *directly* in the simulation executable code (which in this case consists of Python and XML files) and if we store the code in an open Web-accessible location, then Web indexing engines like Google will index the files, making them searchable and increasing their shareability. Within executable code, language-specific comment characters, for example the # character in Python, hide the annotations from the program interpreter or compiler. In the Python code snippet below, we have added the CBO annotations as comments (highlighted in ***bold-italics***) directly in the code that define the growth and death processes for the tumor cells from Figure 1.

***# CBO CBO_Object SystemQuality ComputationalPlatform***

***# Compucell3D***

***# CBO CBO_Process CellProcess CellGrowth***

class VolumeParamSteppable(SteppablePy):

   …

***   ******# CBO CBO_Process CellProcess CellDeath Necrosis***

***   ******# GO:0008219 cell death***

   #Necrotic Cells

   if cell.type == 3:

     # cell growth rate equation

     cell.targetVolume-=0.5

     cell.lambdaSurface=0

Embedding annotations as comments works for any reference ontology: the example above also includes the GO term for cell death. We have partially annotated one of the Python scripts for the angiogenesis model and placed the file in a public directory on a Web server. As of this writing, a Google search for ‘ComputationalPlatform Compucell3D CBO_Process CellDeath’ (all CBO terms in the annotated Python script) locates the Python file as the first Google hit.

A third use of the CBO is as an annotation technique for microscopy images. A CBO meta-model for an image would identify the cell types (as well as other biological features such as basement membranes), which can then be linked to a VTK file containing the image data. The VTK file describes the pixels (or voxels) that belong to a particular cell (or other area or volume object) of a particular class defined in the meta-model.

## 5 CONCLUSION

This initial release of the CBO provides a framework for describing the objects, processes and object-to-processes links typical of multicell experiments, models and simulations. Auxiliary VTK files provide a convenient standard description of the spatiotemporal aspects of the biological model. In future work, we will extend the CBO to serve as the basis for a model-description language that can describe both the modeled biology and the *computational methods* a particular computational implementation uses to model that biology. This model-description language may be a markup language based on the CBO, similar to the SBO and SBML pair, or we may use the CBO in OWL-2 in conjunction with appropriate ontologies, such as the OPB, as both the ontological basis language and the executable markup language.

We hope that the CBO will lead to the creation of repositories of multicell spatiotemporal models similar to, or extending, the BioModels repository. The creation of such repositories of shareable models will facilitate the creation of new validated computational multicell models. In addition, annotating a model's source code using the CBO (and other bio-ontologies) and storing it as plain text files in a publicly accessible directory on the World Wide Web would allow the file to be located using standard Web search engines, reducing the need for a central model repository.

## 6 SUPPLEMENTARY MATERIAL

Supplementary Material is available online. This material includes the CBO OWL meta-model file for the use case in Figure 1. In addition, a step-by-step document provides detailed instructions on creating an OWL meta-model using the CBO in Protégé 4. This document also describes the VTK file formats for both lattice-based and center (lattice-free) models and includes a Python program for extracting information programmatically from both CBO-OWL meta-model files and the linked VTK files.

Supplementary Data
