# Supplementary material for: The cell behavior ontology: describing the intrinsic biological behaviors of real and model cells seen as active agents
Source: Bioinformatics. 2014 Apr 22;30(16):2367–74. doi: 10.1093/bioinformatics/btu210 (PMC4133580; doi:10.1093/bioinformatics/btu210)
Supplement: Supplementary Data [file supp_30_16_2367__index.html]

The Cell Behavior Ontology: Describing the intrinsic biological behaviors of real and model cells seen as active agents — The cell behavior ontology: describing the intrinsic biological behaviors of real and model cells seen as active agents — The cell behavior ontology: describing the intrinsic biological behaviors of real and model cells seen as active agents — Supplementary Data 

# The cell behavior ontology: describing the intrinsic biological behaviors of real and model cells seen as active agents

## Supplementary Data

files

**Files in this Data Supplement:**

- Supplementary Data - zip file
