# Supplementary material for: The cell behavior ontology: describing the intrinsic biological behaviors of real and model cells seen as active agents
Source: Bioinformatics. 2014 Apr 22;30(16):2367–74. doi: 10.1093/bioinformatics/btu210 (PMC4133580; doi:10.1093/bioinformatics/btu210)
Supplement: Supplementary Data [file supp_btu210_Supplemental_Material.zip › Supplemental Material/_Readme.docx]

_ReadMe.docx

12/23/2013

Supplemental Materials to " The Cell Behavior Ontology: Describing the intrinsic biological behaviors of real and model cells seen as active agents ",

James P. Sluka, Maciej Swat, Abbas Shirinifard, Alin Cosmanescu, Randy Heiland, James A. Glazier.

Biocomplexity Institute, Indiana University, Bloomington, Indiana USA

This folder contains:

- This **_Readme.docx** file.
- Supplement 1 (**Supplement_1.docx**) to the paper "The Cell Behavior Ontology: Describing the intrinsic biological behaviors of real and model cells seen as active agents", Sluka *et al*.
- The CBO meta-model file for the Shirinifard Vascular Tumor model (**use_case_1.owl**) described in the paper.
- A Compucell3D steppables file in Python annotated with CBO terms as an example of using CBO (and other ontological terms) to annotate a plain text file suitable for placing in a publically accessible location on the web for Google indexing. (**angio_growth_steppables_08052009_01_45_36__CBOannotated.py**).
- A folder with a step-by-step description of annotating a Compucell3D model of tumor growth with angiogenesis (CBO_Step_by_step). This folder includes:
  - The step by step instructions for creating a CBO-based metamodel description. This document also includes examples of using VTK files to store cell models in both lattice and center model forms (**CBO_Step_by_step.docx**).
  - A Python script (described in the CBO_Step_by_step) that extracts information from the CBO metamodel file and the Compucell3D output files (**Demo_data_extract_CBO_VTK.py**)
  - A folder with Compucell3D scripts, simulation output files (**.png** and **.vtk**), and other files associated with both the Compucell3D model and the CBO OWL metamodel.
