# Supplementary material for: The cell behavior ontology: describing the intrinsic biological behaviors of real and model cells seen as active agents
Source: Bioinformatics. 2014 Apr 22;30(16):2367–74. doi: 10.1093/bioinformatics/btu210 (PMC4133580; doi:10.1093/bioinformatics/btu210)
Supplement: Supplementary Data [file supp_btu210_Supplemental_Material.zip › Supplemental Material/CBO_Step_by_step/CBO_step_by_step.docx]

Step-by-Step instructions for creating a Multi-cell model
description using CBO in Protégé 4

James P. Sluka
Biocomplexity Institute
Indiana University

# Version

This is version 1.0 of this Step-by-Step document, created December 2013. The most recent version of this document is available at: <http://cbo.biocomplexity.indiana.edu/cbo/>.

Contents

[Version 1](#_Toc376185980)

[Overview 2](#_Toc376185981)

[Outline 2](#_Toc376185982)

[Multicell model of tumor growth with angiogenesis 2](#_Toc376185983)

[Describing a multi-cell model using the CBO in OWL using Protégé 4 3](#_Toc376185984)

[Define the System 3](#_Toc376185985)

[Define the Cell Prototypes 6](#_Toc376185986)

[Define the Processes 9](#_Toc376185987)

[Cell Growth 9](#_Toc376185988)

[Cell Death 12](#_Toc376185989)

[Cell Division (Mitosis) 13](#_Toc376185990)

[Cell-Cell Interactions (Adhesions) 15](#_Toc376185991)

[Field Creation 19](#_Toc376185992)

[Chemotaxis 24](#_Toc376185993)

[Conclusion 26](#_Toc376185994)

[Sample Usage of VTK files for Lattice and Center Based Models 27](#_Toc376185995)

[Example 1: Lattice model with cell types and a field. 27](#_Toc376185996)

[Example 2: Center model with cell types, cell centers and radii. 29](#_Toc376185997)

[Example 3: Center model with cell types and fields. 30](#_Toc376185998)

[Sample SPARQL Queries 32](#_Toc376185999)

[Sample Python programs for extracting information 34](#_Toc376186000)

[Configuring Protégé 4 35](#_Toc376186001)

[Annotation Template 35](#_Toc376186002)

[Render with Label vs. Name 36](#_Toc376186003)

[Reasoners 36](#_Toc376186004)

# Overview

This document provides step-by-step instructions for describing a multicell model using the Cell Behavior Ontology (CBO) as implemented in Protégé 4. The CBO is described in "The Cell Behavior Ontology: Describing the biology and models of intrinsic behaviors of cells as active agents," JP Sluka, A Shirinifard, M Swat, A Cosmanescu, R Heiland and JA Glazier.

# Outline

1. Multicell model of tumor growth with angiogenesis.
2. Describing a multi-cell model using the CBO in OWL implemented in Protégé 4^[[1]](#footnote-1)^.
3. Sample Python programs for extracting information from a CBO-OWL file and associated VTK output files produced by a simulation.
4. Sample SPARQL queries.
5. Notes on configuring Protégé 4.

# Multicell model of tumor growth with angiogenesis

We use a multi-cell model of tumor growth with angiogenesis (blood vessel formation) as our sample multicell simulation. This simulation is performed using the open-source multi-cell simulation software package Compucell3D^[[2]](#footnote-2)^ (*CC3D*) and a similar model has been published.^[[3]](#footnote-3)^ This particular simulation is based on the demonstration CC3D script that is part of the CC3D distribution. The model includes tumor cell types "Proliferating" and "Necrotic" and vascular pseudo-cell types "Vascular" and "NeoVascular". The model also includes three diffusing fields: "Glucose", a nutrient field generated by the two vascular cell types and consumed by the two tumor cell types, an endothelial-derived short diffusing VEGF isoform (*VEGF1*) and a long diffusing tumor-derived VEGF isoform (*VEGF2*) field. The model also includes growth and division of the NeoVascular and Tumor cell types and conversion from the Tumor cell type to the Necrotic cell type when a cell's local nutrient concentration drops below a threshold. Figure 1 shows screen shots of three time points from the simulation.


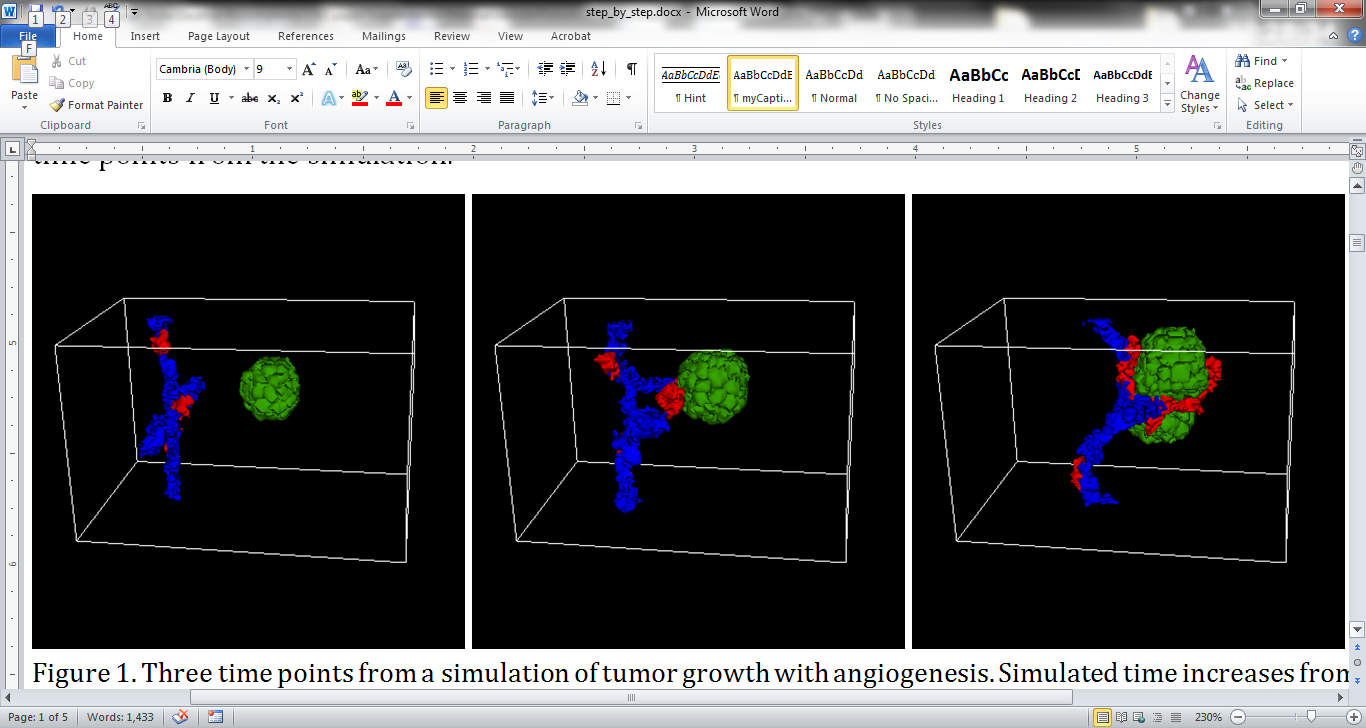

**Figure 1.** Three time points from a simulation of tumor growth with angiogenesis. Simulated time increases from left to right across the three images. In the first image a small multicell tumor (green) is adjacent to a vascular network (red and blue). In the second image the tumor has grown and the vascular network has begun to expand and to migrate towards the tumor. In the third image the tumor has grown further and new vasculature (red) is forming, which keeps the growing tumor supplied with nutrients.

This CC3D simulation is defined using four files:

1. VascularTumor.xml describes the basic cell types, model dimensions and simulation parameters
2. VascularTumor.py links various components of the simulation to the CC3D simulation environment
3. VascularTumorSteppables.py contains the functions describing cell growth and mitosis
4. Glucose_300.dat contains the starting conditions for the Glucose field.

These files are part of the standard CC3D distribution and can be found in the directory \CompuCell3D\Demos\BookChapterDemos_ComputationalMethodsInCellBiology\VascularTumor\Simulation.

The output of the CC3D simulation consists of a series of 2D projections of the model (as shown in Fig. 1) along with VTK files that give the full three dimensional model, including the diffusible fields, at fixed time intervals during the simulation.

# Describing a multi-cell model using the CBO in OWL using Protégé 4

To start, download copies of the CBO and Unit Ontology to your local file system from:

http://cbo.biocomplexity.indiana.edu/svn/cbo/trunk/CBO_1_0.owl
http://cbo.biocomplexity.indiana.edu/svn/cbo/trunk/Import_Ontologies/uo.owl

To describe this model using the CBO-OWL in Protégé 4 we start with a new (empty) ontology and name it "AngioTumor." This ontology is saved in the default format of "RDF/XML". We then import the CBO and the Unit Ontology files downloaded above using;

"Active Ontology" <tab> : "Ontology imports" view "Direct imports" click on the circle "+" sign, select "Import an ontology contained in a specific file.", click "continue", and browse to the CBO file, select it, click then click "Open" then "Continue" then "Finish". Protégé will import CBO into the workspace. Using the same steps load the Unit Ontology. Change to the "Classes" <tab> and the top levels of both CBO and UO should be visible.


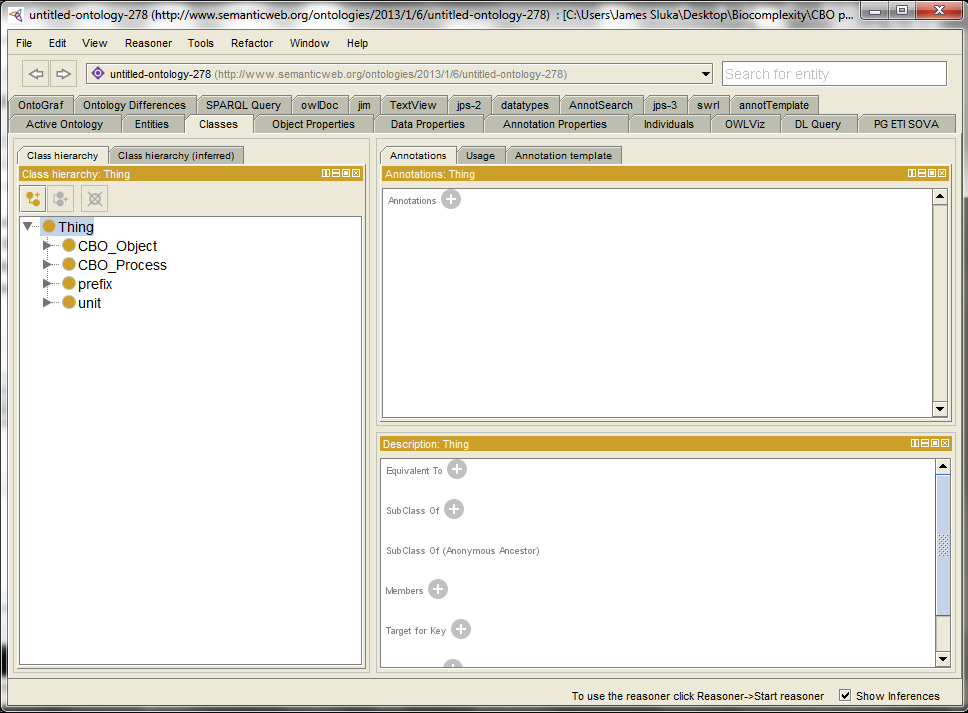


## Define the System

We can now begin to define the model. First we need to define the basic system that is being modeled. We create an OWL:Individual of the class "CBO:System" for our model.

**HINT:** As a quick way of navigating to the "CBO:System" class enter "sys" in the search box in the upper right of the Protégé 4 window and select "System" from the dropdown menu. You can also use wildcards in this box, for example "*cell*" will return a list of all the classes with "cell" anywhere in the class name. In situations when you can't remember (or don't know) what class you need you can also search the annotations for the classes using the AnnotSearch tab.

Move to the "Individuals" tab. In the "Individuals by type" box click the triangle+ symbol and give the individual the name "TumorWithAngiogenesis". In the "Description" window click the "+", then the "Class Hierarchy" tab, navigate to the "System" class, click and then click "OK".

It is a good idea to add suitable annotations to our ontology for this specify model. Move to the "Classes" tab, click "Thing" in the "Class Hierarchy" window (annotations for the entire ontology a part of the top level object which is "Thing") and click the "+" in the annotation window to add an annotation to the "Thing" class. Add an annotation of type "created_by" with your name, "creation_date", and "description" (such as " OWL:CBO description of a Compucell3D model of tumor growth with angiogenesis.").

We now need to define the system qualities such as the models extent, modeling platform etc. To define the models extents create an individual of the class "DiscreteExtent" (the model is defined on a lattice versus a continuous measurement system) named "TumorWithAngiogenesis_extents" (the actual names of the individual is not critical). In the "Description" window click the "+" and add the type "DiscreteExtent". We now need to link this individual to the actual values for our model. In the CC3D scripts the model is defined as being 50x50x80. Select the individual "TumorWithAngiogenesis_extents" and add the annotation (of type "comment") "These are the X, Y and Z extents of the box defining the model's universe." Now add the value to the individual by selecting the "+" for "Data Property Assertions" in the "Property Assertions" frame, then select "hasExtentValueSet3D" then enter "50,50,80" in the value box and click OK.


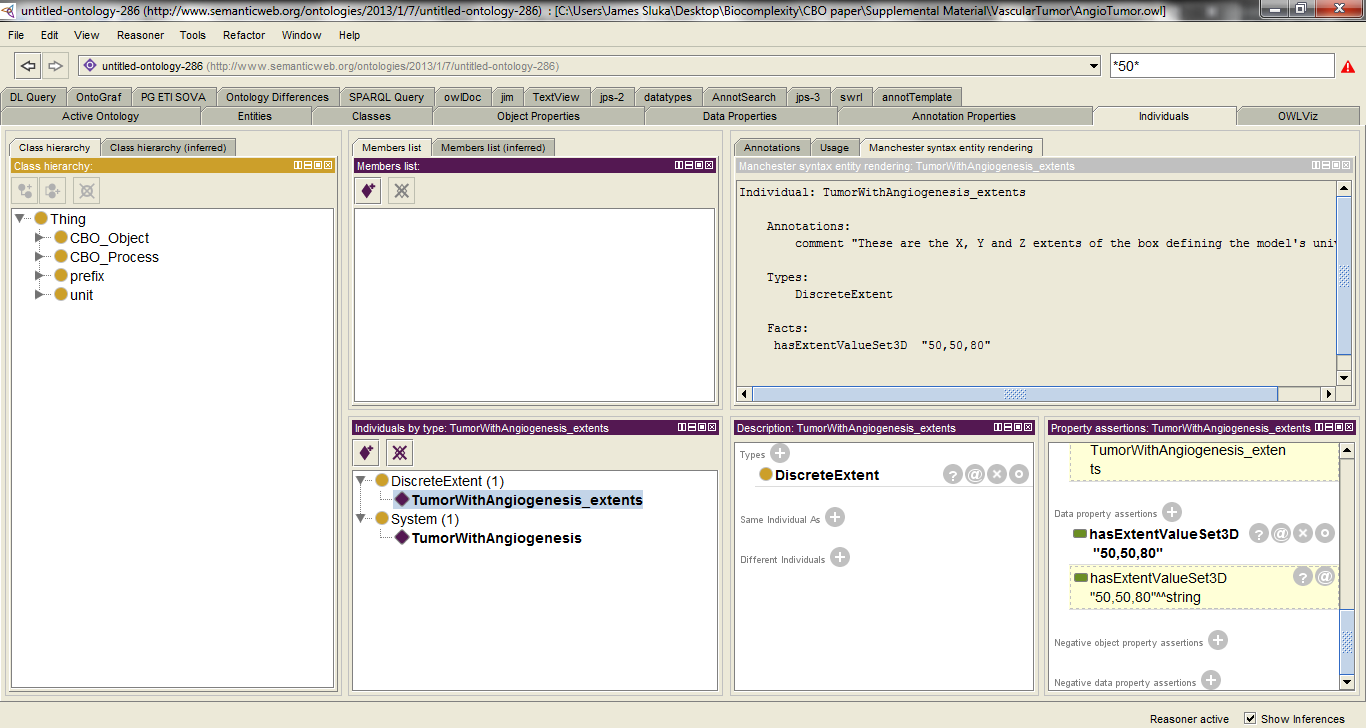


We also need to specify that the individual TumorWithAngiogenesis_extents is a quality of the individual of type system named TumorWithAngiogenesis. Select TumorWithAngiogenesis in the Individuals list then the clock on the "+" for "Data Property Assertions" in the "Property Assertions" frame, then select has_Quality form the property list and TumorWithAngiogenesis_extents from the individuals list.


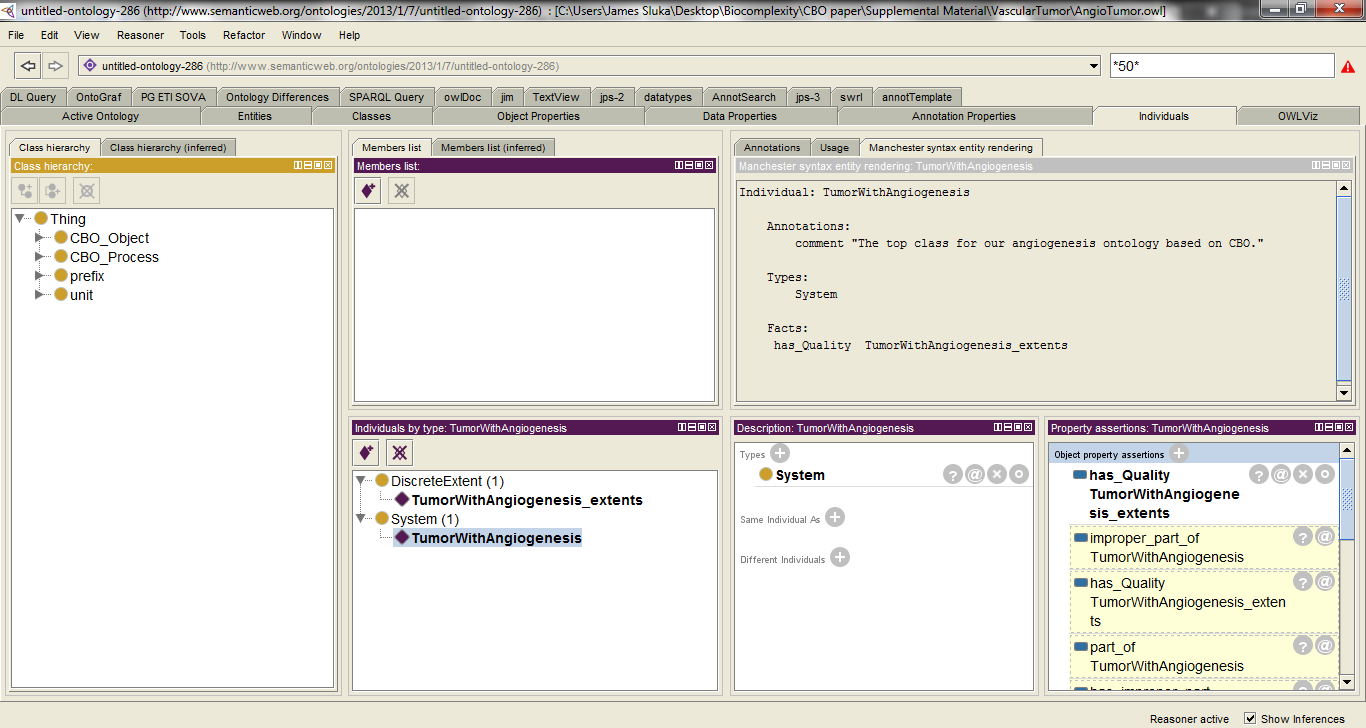


The units of the extents also need to be defined. CompuCell3D models are defined on an integer lattice of voxels (or pixels). The extent value of "50,50,80" is in voxels. According to the CC3D script a voxel is a cube that is 4um on edge. We need to specifiy that conversion factor with an individual of type SystemPixel2Distance. Create an individual named "TumorWithAngiogenesis_pixel2dist" of the class SystemPixel2Distance and in the Description window add the Type " SystemPixel2Distance". In the "Properties assertions" frame click the "+" for "Data property assertions" and select hasFloatValue as the property (in general this scale factor is a floating point number) and enter 4 as the value, in the Type selection (bottom right) set the data type to "float", then click OK We also need to assert that "TumorWithAngiogenesis_pixel2dist" is a quality of our top level individual TumorWithAngiogenesis. Select the individual TumorWithAngiogenesis then add the Object property assertion "has_Quality TumorWithAngiogenesis_pixel2dist". We can check that things are organized corretly by looking at the "Manchester syntax entity rendering". The individual TumorWithAngiogenesis is below. Notice also the two has_Quality links in the bottom right frame.


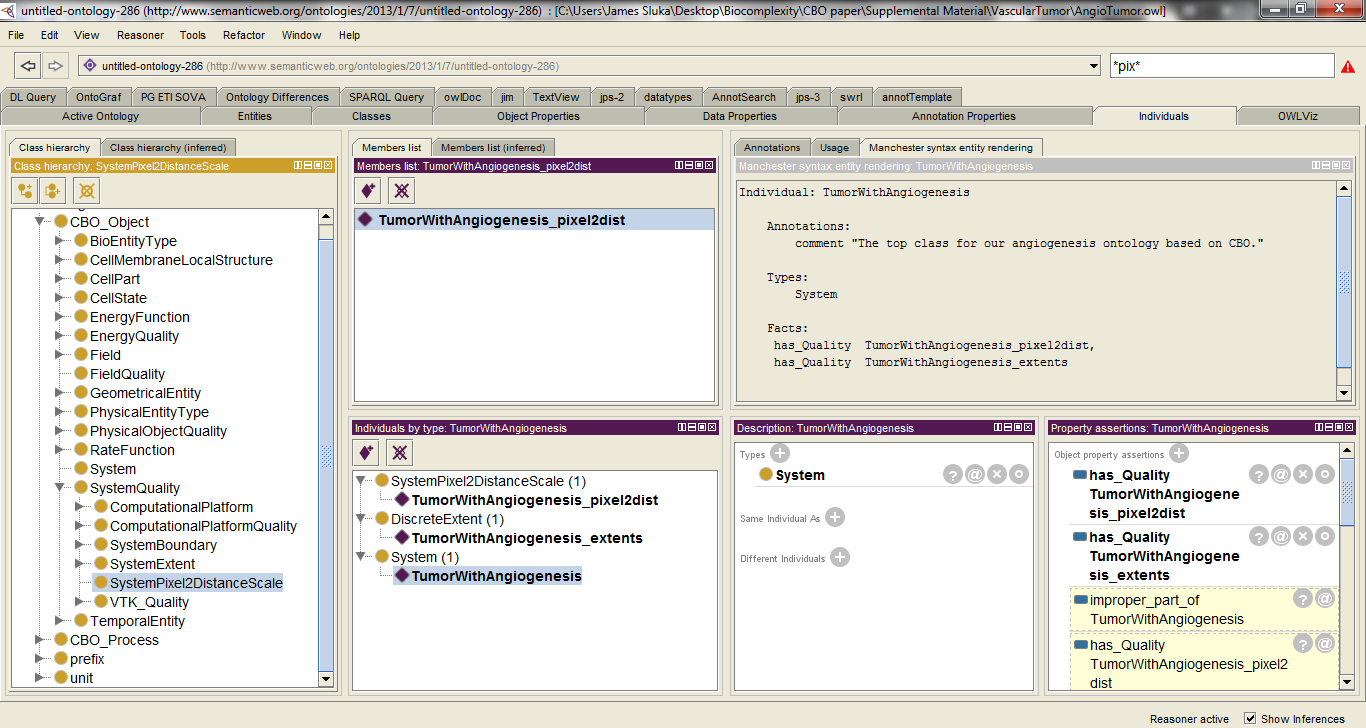


The Manchester rendering frame for the "TumorWithAngiogenesis_pixel2dist" individual:


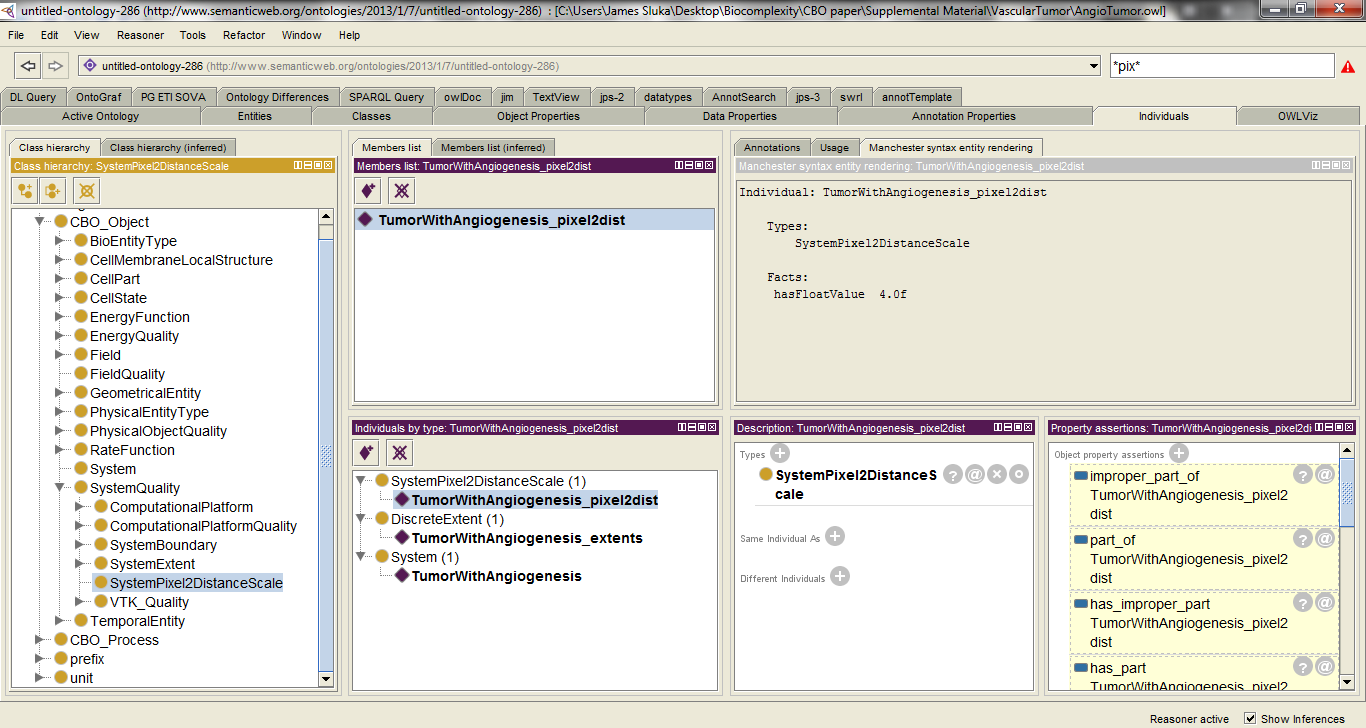


The Manchester rendering frame for the "TumorWithAngiogenesis_extents" individual:


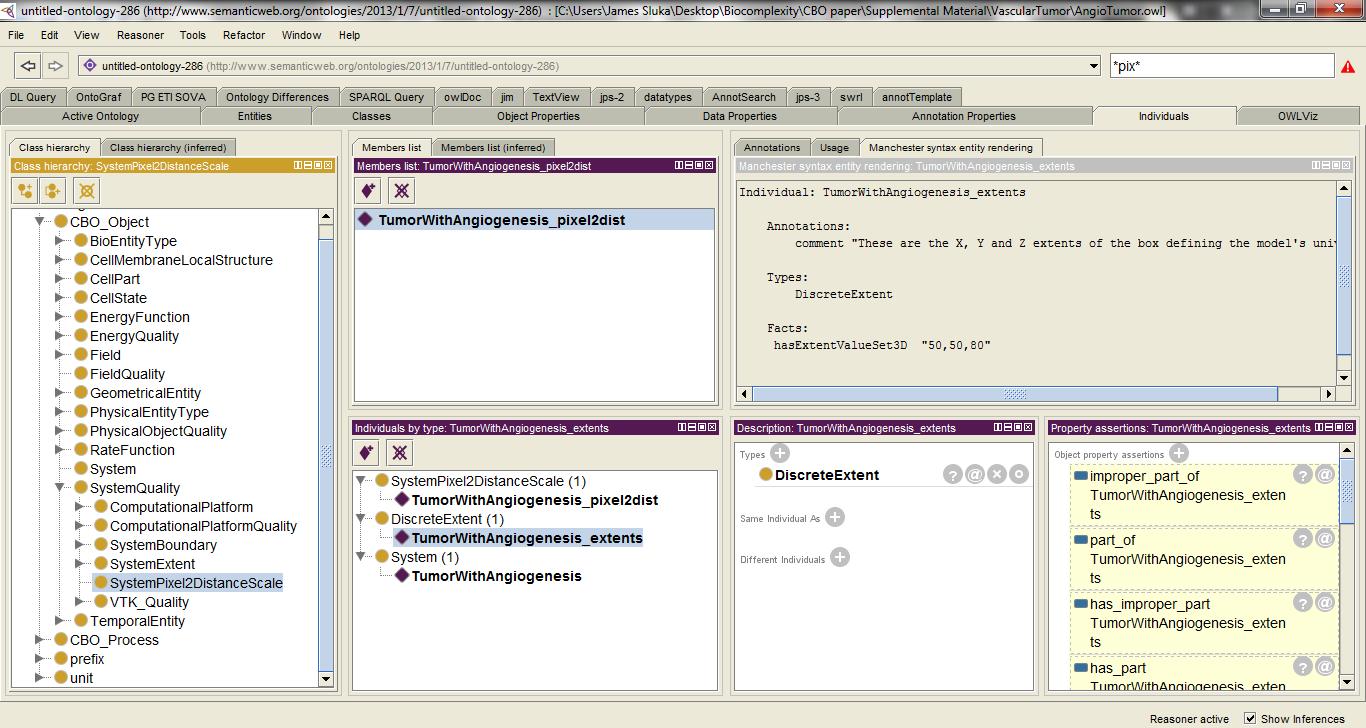


To define the conversion of simulation steps to simulated time we proceed in a similar fashion. Create an individual of class VTK_FileTimeStep named TumorWithAngiogenesis_VTKtimestep. The CC3D script indicates "1 MCS ~ min (typical)" so we assign a data property to TumorWithAngiogenesis_VTKtimestep with hasFloatValue of 1 (of type "float"). To assign a unit to this value we also make the individual TumorWithAngiogenesis_VTKtimestep of type "minute" (defined in the "unit" tree of the unit ontology that we preloaded into our ontology). We also link this individual to our top level individual TumorWithAngiogenesis via has_Quality. The Manchester syntax for TumorWithAngiogenesis_VTKtimestep is:

Individual: TumorWithAngiogenesis_VTKtimestep

Types:

minute,

VTK_FileTimeStep

Facts:

hasFloatValue 1.0f

Our top level indvidual is now, in Manchester syntax:

Individual: TumorWithAngiogenesis

Annotations:

comment "The top class for our angiogenesis ontology based on CBO."

Types:

System

Facts:

has_Quality TumorWithAngiogenesis_pixel2dist,

has_Quality TumorWithAngiogenesis_extents,

has_Quality TumorWithAngiogenesis_VTKtimestep

We now define the computational platform that this particular model is designed for. Add an individual of class Compucell3D called TumorWithAngiogenesis_platform then add a has_Quality link from the individual TumorWithAngiogenesis to TumorWithAngiogenesis_platform.

To specify the behavior of the model at the model's bounding box, to match the periodic boundary conditions in the CC3D model, create individuals of SystemPeriodicX, SystemPeriodicX, and SystemPeriodicX named TumorWithAngiogenesis_Xbound, TumorWithAngiogenesis_Ybound, and TumorWithAngiogenesis_Zbound. Now link those individuals to the main system individual (TumorWithAngiogenesis) via has_Quality. The Manchester Rendering for our main individual TumorWithAngiogenesis now is:

Individual: TumorWithAngiogenesis

Annotations:

comment "The top class for our angiogenesis ontology based on CBO."

Types:

System

Facts:

has_Quality TumorWithAngiogenesis_pixel2dist,

has_Quality TumorWithAngiogenesis_platform,

has_Quality TumorWithAngiogenesis_extents,

has_Quality TumorWithAngiogenesis_Ybound,

has_Quality TumorWithAngiogenesis_VTKtimestep,

has_Quality TumorWithAngiogenesis_Xbound,

has_Quality TumorWithAngiogenesis_Zbound

**For completeness, and to facilitate interpretation, sharing and reuse buy others it is important to link biological concepts to GO, cell types to CL, small molecules to ChEBI, proteins to SwissProt etc.**

## Define the Cell Prototypes

Now that the basic system definitions are in place we can move on to the definition of the cell prototypes. Cell prototypes are new classes that below the CBO:Cell class. The CC3D script contains the definition of four cell types plus a type for "Medium";

<Plugin Name="CellType">

<CellType TypeName="Medium" TypeId="0"/>

<CellType TypeName="Proliferating" TypeId="1"/>

<CellType TypeName="Necrotic" TypeId="2"/>

<CellType TypeName="Vascular" TypeId="3"/>

<CellType TypeName="NeoVascular" TypeId="4"/>

</Plugin>

We will create classes for each of these cell types named as they are in the CC3D script. In addition, we will create mappings from the cell prototypes to the TypeIDs in the CC3D script. To create the prototype class for "Proliferating" go to the Protégé Classes <tab> then search for Cell and select "Cell" from the dropdown. In the classes panel ("Cell" should be selected) click on the "Add Subclass" button (
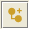
) and name the new class "Proliferating". Note that Protégé shows this class in bold face in the Class frame indicating that this class was created in this Ontology as opposed to being imported from CBO or the Unit ontologies. To quickly annotate this new class switch to the "Annotation Template" <tab> (make this <tab> visible via Windows – Tabs –AnnotTemplate) and enter the basic annotations such as:


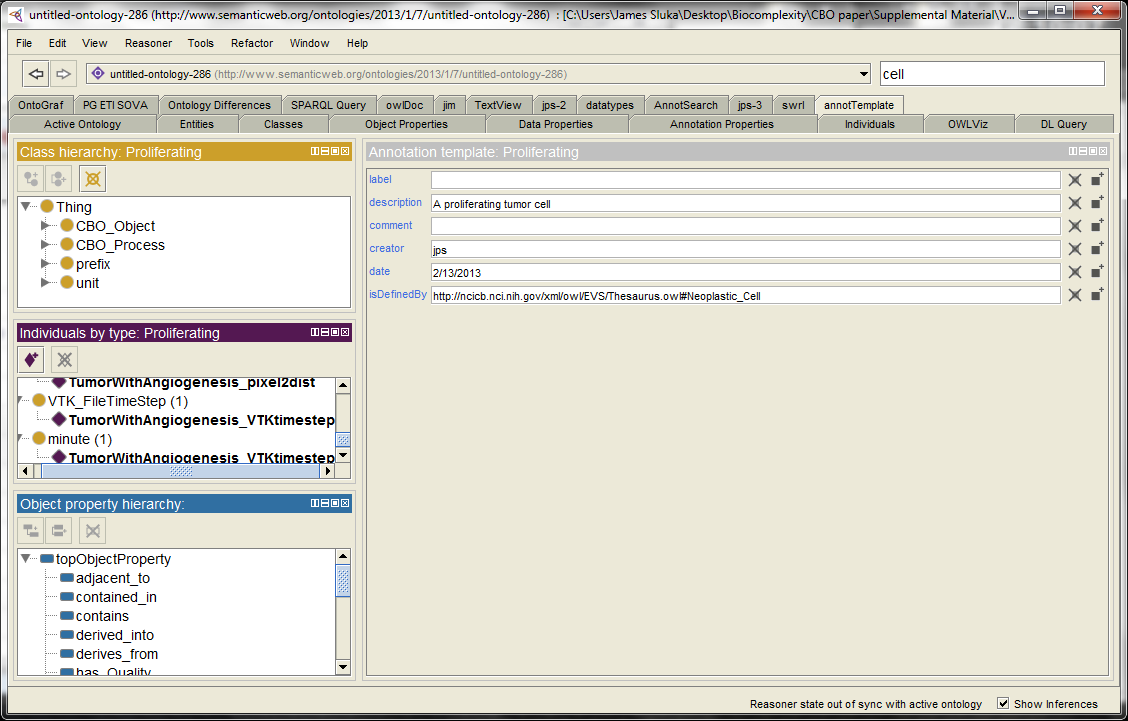


Note the "idDefinedBy" annotation. This points at an external ontology, the NCI thesaurus in this case, that defines our proliferating tumor cell as a Neoplastic cell. In general cells should be annotated using a suitable external ontology such as the NCI Thesaurus or a cell line ontology like Cell Ontology (CL). A handy tool for searching a large number of bio-ontologies for suitable terms is at <http://bioportal.bioontology.org/search>. In some cases you might link to multiple external ontologies, to do that click on the add annotation icon (
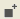
) in the Annotation template window.


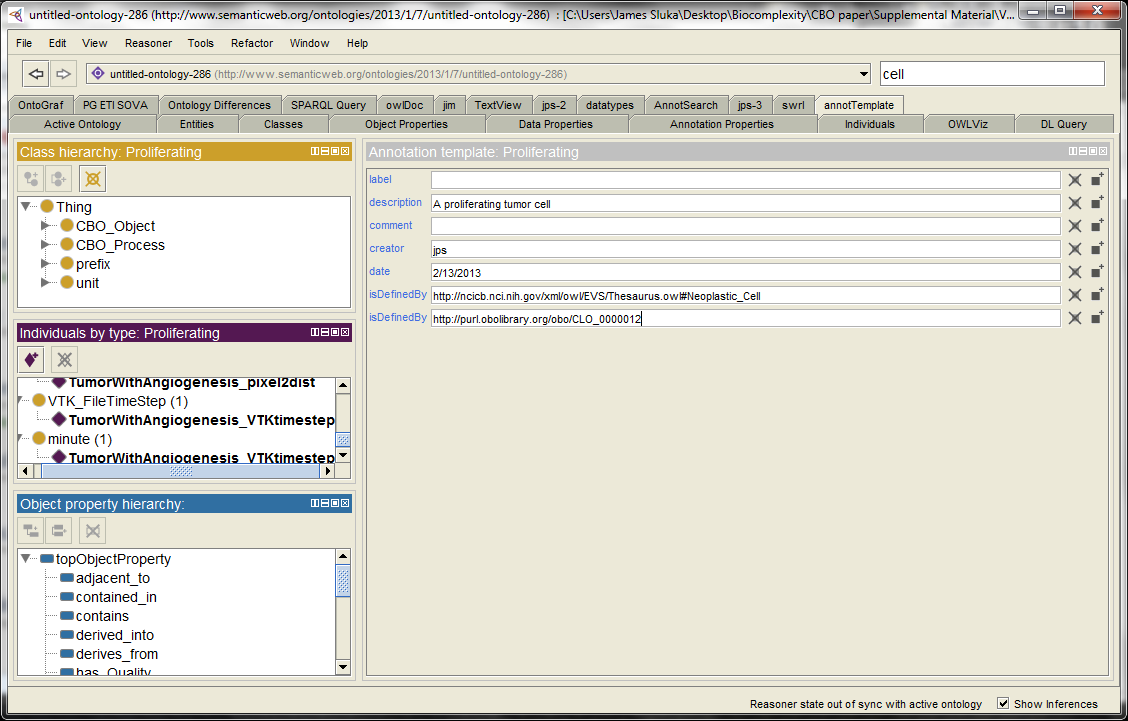


We now want to add the cell type numeric ID. This numeric ID is used by CC3D and is also the cell type ID that will be used in the VTK files. Switch to a Classes view and click on the + for "Equivalent to". In the popup window select the "class expression editor" <tab> and enter "hasVTKtypeID value 1" (without the quotes).

The class expression editor has autocomplete. If you enter "hasV" and then a <tab> the available entities will be shown (in this case hasVTKtypeID and hasVTKfieldID). If you haven’t typed anything, or if the cursor is at the end of the line, then the available options are shown in those cases as well.

Once one cell prototype has been defined you can copy it (or more generally you can copy any class) via the menus or with <ctrl><shift>c. Protégé will ask for a name for the copied class and the new class will inherit all of the features of the original class such as annotations, sub-class relations etc.

Once we are happy with the definition of this first cell prototype we can save some typing by initially creating additional cell prototypes as copies of the first "proto-prototype" followed by changing the details for the new prototypes. Copy Proliferating to "Necrotic", "Vascular", "NeoVascular" and "Medium". Switch to the AnnotTemplate view and correct the annotations then switch to the class view and correct the hasVTKtypeID values.

We also need to specify that the four "cells" are of type PhysicalEntityType:CorpusclarEntity but Medium is of type PhysicalEntityType:ContinousEntity and PhysicalObjectQuality:Fill. To do this switch to a class view, select, for example, the Necrotic cell class then add "Subclass Of", switch to the "class hierarchy" <tab> and select PhysicalEntityType:CorpusclarEntity. Do the same for "Medium" but add ContinousEntity and Fill instead of CorpusclarEntity. If you synchronize the reasoner the prototype classes will be listed in their proper places and in general each prototype will occur in more than one place in the hierarchy.

Finally, we need to tag the cell states for the prototype classes. The "Necrotic" cell class is a subclass of CellState:isNecrotic, the "NeoVascular" and "Proliferating" cell classes are subclasses of CellState:isGrowing so add those "SubClass of" relationships.


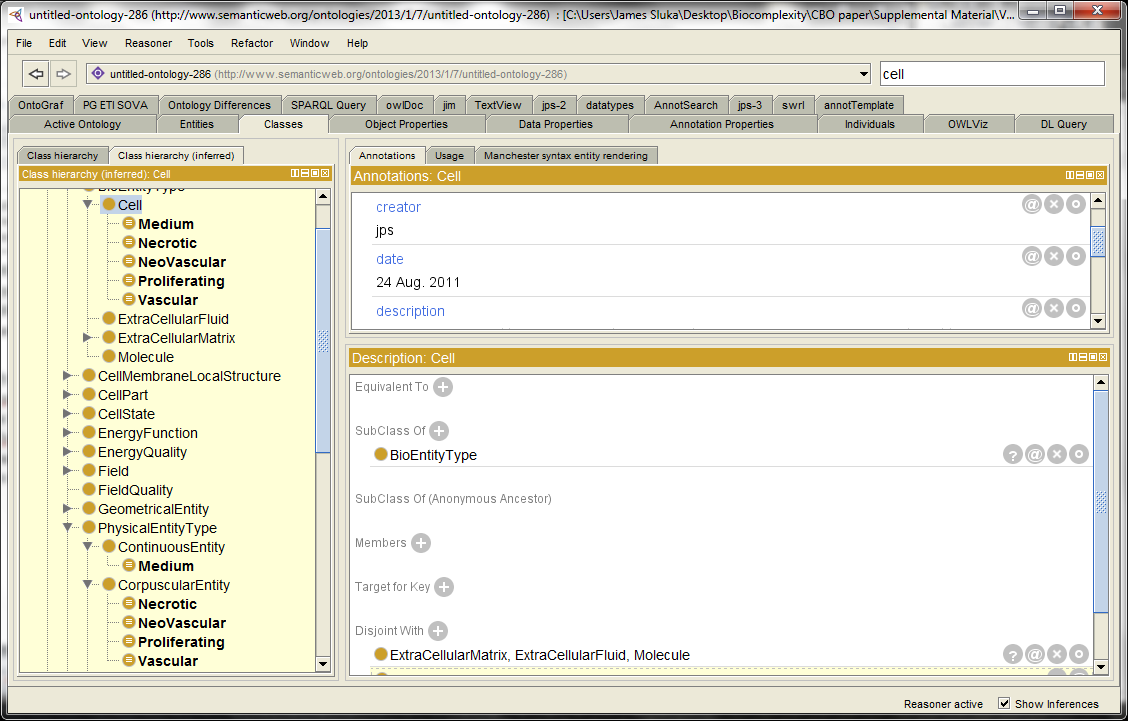


If we now check the Manchester renderings we get for Necrotic cells:

Class: Necrotic

Annotations:

date "2/13/2013",

creator "jps",

description "A necrotic tumor cell.",

isDefinedBy "http://ncicb.nci.nih.gov/xml/owl/EVS/Thesaurus.owl#Necrotic",

isDefinedBy "http://purl.obolibrary.org/obo/PATO_0000647"

EquivalentTo:

hasVTKtypeID value 2

SubClassOf:

CorpuscularEntity,

IsNecrotic,

Cell

And for Vascular cells:

Class: Vascular

Annotations:

date "2/13/2013",

creator "jps",

description "A vascular portion. Note that this \"cell\" type represents a portion of a

capilary including both the endothelium and the lumen.",

isDefinedBy "http://purl.obolibrary.org/obo/FMA_63194"^^anyURI,

isDefinedBy "http://sig.uw.edu/fma#Capillary",

isDefinedBy "http://ncicb.nci.nih.gov/xml/owl/EVS/Thesaurus.owl#Capillary"

EquivalentTo:

hasVTKtypeID value 3

SubClassOf:

CorpuscularEntity,

Cell

Issue: Should annotate the FMA_63194 annotation with the class name "capillary".

## Define the Processes

Now that the basic system definition and cell types are in place we can move on to the definition of the cell processes. Cell processes are new classes below the CBO_Process:CellProcess class.

### Cell Growth

The CC3D Python script contains a steppable that controls the volumetric growth of the cells in the simulation (class VolumeParamSteppable(SteppablePy)). Growth processes are defined for cell type IDs of 1, 2 and 4, corresponding to cell type names of "Proliferating", "Necrotic" and "NeoVascular". Note that for the "Necrotic" cell type the growth rate is negative and these cells shrink and eventually vanish.

We will create new classes for each of the cell growth processes described in the CC3D script. It is helpful, but not required, to name these new classes in some consistent fashion. We will use the cell type name and the string "Growth". So the CBO class for the growth of "Proliferating" will be called "Proliferating_Growth". As in the case of creating cell prototypes, the growth prototypes are similar and we will take advantage of that by creating an initial, well annotated growth class then copying that class.

Switch to a class view tab, enter "*growth" in the Protégé search box then select CellGrowth from the dropdown list. In the class window click the add subclass button (
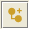
) and name the new class Proliferating_Growth. Switch to the Annotation Template tab and annotate this class;


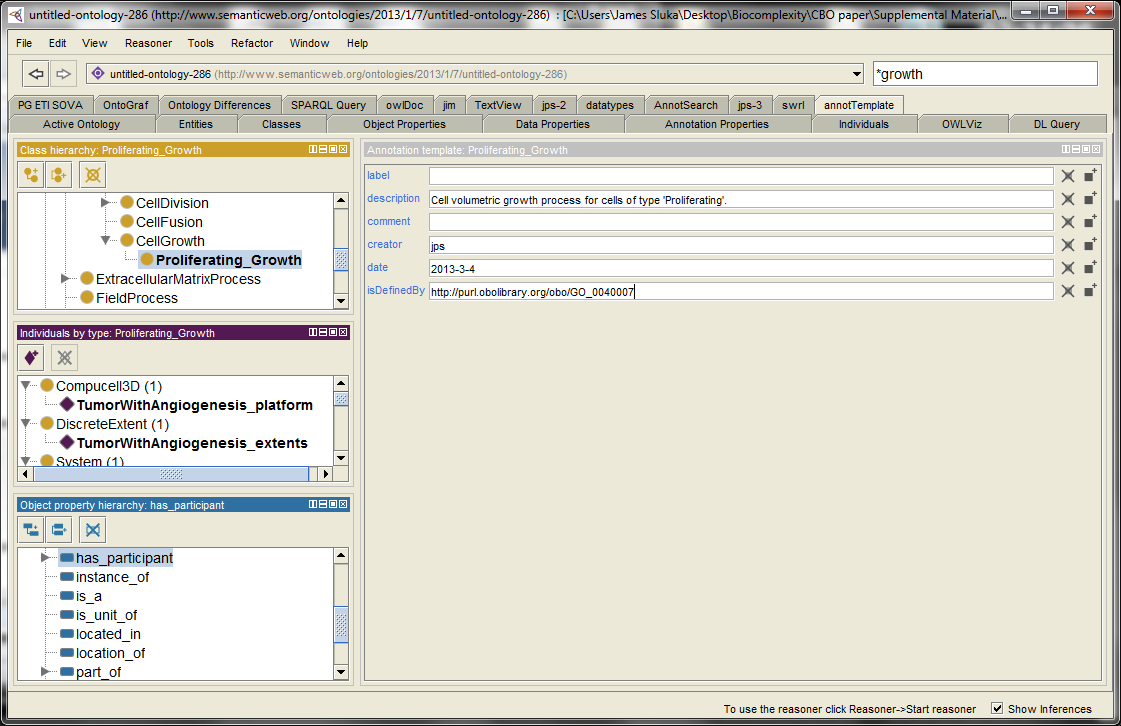


We now need to specify that this growth process is specific to the "Proliferating" cell prototype class. Switch to the Classes tab, select the Proliferating_Growth class and then in the description window click the "+" to add a "SubClass Of". In the pop-up window select the Class Expression Editor tab then type "has_P" then a tab character and select "has_participant". Next type "exa" then a tab character ("exa" will be replaced by "exactly"), type a space then a 1, followed by a space and "Proliferating".


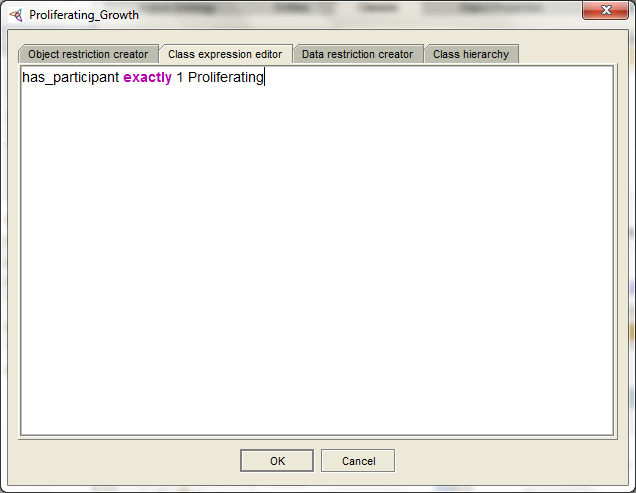


The class view is now:


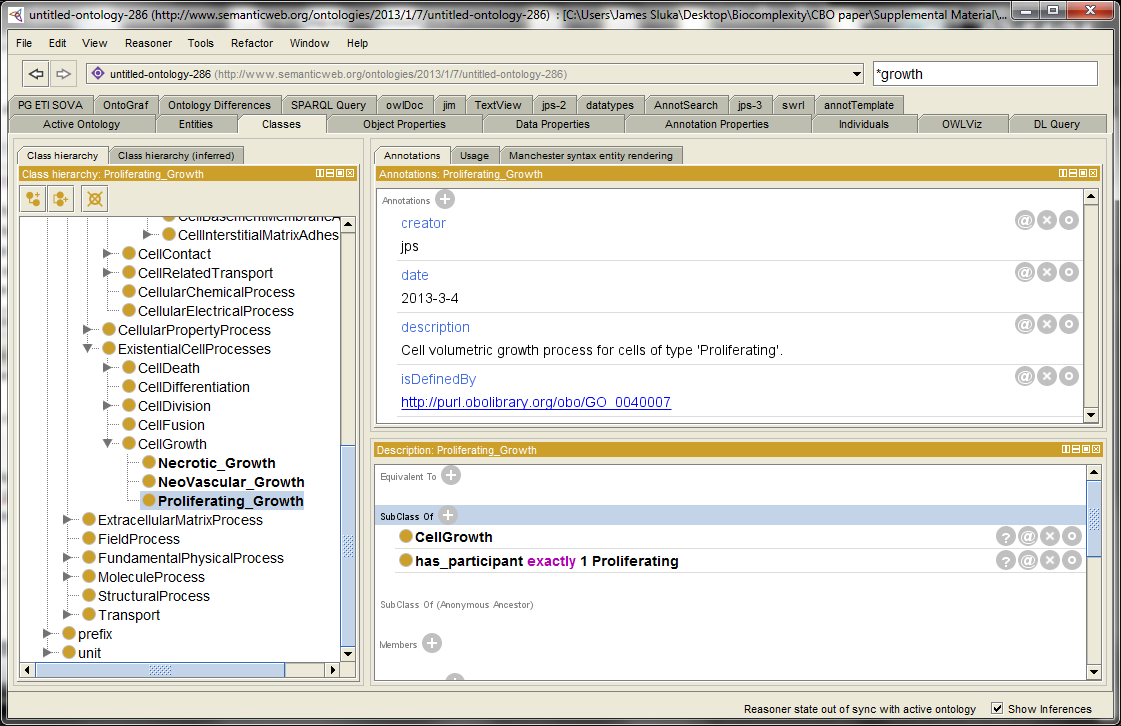


Now duplicate this prototype growth process and give the new version the name "NeoVascular_Growth", then make another copy for the "Necrotic" cells called "Necrotic_Growth". We now need to correct the annotations for the two new classes. For the NeoVascular_Growth change the description:


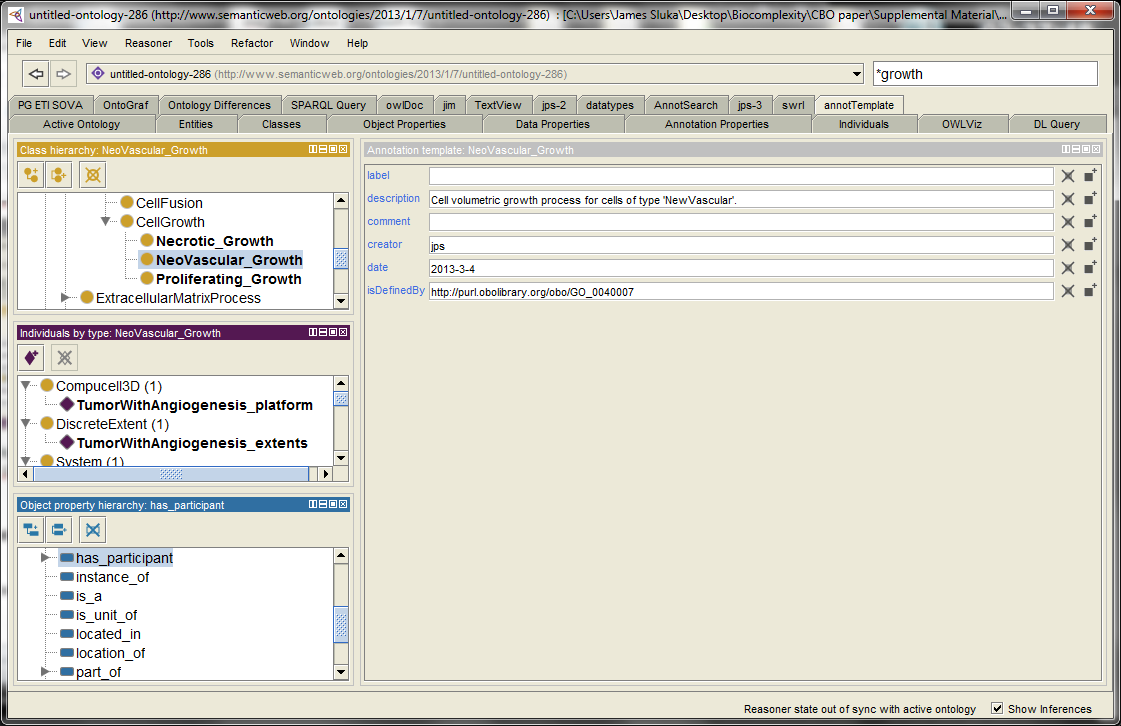


For the "Necrotic_Growth" we need to change both the description and the idDefinedBy link:


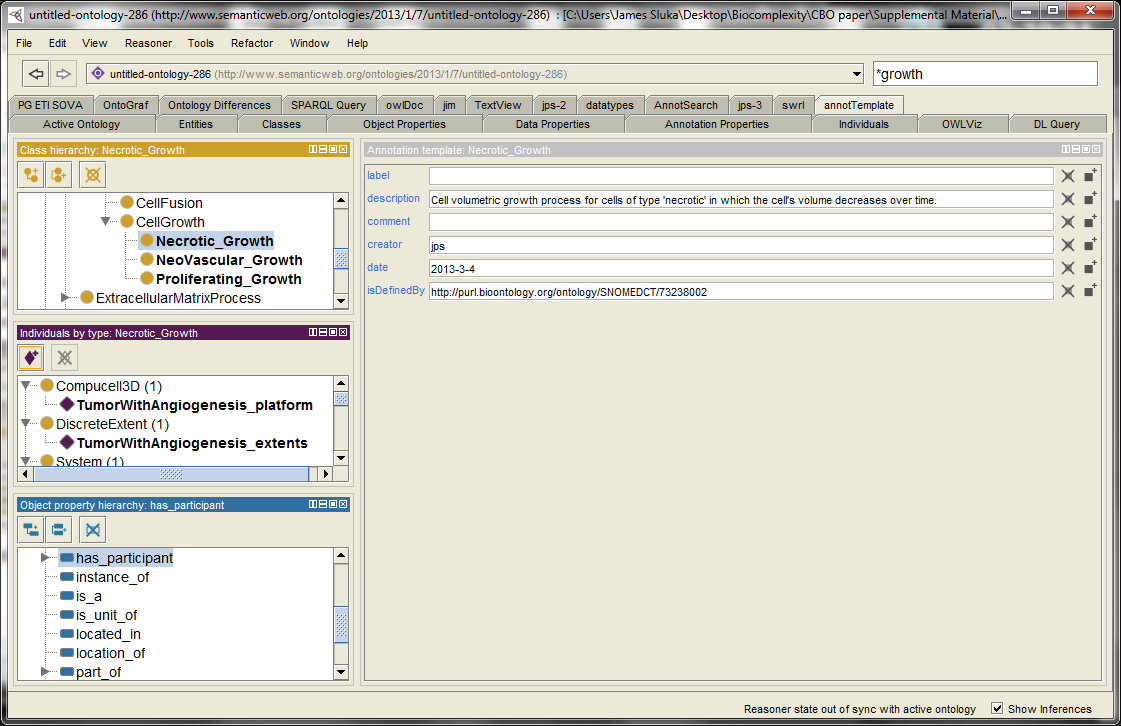


The SNOMEDCT/73238002 term is "shrinkage". We can annotate the SNOMEDCT annotation with this human-readable version from the class tab by clicking on the
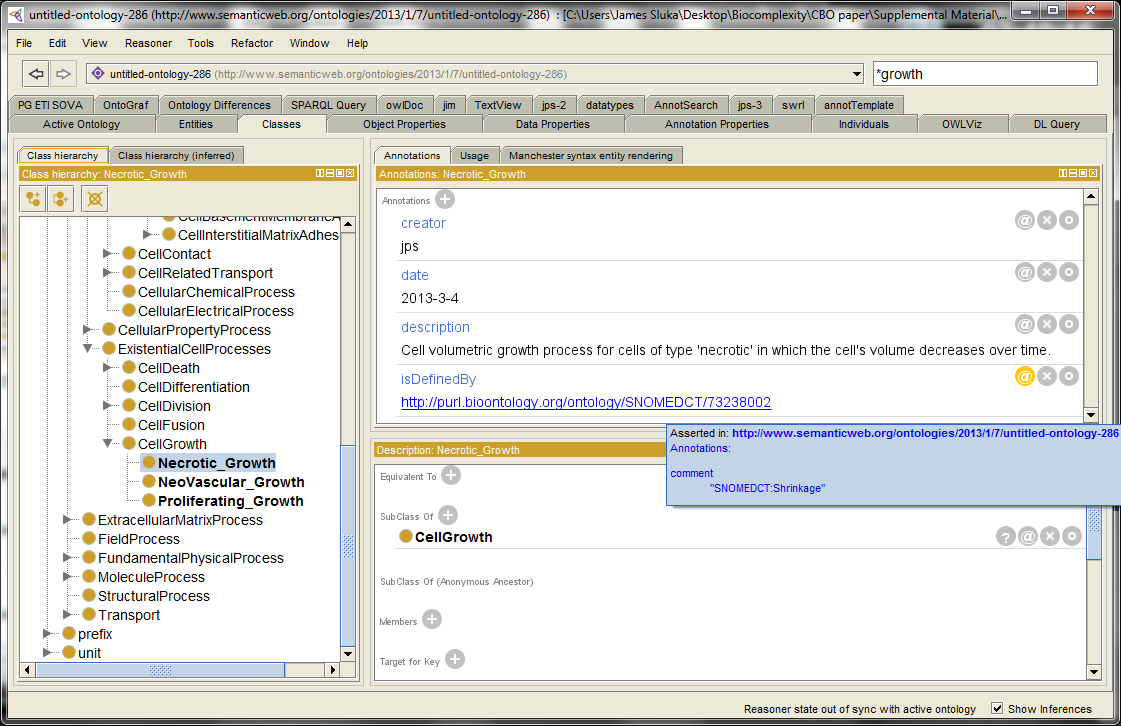
 button in the Annotations window, then selecting the "+" icon in the pop-up window followed by selecting "Comment" and typing in "SNOMEDCT:Shrinkage" followed by the "OK" button. In the Classes tab when we mouse-over the isDefinedBy annotation we get a pup-up with the annotation for the annotation:


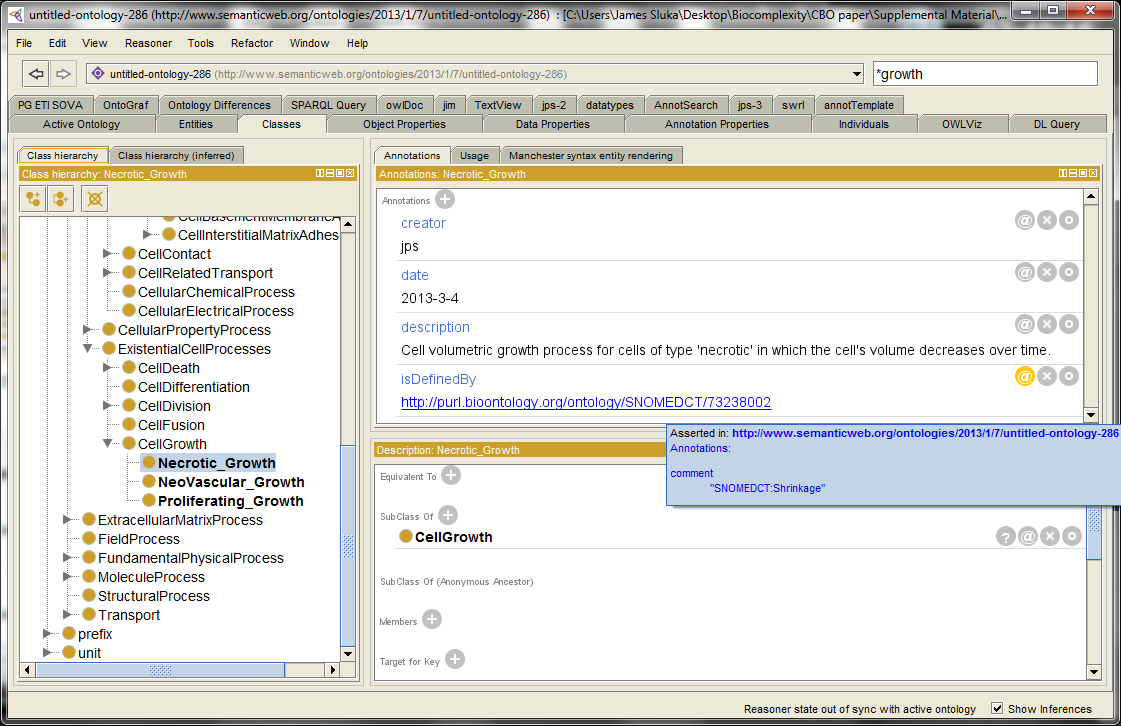


We also need to correct the "SubClass Of" fields for the two new classes. Select the class NeoVascular_Growth in the Class tab then select the edit button in the description window adjacent to the incorrect "SubClass Of" relation:


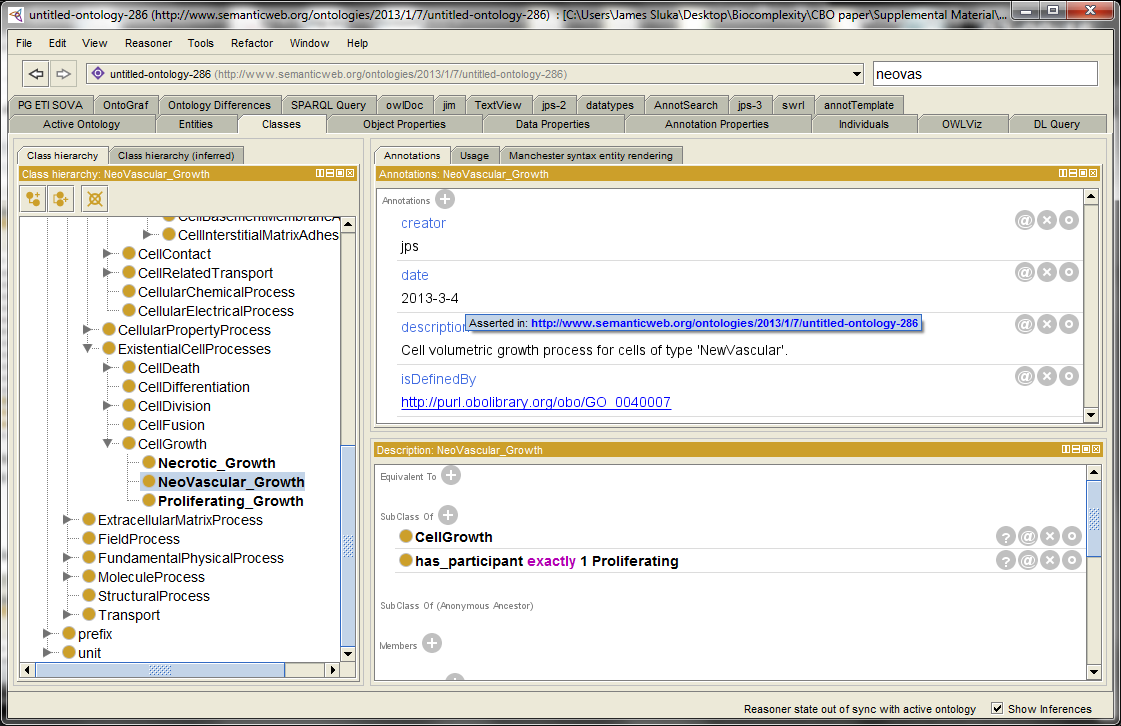


edit button

In the pop-up window replace "Proliferating" with "NeoVascular".

The NeoVascular class view should now be:


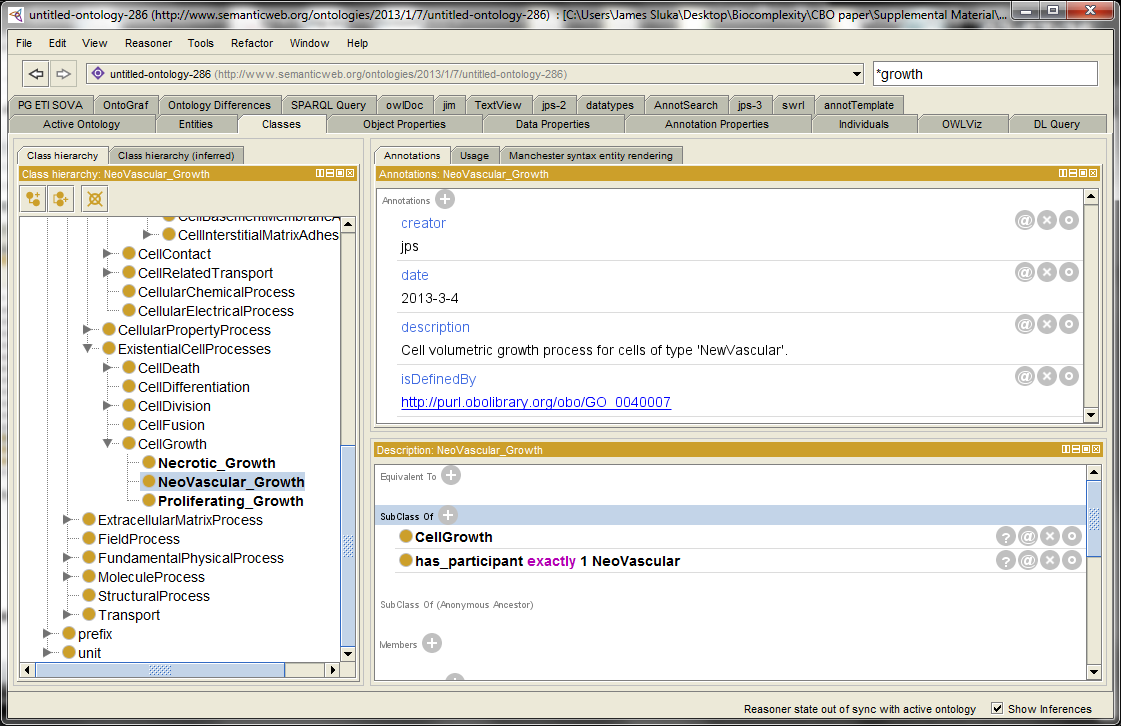


Now correct the Necrotic_Growth class "SubClass of" in a similar fashion.

The Manchester rendering of the Necrotic_Growth class is now:

Class: Necrotic_Growth

Annotations:

date "2013-3-4",

creator "jps",

description "Cell volumetric growth process for cells of type 'necrotic' in which the

cell's volume decreases over time.",

Annotations: comment "SNOMEDCT:Shrinkage"

isDefinedBy "http://purl.bioontology.org/ontology/SNOMEDCT/73238002"

SubClassOf:

has_participant exactly 1 Necrotic,

CellGrowth

### Cell Death

The C3D Python script contains code that converts a cell of type "Proliferating" into a cell of type "Necrotic" if the glucose level of the cell drops below a threshold:
In the class VolumeParamSteppable(SteppablePy)):

#Proliferating Cells

if cell.type == 1:

...

# Proliferating Cells become Necrotic when GlucoseConcentration is low

if GlucoseConcentration < 0.001 and mcs>1000:

cell.type = 2

This is a phenotypic change in the cell's type. We need to create a model-specific class for this process. Switch to the Class tab and in the search box type "Pheno" then select PhenotypicChange from the dropdown. In the Class Hierarchy window (PhenotypicChange should be highlighted) click on the add subclass button (
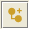
)) and name the new class "Proliferating_2_Necrotic". Switch to the Annotation Template and annotate this new class. I used the Go term (http://purl.obolibrary.org/obo/GO_0070265) "necrotic cell death" and the SNOMEDCT term (http://purl.bioontology.org/ontology/SNOMEDCT/6574001) "Necrosis".

We need to link the necrosis class to the cell source type and the cell target types. Switch to the Class tab and select the Proliferating_2_Necrotic class (or search for it in the search box). Click on the "+" for "SubClass Of" in the Description view. Select the Class Expression tab in the pop-up window and enter:
 has_participant some Proliferating
 and derived_into some Necrotic
(or use the auto complete function). Note that "derived_into" is a transitive property and the OWL reasoners won't allow a cardinality restriction such as "exactly 1", hence the use of the OWL construct "some". The Class tab view should now be similar to:


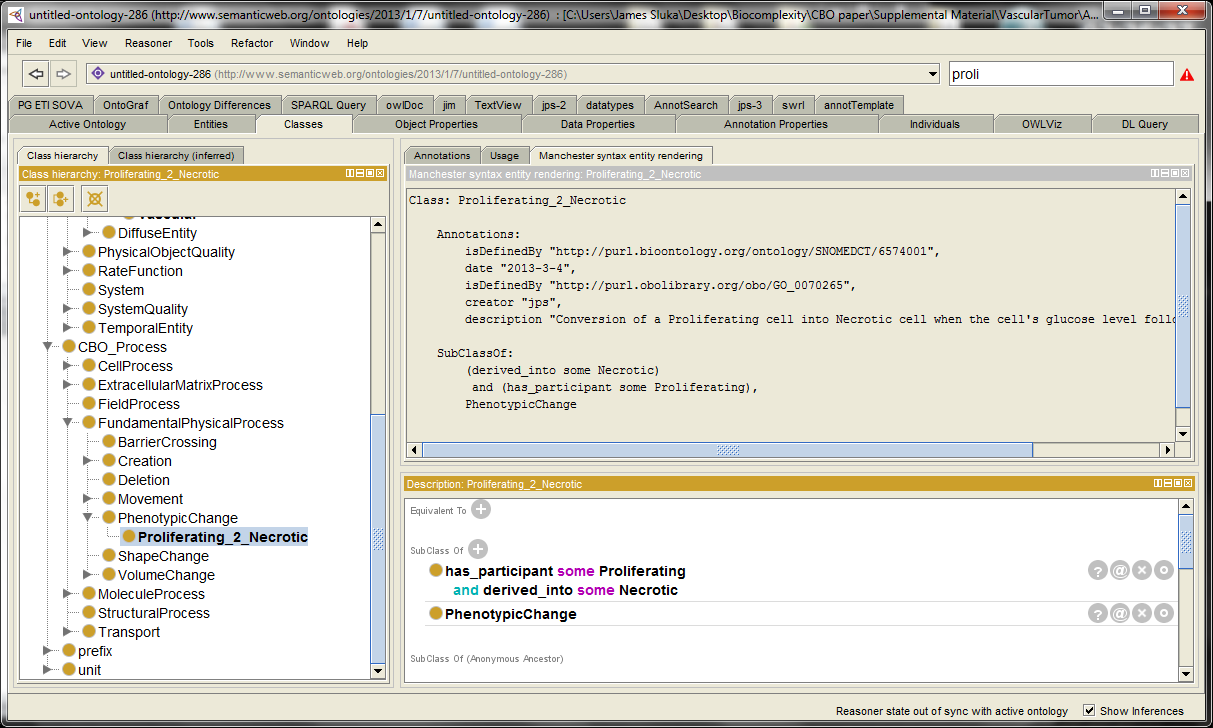


### Cell Division (Mitosis)

Cell proliferation (division, mitosis) is defined in the CC3D script's "MitosisSteppable" CC3D/Python class:

class MitosisSteppable(MitosisSteppableBase):

def __init__(self,_simulator,_frequency=1):

MitosisSteppableBase.__init__(self,_simulator, _frequency)

def step(self,mcs):

cells_to_divide=[]

for cell in self.cellList:

if cell.type == 1 and cell.volume>64:

cells_to_divide.append(cell)

if cell.type== 4 and cell.volume>128:

cells_to_divide.append(cell)

The above code indicates that cells of type ID 1 ("Proliferating") and type ID 4 ("NeoVascular") divide when their volume exceeds a cell type specific thresholds. Note that in CC3D, and many other multi-cell modeling platforms, cell "growth" and cell "mitosis" (division) are separate processes. Some ontologies use a single name to describe both processes and the two processes are considered to always happen together. Here they are described as distinct processes.

We will create new classes for each of the cell division processes described in the CC3D script. It is helpful, but not required, to name these new classes in some consistent fashion. We will use the cell type name and the string "Mitosis". The CBO model-specific class for the mitosis of "Proliferating" cells will be "Proliferating_Mitosis". As in the case of creating cell prototypes, the mitosis prototypes are similar and we will take advantage of that by creating an initial, well annotated mitosis class then copying that class to create the other cell division classes.

Switch to a class view tab, enter "mit" in the Protégé search box then select "Mitosis" from the dropdown list. Note that the CBO has four subclasses for Mitosis:

1. AsymmetricCellDivisionOrientedCleavagePlane
2. AsymmetricCellDivisionRandomCleavagePlane
3. SymmetricCellDivisionOrientedCleavagePlane
4. SymmetricCellDivisionRandomCleavagePlane

For this CC3D model the cell divisions are defined as symmetric cleavages (both resulting cells are of the same type and each is one half the volume of the original cell) and the cleavage plane is randomly oriented. Select SymmetricCellDivisionRandomCleavagePlane in the Class hierarchy then in the class window click the add subclass button (
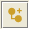
) and name the new class Proliferating_Mitosis. Switch to the Annotation Template tab and annotate this class;


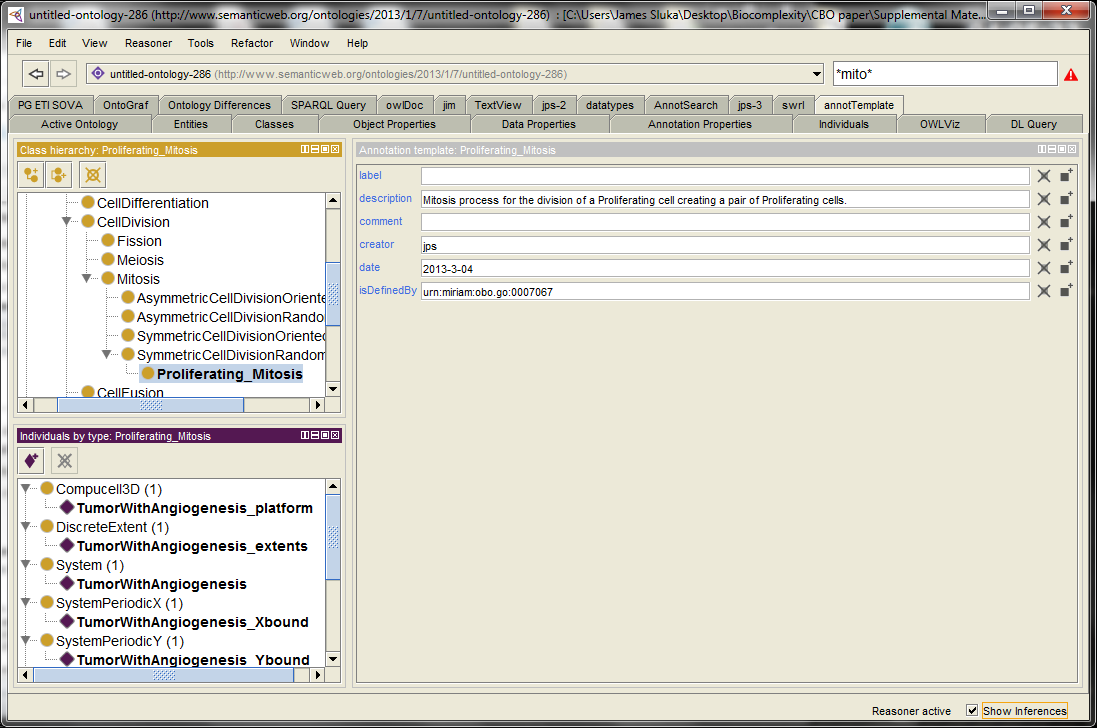


Switch back to the class view and add the "SubClass Of" restriction:
 (has_participant some Proliferating)
 and
 (derived_into some Proliferating)

As previously we would prefer to use cardinality restrictions such as;
 (has_participant exactly 1 Proliferating)
 and
 (derived_into exactly 2 Proliferating)

but those are problematic with transitive properties like "derived_into".


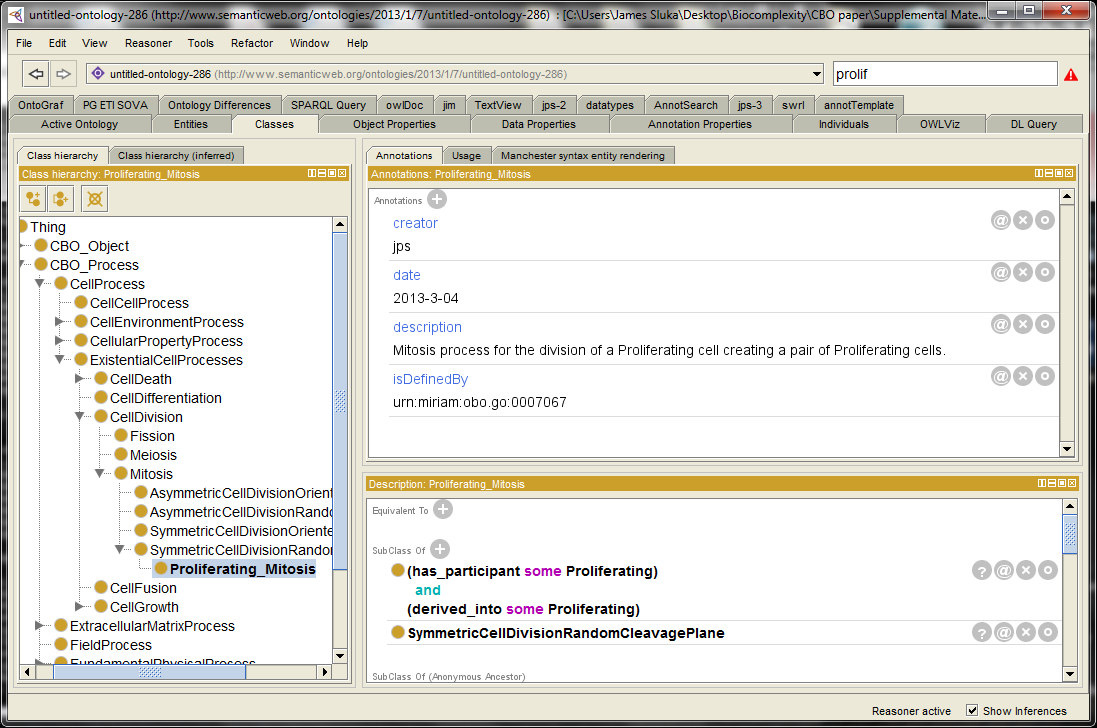


To create the mitosis class for the NeoVascular cells start by copying the Proliferating_Mitosis class with <ctrl><shift>c and name the new class "NeoVascular_Mitosis". Correct the "SubClass Of" relations to:
 (has_participant some NeoVascular)
 and
 (derived_into some NeoVascular)

In addition, since this mitosis processes is also an example of angiogenesis it should be annotated as such using external ontologies such as GO.


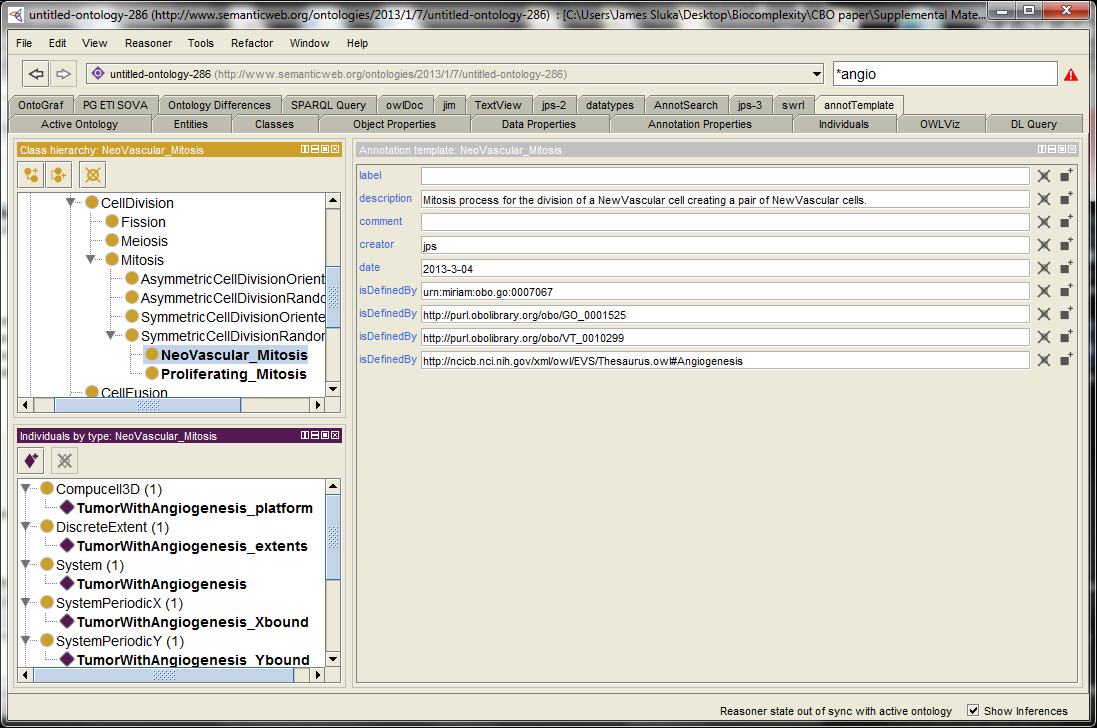


### Cell-Cell Interactions (Adhesions)

The CC3D script contains the definition of all possible pairwise adhesions between the prototype cell classes (including the extracellular matrix "Medium");

<Plugin Name="Contact">

<Energy Type1="Medium" Type2="Medium">0</Energy>

<Energy Type1="Proliferating" Type2="Medium">10</Energy>

<Energy Type1="Proliferating" Type2="Proliferating">8</Energy>

<Energy Type1="Necrotic" Type2="Medium">15</Energy>

<Energy Type1="Necrotic" Type2="Proliferating">8</Energy>

<Energy Type1="Necrotic" Type2="Necrotic">3</Energy>

<Energy Type1="Vascular" Type2="Medium">12</Energy>

<Energy Type1="Vascular" Type2="Proliferating">30</Energy>

<Energy Type1="Vascular" Type2="Necrotic">30</Energy>

<Energy Type1="Vascular" Type2="Vascular">5</Energy>

<Energy Type1="NeoVascular" Type2="Medium">12</Energy>

<Energy Type1="NeoVascular" Type2="Proliferating">30</Energy>

<Energy Type1="NeoVascular" Type2="Necrotic">30</Energy>

<Energy Type1="NeoVascular" Type2="Vascular">5</Energy>

<Energy Type1="NeoVascular" Type2="NeoVascular">5</Energy>

<NeighborOrder>4</NeighborOrder>

</Plugin>

We will create classes for each of these cell-cell adhesions described in the CC3D script. There are five cell types and 15 distinct pairwise combinations. It is helpful, but not required, to name these new model-specific classes in some consistent fashion. We will use the first letter or two of each of the two cell types and the string "Adh". So the CBO class for the adhesion of "Medium" with "Medium" will be called "M_M_Adh, for "Necrotic" to "NeoVascular" we will use N_NV_Adh. As in the case of creating cell prototypes the adhesion prototypes are all similar. We will take advantage of that by creating an initial, well annotated adhesion class then copying and editing that class to create the 15 total adhesion classes.

Our prototype will be NV_P_Adh for the adhesion of "NeoVascular" cells to "Proliferating" cells. Switch to a class view tab, click on CellCellAdhesion (or search for "CellCell" in the search box), and then the add subclass button (
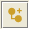
). Name the new class NV_P_Adh. Switch to the Annotation Template tab and annotate this class;


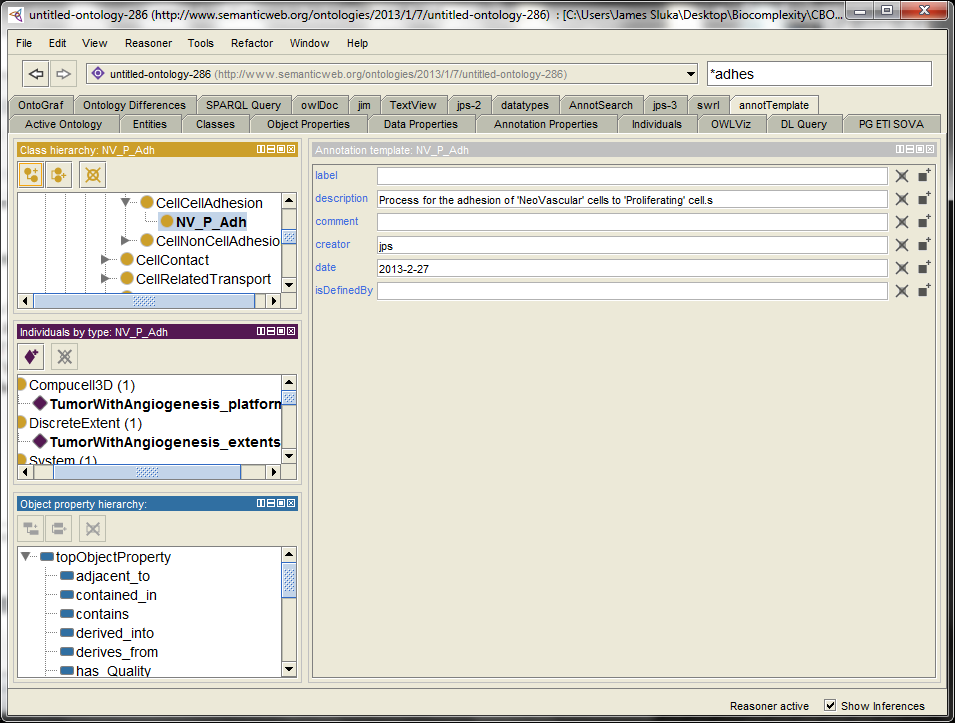


Since this class is a subclass of CellCellAdhesion it inherits the GO and PATO annotations (isDefinedBy) of CellCellAdhesion.

We now need to specify the participants in this process. Switch back to a class view and click on the "SubClass of +" in the Description frame to add a class restriction. In the popup window select the "Class expression editor" tab. In the box type (without the quotes) "has_p" then hit <tab> and select "has_participant" from the option list. Type a space then another <tab> and select "some" from the option list. Type another space then "p" then <tab> and select "Proliferating" from the list. Recall that "Proliferating" is the name of one of our prototype cell classes that we created earlier.


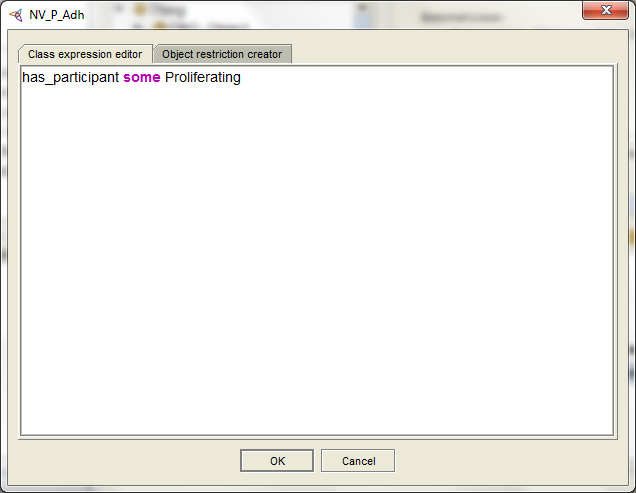


Click OK to close the dialog.

To add the other cell participant follow the same process to add another SubClass;


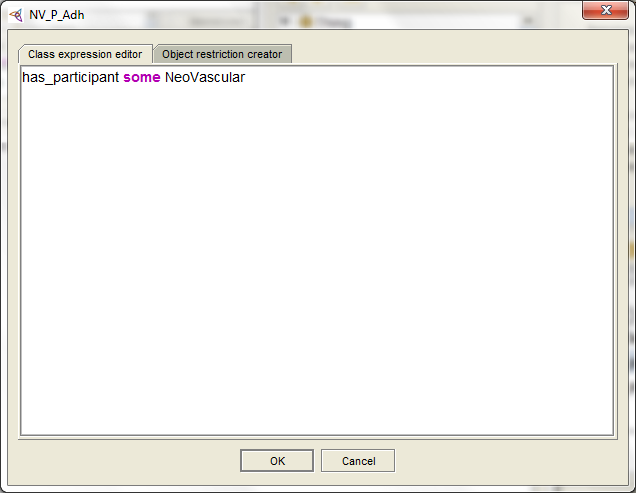


After synchronizing the reasoner the Class and Manchester syntax panels for "NV_P_Adh" looks like:


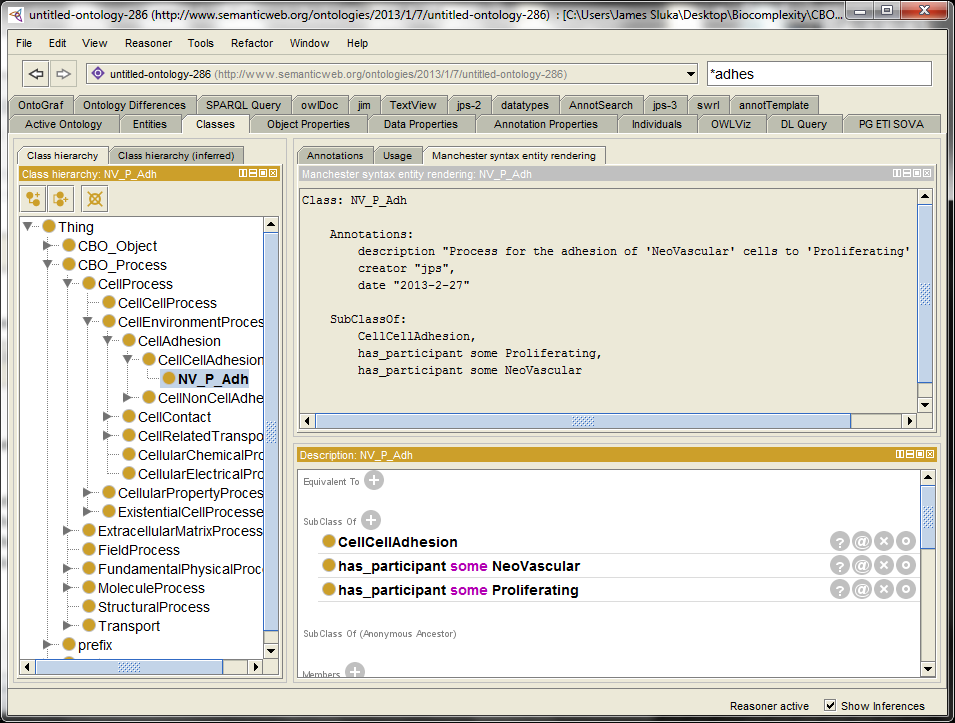


The participants in this adhesion process can also be described by a single "SubClass Of" restriction and, as before, the "some" qualifier might best be replaced by "exactly 1" if the reasoner used will allow it. For example, the pair of has_participant SubClass Of relations could be replaced by:

(has_participant exactly 1 NeoVascular)

and

(has_participant exactly 1 Proliferating)

Now that we have a properly defined and well annotated prototype class for the cell-cell adhesion process we can copy that NV_P_Adh class to create the other 14 adhesions in the model. In a class hierarchy view select NV_P_Adh and then use either the menus [Edit] – [Duplicate Selected Class…] or <Ctrl><Shift>C to duplicate the class. Protégé will require a new name for the new class. Remember to change both the annotations for each of the new adhesion classes and the "has_participant" class restrictions. When editing the SubClass you can click on the edit icon (
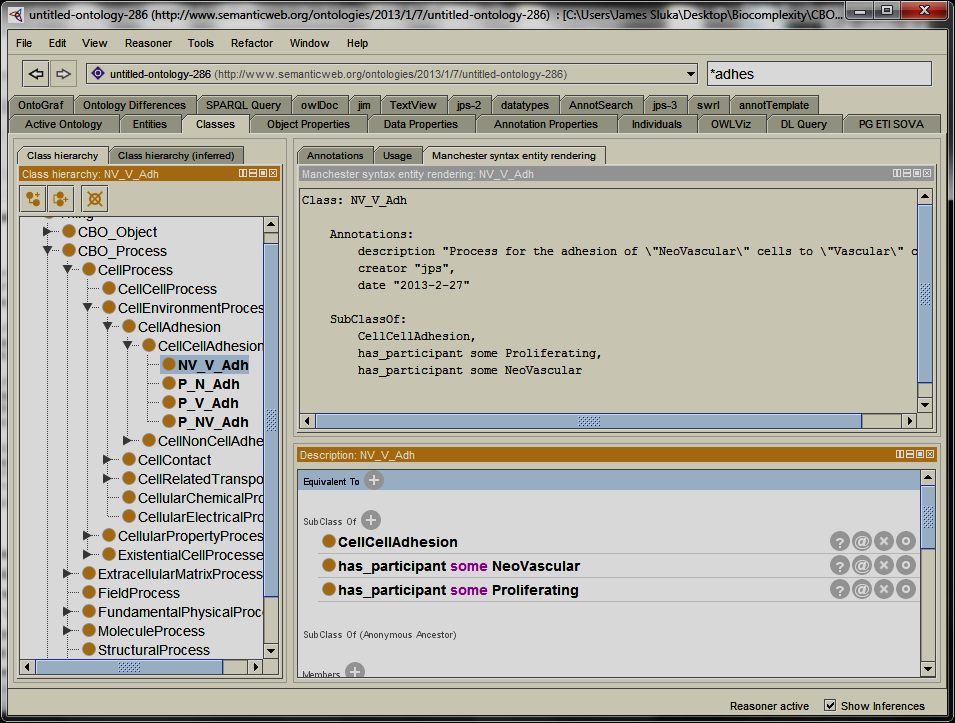
) for a particular restriction.

After creating the remaining 14 adhesions and synchronizing the reasoner:


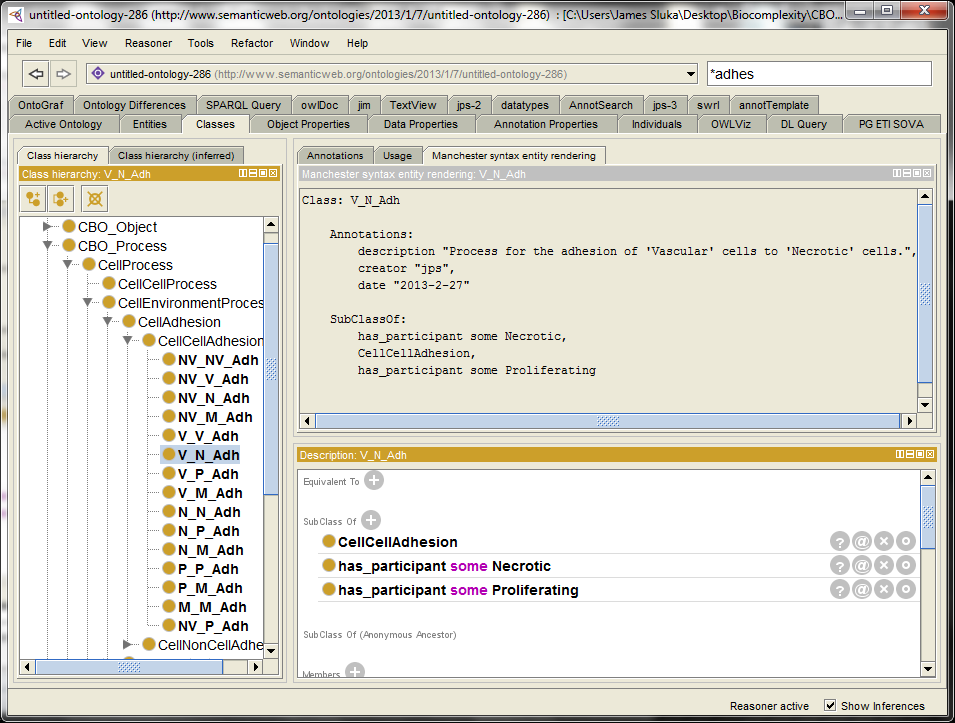


NOTE: This would be a good place for a macro that automatically generates all the pairwise combinations between the prototype cell classes!

We have another correction to make to our set of 15 adhesion processes. The cell type "Medium" actually represents the interstitial matrix and there are specific CBO adhesion classes for cells adhering to non-cells, in this case we want the process "CellInterstitialMatrixAdhesion". So for each of the adhesion processes with an "M" in the name we need to change the super-class from CellCellAdhesion to CellInterstitialMatrixAdhesion. A simple way to do this is to navigate to a class hierarchy view and then just drag-and-drop the class from its old position in the hierarchy to the new position.


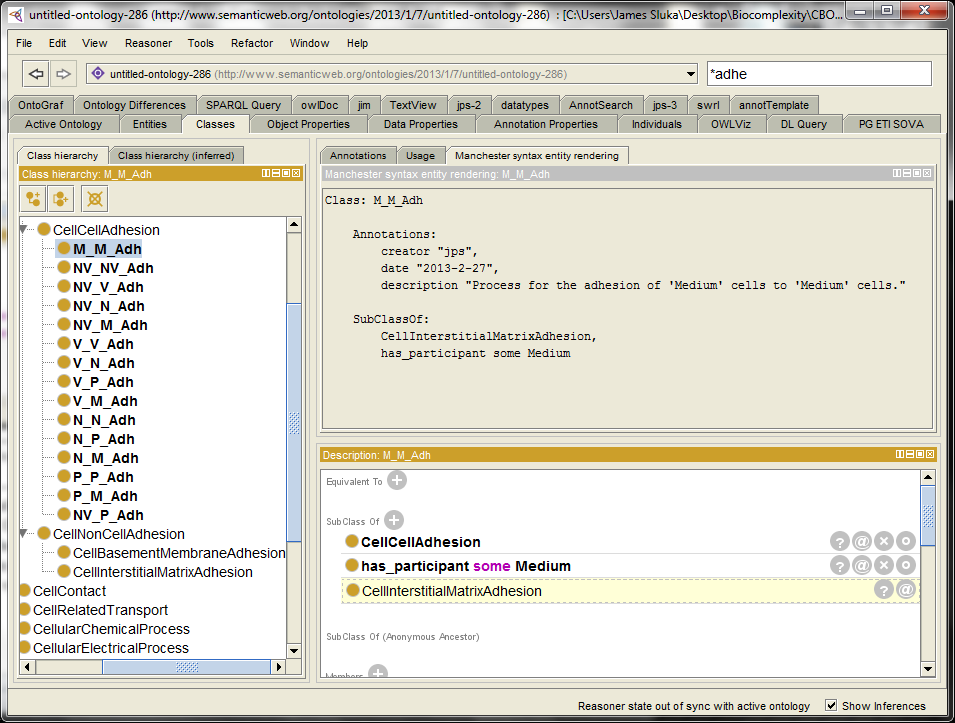

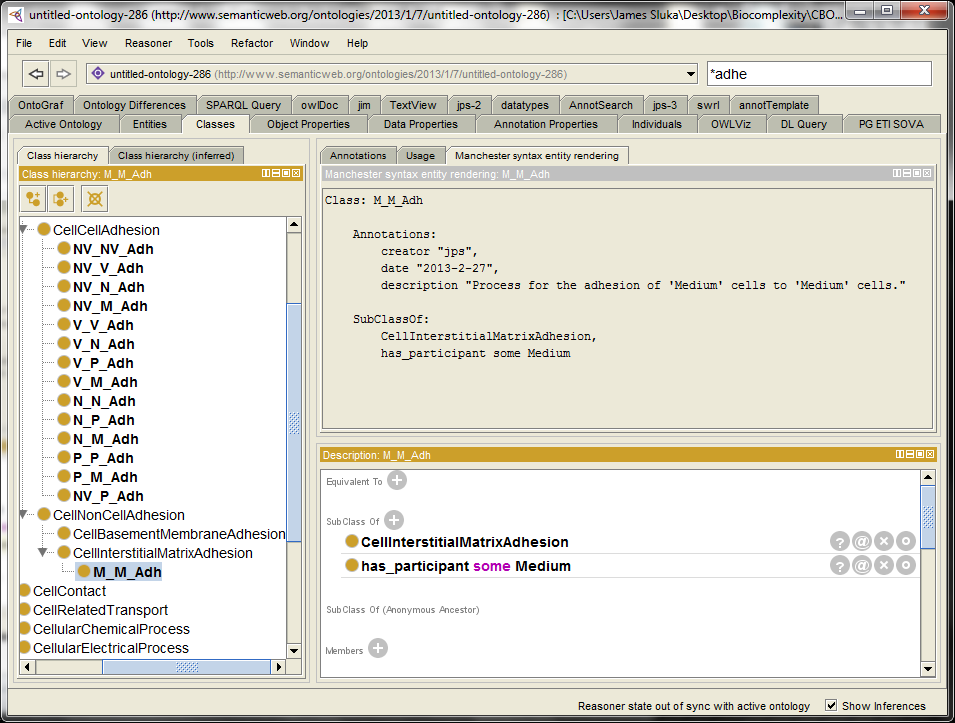


Drag

Hint: Dragging-and-dropping is a convenient way to move classes form one place to another in the hierarchy but it is also extremely easy to accidently move a class when you didn't intend to. If your mouse is moving when a class is selected in the hierarchy view it is easy to accidently, and often unnoticed, move the selected class to a new location in the hierarchy.

After moving all five of the adhesion classes that include Medium our hierarchy is now (recall that classes specific to the model-specific OWL ontology are shown in bold face, classes from the underlying CBO are in plain face):


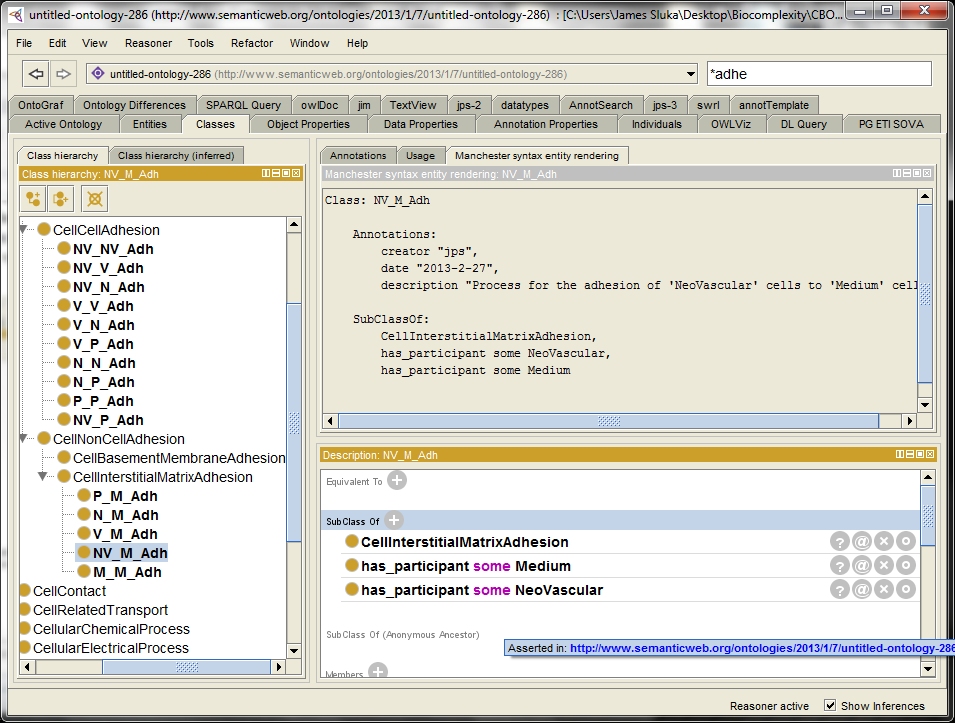


At present there is no CBO class specifically for adhesion between two portions of Medium so we leave M_M_Adh as a subclass of CellInterstitialMatrixAdhesion.

In a future release of CBO the classes need to specify the interaction energy will be added.

### Field Creation

In CC3D molecular diffusible fields are defined as shown below. Here we will include the characteristics marked with a **•** in the CBO description, the field name and the names of the source cell types and the names of the cell types in which the field decays (is destroyed).

<Steppable Type="FlexibleDiffusionSolverFE">

<!--Serialize Frequency="10"/-->

<!-- endothelial-derived short diffusing VEGF isoform-->

<DiffusionField>

<DiffusionData>

<FieldName>VEGF1</FieldName> **•**

<DiffusionConstant>0.010</DiffusionConstant>

<DecayConstant>0.0016</DecayConstant>

<DoNotDecayIn>Vascular </DoNotDecayIn>

<DoNotDecayIn>NeoVascular</DoNotDecayIn>

</DiffusionData>

<SecretionData>

<Secretion Type="NeoVascular">0.001</Secretion> **•**

<Secretion Type="Vascular" >0.001</Secretion> **•**

</SecretionData>

</DiffusionField>

...

</Steppable>

In particular, we will include the name of the field along with suitable external annotations for the molecule, the cell types that create the field and the cell types in which the field decays.

A molecular field is a CBO_Object:BioEntityType:Molecule , it's biological type, and CBO_Object:­PhysicalEntityType:­DiffuseEntity and CBO:Field:MolecularField, it's model representation type. (This is similar to the way cells are defined as both a biological type "Cell" and a model type "CorpuscularEntity".)

Should an individual field be a subclass or an individual? Because of the CBO representational similarities between Cells and Fields they should be done the same way. A model may have multiple fields but a particular field is probably best considered as existing at all times during the model, even if the concentration drops to zero everywhere. Therefore **Fields are subclasses (not individuals)**

A particular chemical field is a subclass of both CBO:Molecule and CBO:MolecularField. We will name the class using the name of the molecule. Navigate to the Classes tab and create a class named "VEGF1" as a subclass of CBO:MolecularField. In the description panel for this class click on "SubClass of +" and use the class hierarchy tab on the pop-up window to add subclass relations to CBO_Object:BioEntityType:Molecule and CBO_Object:PhysicalEntityType:DiffuseEntity.

When done the class panel for our model-specific VEGF1 molecular field should look like:


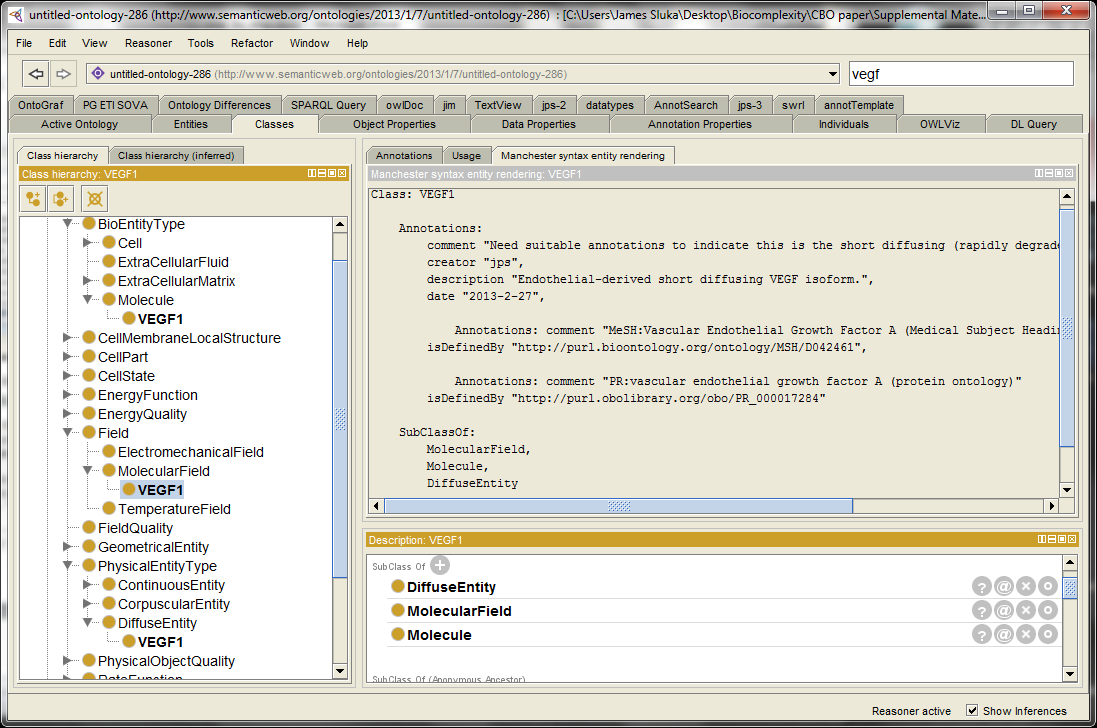


Note that the individual VEGF1 is listed under DiffuseEntity, MolecularField and Molecule classes. The Manchester syntax, including the added annotations:

Class: VEGF1

Annotations:

comment "Need suitable annotations to indicate this is the short diffusing (rapidly

degraded in the interstitial space) isoform.",

creator "jps",

description "Endothelial-derived short diffusing VEGF isoform.",

date "2013-2-27",

Annotations: comment "MeSH:Vascular Endothelial Growth Factor A (Medical Subject

Headings)"

isDefinedBy "http://purl.bioontology.org/ontology/MSH/D042461",

Annotations: comment "PR:vascular endothelial growth factor A (protein ontology)"

isDefinedBy "http://purl.obolibrary.org/obo/PR_000017284"

SubClassOf:

MolecularField,

Molecule,

DiffuseEntity

In the above some of the annotations have annoations. For example;
 Annotations: comment "PR:vascular endothelial growth factor A (protein ontology)"
is an annotation of the relation;
 isDefinedBy "http://purl.obolibrary.org/obo/PR_000017284"!

We now define the processes that create and destroy the VEGF1 field including the source cell types. Navigate to the class MoleculeProcess:MoleculeCreation and create a subclass called "VEGF1_creation" and then annotate the process using the Annotation Template tab. When done, the class view is:


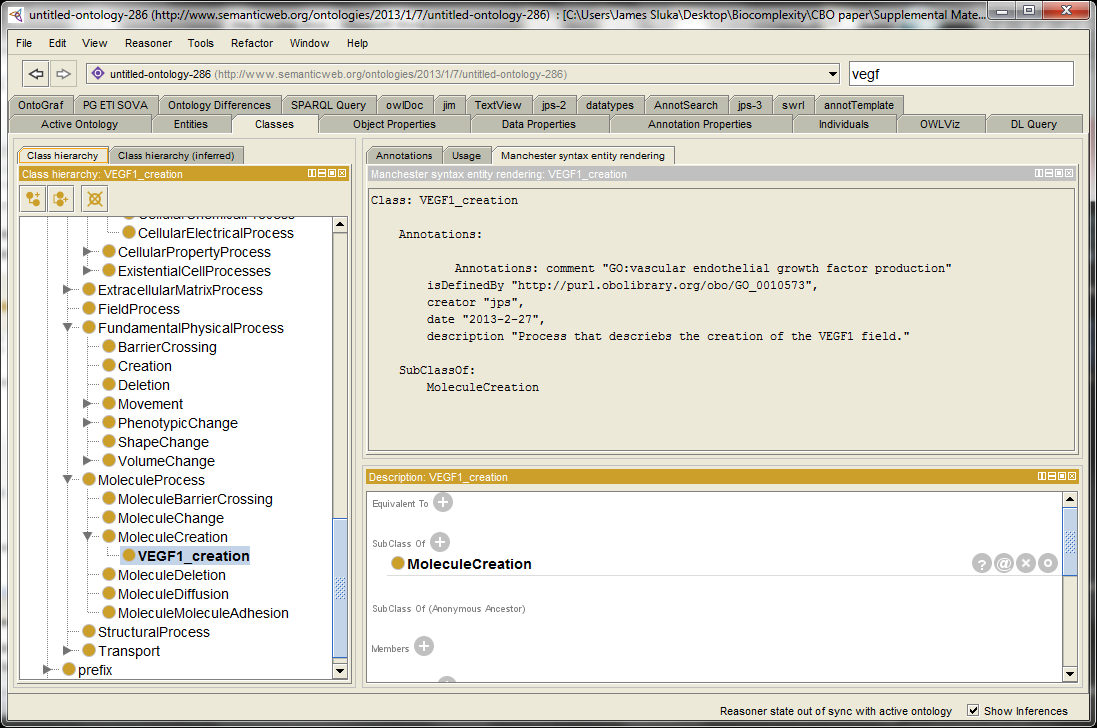


Simlarly create a VEGF1_deletion process class using the class copying procedure (<ctrl-shift>-C). Move the deletion process from a subclass of MoleculeCreation to bing a subclass of MoleculeDeleteion and correct the annotations.


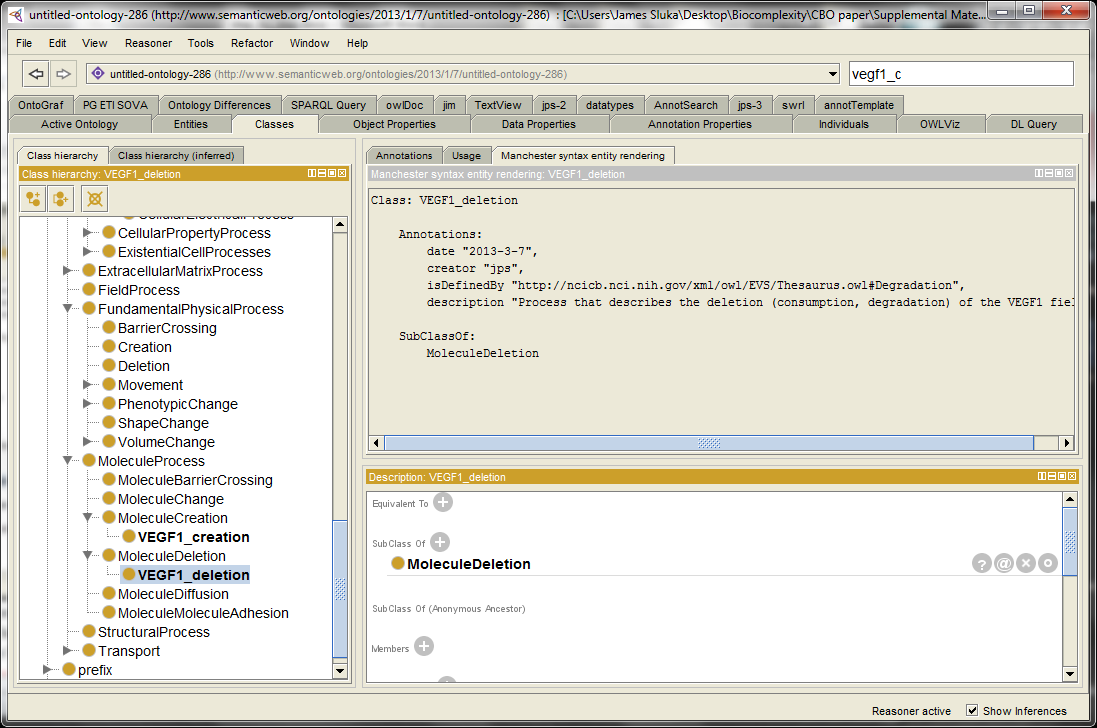
 We now link the creation and deletion process classes to our field object class "VEGF1". In a class panel navigate to the VEGF1 class then click on "SubClass of +" in the Description window, select the Class Expression Editor tab and enter "derives_from some VEGF1_creation ". The deletion process is defined simlarly with "derives_from some VEGF1_deletion".

Now that we have a class for the VEGF1 field and creation and deletion processes for that field we need to link the cell types that participate in the creation and deletion processes. Navigate to the Class tab and then to the VEGF1_creation class. In the Description window click the + for SubClasss Of and add "has_participant some (NeoVascular or Vascular)", which states that the VEGF1 field is created by cells of types NewVascular or Vascular.

The decay of the VEGF1 field is assumed to occur everywhere (in all cell types and in Medium) except the two cell types listed in the CC3D script, namely the same two cells types that create the field. To specify this select the VEGF1_deletion class and add the SubClass Of restriction "has_participant some (not NeoVascular and not Vascular)".

After synchronizing the reasoner the Manchester syntax for the VEGF1 field and the associated processes is (some annotations removed to simplyfy the listing):

**Class: VEGF1**

Annotations:

dc:description "Endothelial-derived short diffusing VEGF isoform.",

rdfs:isDefinedBy "http://purl.bioontology.org/ontology/MSH/D042461",

rdfs:isDefinedBy "http://purl.obolibrary.org/obo/PR_000017284"

SubClassOf:

MolecularField,

Molecule,

DiffuseEntity,

derives_from some VEGF1_creation,

derives_from some VEGF1_deletion

**Class: VEGF1_creation**

Annotations:

dc:description "Process that describes the creation of the VEGF1 field.",

rdfs:isDefinedBy "http://purl.obolibrary.org/obo/GO_0010573",

SubClassOf:

MoleculeCreation,

has_participant some (NeoVascular or Vascular)

**Class: VEGF1_deletion**

Annotations:

rdfs:isDefinedBy "http://ncicb.nci.nih.gov/xml/owl/EVS/Thesaurus.owl#Degradation",

dc:description "Process that describes the deletion (consumption, degradation) of the

VEGF1 field."

SubClassOf:

MoleculeDeletion,

has_participant some ((not (NeoVascular))and (not (Vascular)))

In addition to the VEGF1 field this model also has a VEGF2 (tumor-derived long diffusing VEGF isoform) and Glucose (representative limiting nutrient) fields. The VEGF2 field is created by the Proliferating cells and consumed by the Vascular and NeoVascular cells types. The Glucose field is created by the NeoVascular and Vascular cell types and consumed by the Proliferating cell type. The CC3D description of these two fields is:

<DiffusionField>

<DiffusionData>

<FieldName>VEGF2</FieldName>

<ConcentrationFileName></ConcentrationFileName>

<DiffusionConstant>0.15</DiffusionConstant>

<DecayConstant>0.0016</DecayConstant>

</DiffusionData>

<SecretionData>

<Secretion Type="Proliferating">0.001</Secretion>

<Uptake Type="NeoVascular" MaxUptake="0.05" RelativeUptakeRate="0.5"/>

<Uptake Type="Vascular" MaxUptake="0.05" RelativeUptakeRate="0.5"/>

</SecretionData>

</DiffusionField>

<DiffusionField>

<DiffusionData>

<FieldName>Glucose</FieldName>

<ConcentrationFileName>Simulation/Glucose_300.dat</ConcentrationFileName>

<DiffusionConstant>0.15</DiffusionConstant>

</DiffusionData>

<SecretionData>

<Secretion Type="NeoVascular">0.4</Secretion>

<Secretion Type="Vascular" >0.8</Secretion>

<Uptake Type="Medium" MaxUptake="0.0064" RelativeUptakeRate="0.1"/>

<Uptake Type="Proliferating" MaxUptake="0.1" RelativeUptakeRate="0.1"/>

</SecretionData>

</DiffusionField>

We can use the VEGF1 field (object) class and process classes VEGF1_creation and VEGF1_deletion classes, as prototypes for the two new fields (VEGF2 and Glucose) and four new processes (VEGF2_creation, VEGF2_deletion, Glucose_creation and Glucose_deletion). Once the new classes are created we need to edit the annotations and the SubClass Of relations. The Glucose field should be annotated via ChEBI (CHEBI_17234). Once the new classes have been created from the VEGF1 classes and edited the set of nine classes for the three diffusible fields in Manchester syntax (some annotations omitted):

**Class: VEGF1**

Annotations:

dc:description "Endothelial-derived short diffusing VEGF isoform.",

rdfs:isDefinedBy "http://purl.bioontology.org/ontology/MSH/D042461",

rdfs:isDefinedBy "http://purl.obolibrary.org/obo/PR_000017284"

SubClassOf:

MolecularField,

Molecule,

derives_from some VEGF1_creation,

derives_from some VEGF1_deletion,

DiffuseEntity

**Class: VEGF1_creation**

Annotations:

dc:description "Process that describes the creation of the VEGF1 field.",

rdfs:isDefinedBy "http://purl.obolibrary.org/obo/GO_0010573",

SubClassOf:

MoleculeCreation,

has_participant some (NeoVascular or Vascular)

**Class: VEGF1_deletion**

Annotations:

rdfs:isDefinedBy "http://purl.obolibrary.org/obo/GO_0010573",

dc:description "Process that describes the deletion (consumption, degradation, clearance)
 of the VEGF1 field."

SubClassOf:

has_participant some ( (not (NeoVascular)) and (not (Vascular)) ),

MoleculeDeletion

**Class: VEGF2**

Annotations:

dc:description "tumor-derived long diffusing VEGF isoform",

rdfs:isDefinedBy "http://purl.bioontology.org/ontology/MSH/D042461",

rdfs:isDefinedBy "http://purl.obolibrary.org/obo/PR_000017284"

SubClassOf:

derives_from some VEGF2_creation,

derives_from some VEGF2_deletion,

MolecularField,

Molecule,

DiffuseEntity

**Class: VEGF2_creation**

Annotations:

dc:description "Process that describes the creation of the VEGF2 field.",

rdfs:isDefinedBy "http://purl.obolibrary.org/obo/GO_0010573",

SubClassOf:

MoleculeCreation,

has_participant some Proliferating

**Class: VEGF2_deletion**

Annotations:

dc:description "Process that describes the deletion (consumption, degradation, clearance)
 of the VEGF2 field."

SubClassOf:

has_participant some (NeoVascular or Vascular),

MoleculeDeletion

**Class: Glucose**

Annotations:

dc:description "Glucose (as a representitive limiting nutrient)",

rdfs:isDefinedBy "http://purl.obolibrary.org/obo/CHEBI_17234",

SubClassOf:

MolecularField,

Molecule,

derives_from some Glucose_creation,

derives_from some Glucose_deletion,

DiffuseEntity

**Class: Glucose_creation**

Annotations:

dc:description "Process that describes the creation of the Glucose field.",

SubClassOf:

MoleculeCreation,

has_participant some (NeoVascular or Vascular)

**Class: Glucose_deletion**

Annotations:

rdfs:isDefinedBy "http://purl.obolibrary.org/obo/GO_0010573",

dc:description "Process that describes the deletion (consumption, degradation, clearance)
 of the Gucose field."

SubClassOf:

has_participant some (Medium or Proliferating),

MoleculeDeletion

### Chemotaxis

The tumor growth with angiogenesis model includes several chemotaxis processes. Chemotaxis requires specifying the cells types that move and the field(s) (usually chemical) that direct the chemotaxis. In the CC3D script chemotaxis processes are defined by:

<Plugin Name="Chemotaxis">

<ChemicalField Source="FlexibleDiffusionSolverFE" Name="VEGF1">

<ChemotaxisByType Type="NeoVascular" Lambda="8000"

ChemotactTowards="Medium,Proliferating,Necrotic" />

</ChemicalField>

<ChemicalField Source="FlexibleDiffusionSolverFE" Name="VEGF2">

<ChemotaxisByType Type="NeoVascular" Lambda="1000"

ChemotactTowards="Medium,Proliferating,Necrotic" SaturationCoef="0.05"/>

</ChemicalField>

<ChemicalField Source="FlexibleDiffusionSolverFE" Name="VEGF1">

<ChemotaxisByType Type="Vascular" Lambda="8000"

ChemotactTowards="Medium,Proliferating,Necrotic" />

</ChemicalField>

</Plugin>

The above CC3D code snippet specifies that cell types NeoVascular and Vascular chemotax to the VEGF1 field but only NeoVascular cells chemotax to the VEGF2 field. Furthermore, for all three chemotaxis specifications the moving cell can only displace (push) cells of types Medium, Proliferating and Necrotic. (This last restriction cannot currently be specified in CBO). In CBO chemotaxis is specified with the class CBO_Process:…:Chemotaxis. We will name our chemotaxis processes based on the molecule field names; "VEGF1_chemotaxis" and "VEGF2_chemotaxis". On the class view tab enter "chemo" in the search box then select Chemotaxis from the dropdown. In the Class Hierarchy make sure Chemotaxis is selected then click the Add Subclass button (
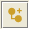
). Name the new class VEGF1_chemotaxis. Switch to the Annotation Template tab and annotate this process. Switch back to the Classes tab and in the Description window add the SubClass Of definitions "has_participant some (NeoVascular or Vascular)" and "has_participant some VEGF1". The Classes tab for the VEGF1_chemotaxis class should now look like:


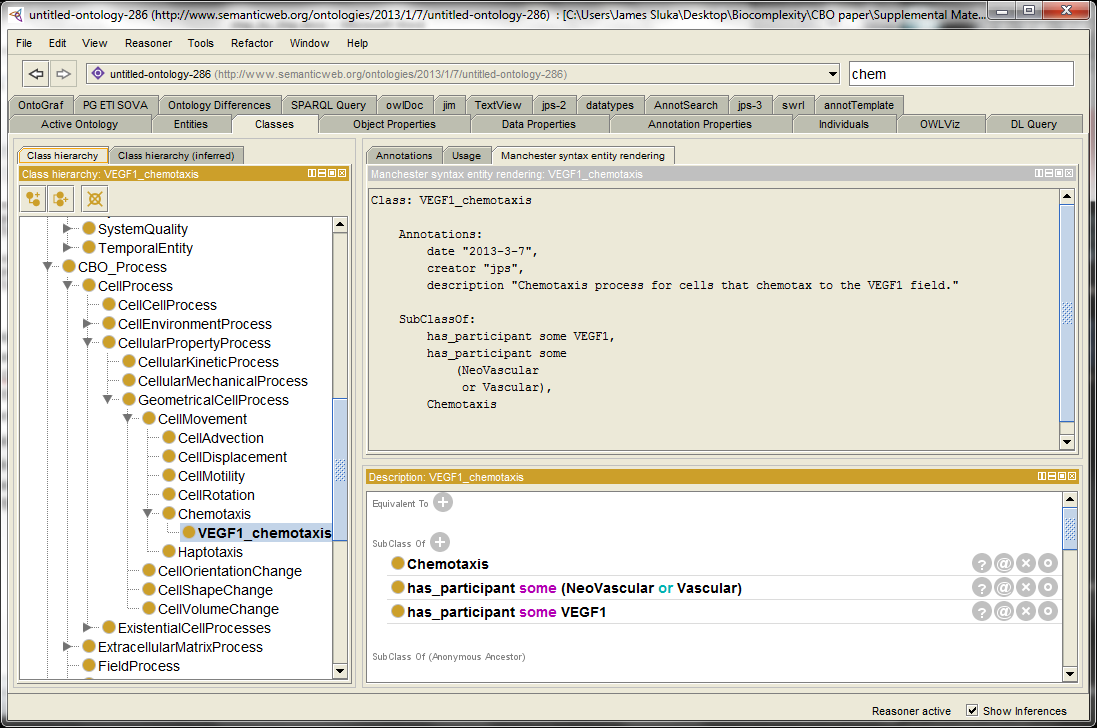


The pair of SubClass Of relations shown above can be collapsed into a single restriction:
 has_participant only ( (NeoVascular or Vascular) and VEGF1 )

To create the VEGF2_chemotaxis process class we can just copy the VEGF1_chemotaxis class and edit the annotations and SubClass Of properties (recall that only the NeoVascular cells chemotax to the VEGF2 field):


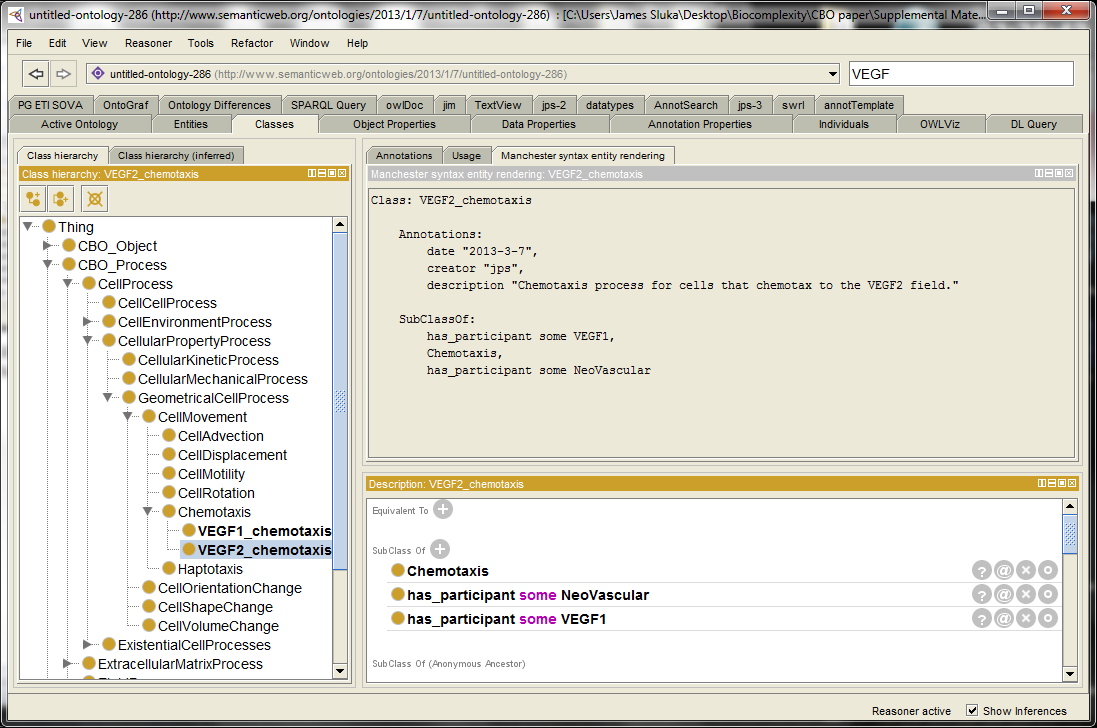


The Manchester rendering for our two chemotaxis process is (most annotations removed):

**Class: VEGF1_chemotaxis**

Annotations:

dc:description "Chemotaxis process for cells that chemotax to the VEGF1 field."

SubClassOf:

Chemotaxis,

has_participant some VEGF1,

has_participant some (NeoVascular or Vascular)

**Class: VEGF2_chemotaxis**

Annotations:

dc:description "Chemotaxis process for cells that chemotax to the VEGF2 field."

SubClassOf:

Chemotaxis,

has_participant some VEGF1,

has_participant some NeoVascular

## Conclusion

We have completed our description of the tumor growth with angiogenesis multicell model, using the CBO as our underlying ontology, in Protégé 4. The resulting OWL file, AngioTumor.owl, is a new ontology, based on the CBO, specific to this particular multicell model. Our new ontology describes the objects, qualities and processes in the model but does not describe the results of actually running the model. Instead our OWL ontology, in conjunction with the VTK files created during the running of the model, create a data structure from which the results of running the model can be extracted. In the next section we will give a simple demonstration of using the combination of the OWL model-specific ontology along with the simulation produced VTK files to extract information about the results of the simulation.

# Sample Usage of VTK files for Lattice and Center Based Models

The Visualization Toolkit (VTK <http://www.vtk.org/>) is an open-source software system for 3D computer graphics, image processing and visualization. Here we are primarily concerned with using VTK files to store simulation results from multicell computational models. The VTK format, though designed for graphics, has several important characteristics that separate it from most other graphics formats. Three particularly important aspects of the VTK format(s) are (1) the ability to store an arbitrary number of channels (as opposed to just RGB or CMYK), (2) the ability to store signed floating point values (as opposed to most graphic descriptions using unsigned integers) and (3) the ability to represent 3D systems. In the VTK nomenclature a "channel" is called a "field". VTK supports several different native formats described at <http://www.vtk.org/VTK/img/file-formats.pdf>. We will focus on the VTK legacy formats "STRUCTURED_POINTS" and " POLYDATA". STRUCTURED_POINTS style is used to describe regular lattices and POLYDATA for continuous space. For simplicity we will use the ASCII version of the VTK files though they can also be stored in binary format.

## Example 1: Lattice model with cell types and a field.

Our first example consists of a simple 5x5 2D lattice containing two cells with cell types (CellType) of "4" and "12" along with medium of cell type "0". Medium fills all the lattice sites not occupied by the two cells. The individual cells are defined by cell IDs (CellId) of "1" and "2". Note that CellType and CellId are distinct concepts and the same numeric value can occur in both contexts though they would have different meanings. In addition, there is a field, named "FieldA", that overlays the cells and medium and is described by a floating point number for each lattice site. The table below list the three VTK fields.

| Cell ID Field "CellId" | Cell Type Field "CellType" | Field "FieldA" |
| --- | --- | --- |
| \| 10 \| 11 \| 12 \| 13 \| 14 \| \| --- \| --- \| --- \| --- \| --- \| \| 15 \| 1 \| 1 \| 2 \| 2 \| \| 16 \| 1 \| 1 \| 2 \| 27 \| \| 17 \| 18 \| 19 \| 20 \| 21 \| \| 22 \| 23 \| 24 \| 25 \| 26 \|   *NOTE:* <0,0> is bottom left | \| 0 \| 0 \| 0 \| 0 \| 0 \| \| --- \| --- \| --- \| --- \| --- \| \| 0 \| 4 \| 4 \| 12 \| 12 \| \| 0 \| 4 \| 4 \| 12 \| 0 \| \| 0 \| 0 \| 0 \| 0 \| 0 \| \| 0 \| 0 \| 0 \| 0 \| 0 \| | \| 1 \| 1.3 \| 1.5 \| 1.5 \| 1.5 \| \| --- \| --- \| --- \| --- \| --- \| \| 1.1 \| 1.5 \| 1.5 \| 0.1 \| 0.1 \| \| 1.1 \| 1.5 \| 1.5 \| 0.1 \| 1 \| \| 1.2 \| 1.2 \| 1.3 \| 1.4 \| 1.5 \| \| 1.2 \| 1.2 \| 1.3 \| 1.4 \| 1.5 \| |

These three tables specify that, for example, the lattice site at <0,1> is part of the cell with CellId 17, which is of CellType 0 (a pixel of medium). In addition the value of FieldA at that lattice site is 1.2. The VTK file (STRUCUTRED_POINTS, ASCII data) for this example is:

# vtk DataFile Version 2.0

Example 1: Lattice model with cell types and a field

ASCII

DATASET STRUCTURED_POINTS

DIMENSIONS 5 5 1

SPACING 1 1 1

ORIGIN 0 0 0

POINT_DATA 25

FIELD FieldData 3

CellType 1 25 int

0 0 0 0 0

0 4 4 12 12

0 4 4 12 0

0 0 0 0 0

0 0 0 0 0

CellId 1 25 long

10 11 12 13 14

15 1 1 2 2

16 1 1 2 27

17 18 19 20 21

22 23 24 25 26

FieldA 1 25 double

1.0 1.3 1.5 1.5 1.5

1.1 1.5 1.5 0.1 0.1

1.1 1.5 1.5 0.1 1.0

1.2 1.2 1.3 1.4 1.5

1.2 1.2 1.3 1.4 1.5

As you can see the VTK file consists of a header block (first eight lines), a line describing how many fields are in the file (FIELD FieldData 3), then blocks to describe each of those fields (CellId, CellType and FieldA) giving the values for each lattice site.

If we rendered these three fields in a tool such as ParaView (http://www.paraview.org/) (note that <0,0> is top left corner in ParaView) we get graphical representations of the data in each field:
 CellId CellType FieldA


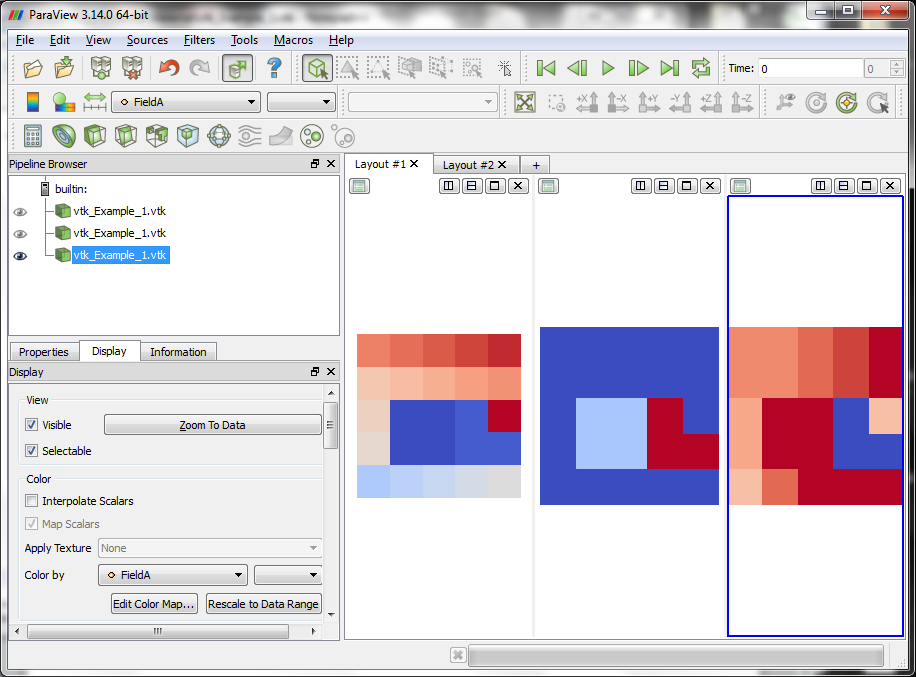


In the above example every pixel (voxel, lattice site) of type "0", representing Medium or extracellular space, is assigned its own CellId. In some multicell modeling packages, such as Compucel3D (CC3D www.compucell3d.org), "Medium" (CellType="0") is a special class of object and all pixels of Medium have the same CellId "0" and the same CellType "0". In images above the left most image, CellId, is the only one that would change and all of the CellType "0" pixels would be colored the same since they would all have CellId of "0".

An arbitrary number of fields can be included in a VTK file, though the CellId and CellType fields are required. Note that VTK specifically uses the word "cell", though not in exactly the same way as a biologists uses that term. A VTK "cell" is a set of points in 3D space that have the same ID, defined by CellId, and are of the same type, defined by the CellType, and are considered to make up a single object named by the CellId.

The VTK Fields can contain integers (signed and unsigned) or floating point numbers. In the VTK sample above CellId is defined as a "long"(integer) and FieldA as a "double". The example of FieldA given above demonstrates representing a pixel based quantity (such as the concentration of a diffusible molecule) in a VTK file. Values representing any numeric quantity can be included in a VTK file by adding a field of the appropriate numeric type. A field could also describe some quantity associated with an entire VTK cell (or the biological cell it represents). For example, in a biological model a field could be included giving the generational age of the "biological cells", or a field describing the state of some internal process of the biological cells (e.g., 0,1), or the volume of the cell (which could be calculate form the pixel list in the VTK file). In the case of these cell based quantities the VTK lattice description will be somewhat redundant since the field value for a cell based quantity is duplicated for every pixel of the cell.

This basic data structure can be expanded to three dimensions, indeed the VTK file shown earlier is actual 3D but the dimension on one of the axis is 1, as defined in the line "DIMENSIONS 5 5 1". Rendering 3D data sets presents challenges, in particular since "Medium" is often an actual cell type ("cell" in the VTK sense of the word, which is distinct form the computational or biological senses), so in general Medium would be either not rendered or rendered (semi-)transparently.

Time can be represented in VTK in a number of ways. It is possible to include multiple VTK "snap shots", each representing a particular instance in time, in a single VTK file. We prefer to create a new VTK file for each snap shot or time instance. A multicell simulation generates a series of VTK files and the time coordinate can be included as part of the VTK file name.

## Example 2: Center model with cell types, cell centers and radii.

The VTK format described above, "STRUCTURED_POINTS ", is for regular lattices. In addition VTK files can describe "center models". A center model describes cells (VTK, biological or computational) as a point in space for the center of a sphere and a radius for the sphere. For example, a center model with three cells (IDs:1,2,3), two cell types (1,3), cell radii and Center of Mass (COM) coordinates specified for each cell is shown below.

ID: 3
Type:1
COM=<5.5,4.2,0>
r=1

ID: 1
Type:1
COM=<1.1,3.4,0>r=1

ID: 2
Type:3
COM=<4,4,0>r=1.5

A VTK file for this model uses the "DATASET POLYDATA" file type and is shown below.

# vtk DataFile Version 2.0

Example 1: Lattice model with cell types and a field

ASCII

DATASET POLYDATA

POINTS 3 float

1.1 3.4 0.0

4.0 4.0 0.0

5.5 4.2 0.0

VERTICES 3 6

1 0

1 1

1 2

POINT_DATA 3

SCALARS cellType int

LOOKUP_TABLE default

1 2 1

SCALARS cellID int

LOOKUP_TABLE default

1 2 3

SCALARS cellRadius float

LOOKUP_TABLE default

1.0 1.5 1.0

Note that

VERTICES 3 6

1 0

Means there are 3 vertices, 6 data values and there is a vertex at cell "0" (Not that cells "1" and "0" are a vertex). So 3 of the six data values simply list the number of points on a particular line.

The VTK center model file above can be displayed in ParaView Display as circular (spherical) glyphs of Radius cellRadius:


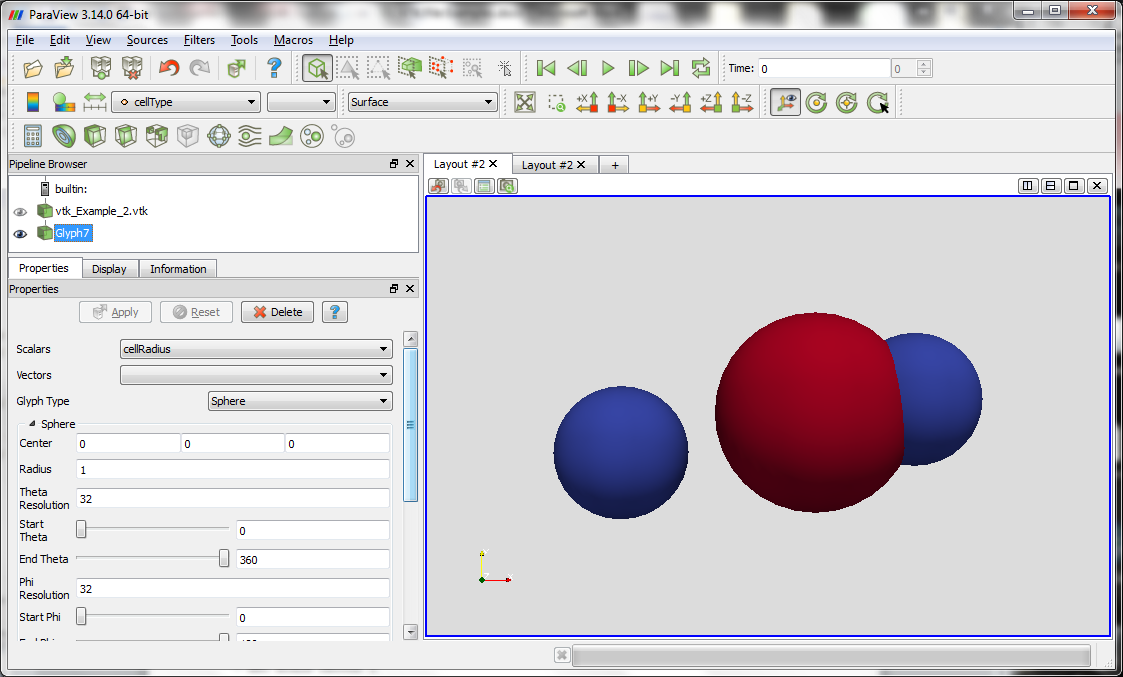


(NOTE: ParaView places the origin <0,0,0> in the upper left instead of the bottom left of the view window.)

The exact nature of the intersection between the two spheres in the image above was calculated by ParaView. Other rendering tools, as well as modeling tools, may treat the intersection differently than in the above image. In addition, in a center model representation some basic qualities of individual cells, for example a cell's volume, can only be determined if the cell of interest and the neighboring cells are specified. In the image above the two blue cells have different volumes even though they have the same radii.

## Example 3: Center model with cell types and fields.

The POLYDATA format used to described center models does not appear to be able to also described a pixel based quantity such as a diffusible molecular field. To present both a center model and a lattice-based field model requires both a POLYDATA and a STRUCTURED_POINTS description. The advanced VTK file formats can handle this case. Here we will stay with the simpler legacy formats and simply use two VTK file for the data. In the example below we combine the three cell center model (from Example 2) with a separate file describing the lattice for the diffusible field (similar to Example 1). In calculating the field it was assumed that Cell type 2 is opaque to the field and cell type 1 is transparent to the field.

| vtk_Example_3a.vtk (center model cells): |
| --- |
| # vtk DataFile Version 2.0  Example 3: Center model with cell types, COM and radii, Part A: cell defintions  ASCII  DATASET POLYDATA  POINTS 3 float  1.1 3.4 0.0  4.0 4.0 0.0  5.5 4.2 0.0  VERTICES 3 6  1 0  1 1  1 2  POINT_DATA 3  SCALARS cellType int  LOOKUP_TABLE default  1 2 1  SCALARS cellID int  LOOKUP_TABLE default  1 2 3  SCALARS cellRadius float  LOOKUP_TABLE default  1.0 1.5 1.0 |
| vtk_Example_3b.vtk (lattice model field): |
| # vtk DataFile Version 2.0  Example 3: Center model with cell types, COM and radii, Part B: Field definitions  ASCII  DATASET STRUCTURED_POINTS  DIMENSIONS 40 40 1  SPACING 0.2 0.2 0.2  ORIGIN 0 0 0  POINT_DATA 1600  FIELD FieldData 1  FieldA 1 1600 double  0 1 4 9 16 25 36 49 64 81 100 121 144 169 196 225 256 289 324 361 400 441 484 529 576 625 676 729 784 841 900 961 1024 1089 1156 1225 1296 1369 1444 1521  1 4 9 16 25 36 49 64 81 100 121 144 169 196 225 256 289 324 361 400 441 484 529 576 625 676 729 784 841 900 961 1024 1089 1156 1225 1296 1369 1444 1521 1600  4 9 16 25 36 49 64 81 100 121 144 169 196 225 256 289 324 361 400 441 484 529 576 625 676 729 784 841 900 961 1024 1089 1156 1225 1296 1369 1444 1521 1600 1681  . . . |

Rendering this pair of VTK files in ParaView generates the images below. The upper left image is the center model only, upper right the field model only. Notice the "hole" in the field at the type 2 cell, which is opaque to the field, compared to the even field across the "transparent" cells of type 1. Also notice the coarseness of the field lattice compared to the much smoother rendering of the spheres in the center model. The bottom left image overlays both models and the center model is rendered semi-transparent, the bottom right image renders the center model as opaque. In both of the bottom images the center model is stacked "on top" of the field model.


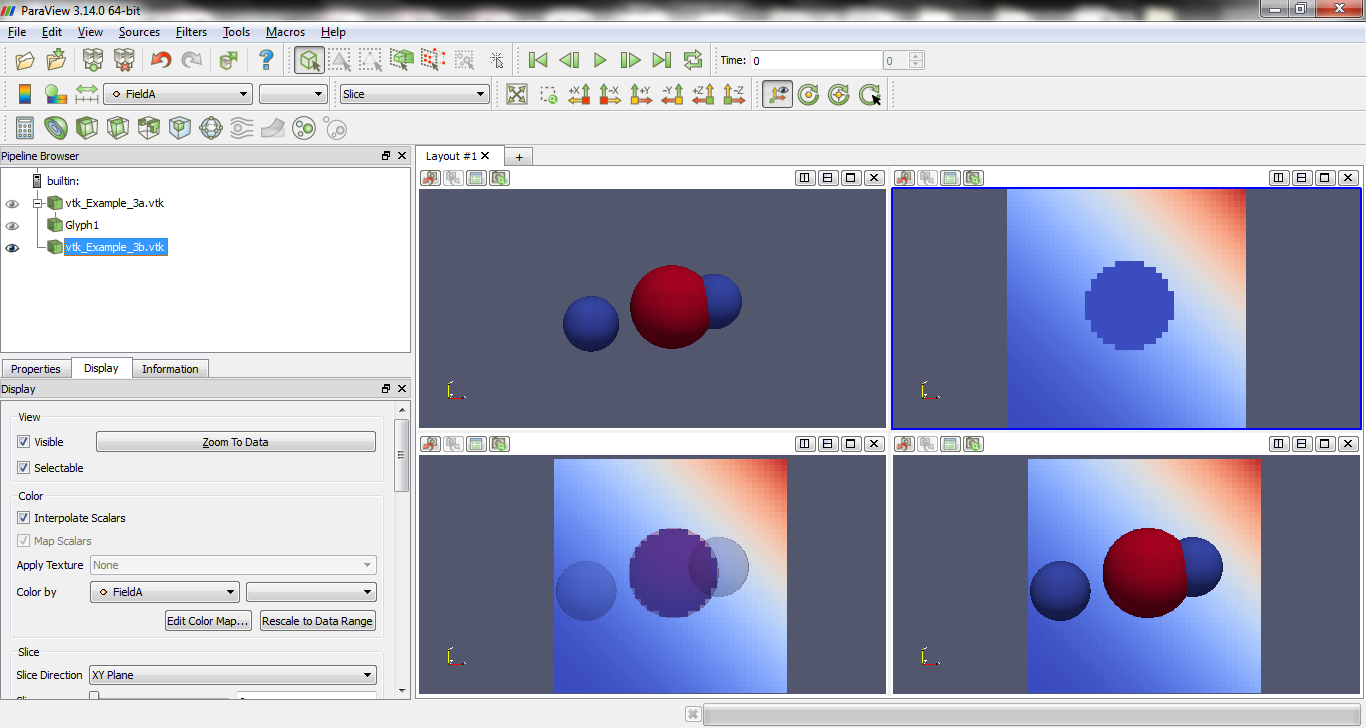


# Sample SPARQL Queries

Here we present a few examples of SPARQL (SPARQL Protocol and RDF Query Language, the acronym is recursive, hence the first "S") queries directed at both CBO and at the tumor growth with angiogenesis metamodel. SPARQL is the structured query language for OWL ontologies. SPARQL queries are similar in syntax to SQL (Structured Query Language) queries for relational databases.

Our first SPARQL query extracts all of the references to external ontologies that occur in the CBO. In Protégé 4 load the CBO and select the SPARQL Query" tab. (If the tab is not visible go to Protégé's <Window><tabs> and check the "SPARQL Query" item.)

SELECT ?s ?p ?o

WHERE {

?s ?p ?o .

FILTER(regex(?o,"subclass of: BFO","i")) .

}

ORDER BY ?s

Returns:


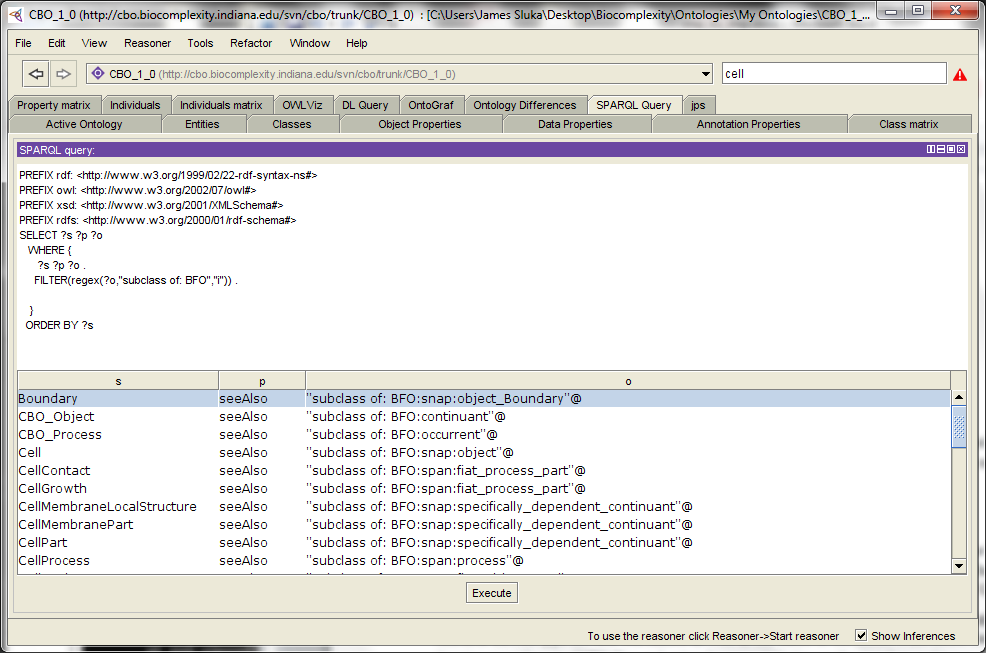


The BFO (Basic Formal Ontology) links are structured as "isA" (subClassOf) which cannot be directly represented in Protégé 4 without actually importing all of BFO.

The SPARQL query below returns the list of classes that are linked to external resources via "isDefinedBy" properties.

PREFIX rdfs: <http://www.w3.org/2000/01/rdf-schema#>

SELECT ?s ?o

WHERE {

?s rdfs:isDefinedBy ?o .

}

ORDER BY ?s

Returns:


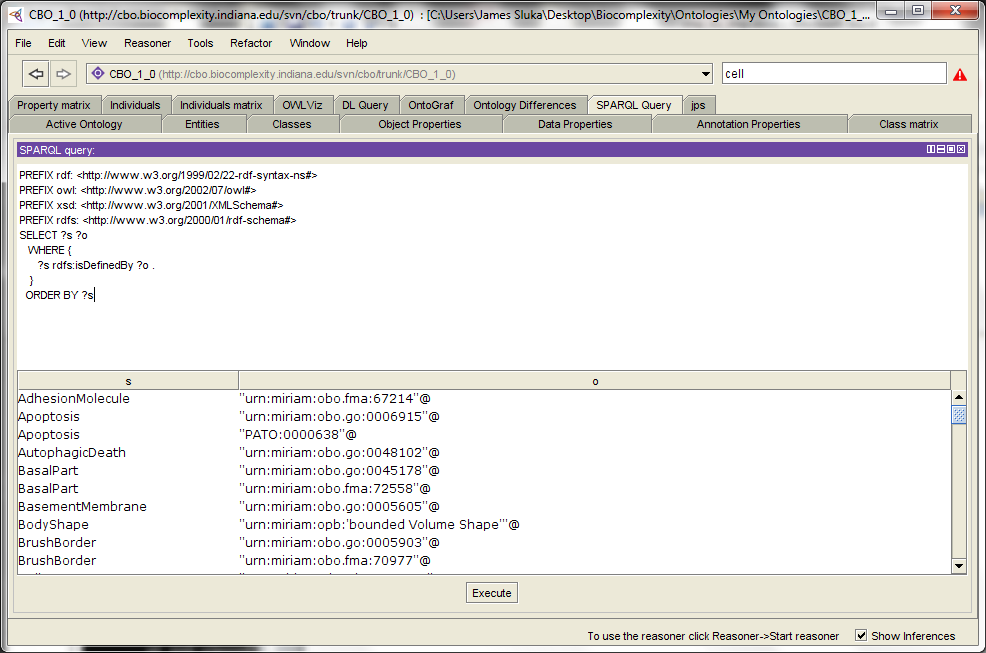


If we just want external links to, for example, GO we could use the SPARQL:

PREFIX rdfs: <http://www.w3.org/2000/01/rdf-schema#>

SELECT ?s ?o

WHERE {

?s rdfs:isDefinedBy ?o .

FILTER(regex(?o,"GO","i")) .

}

ORDER BY ?s

Returns:


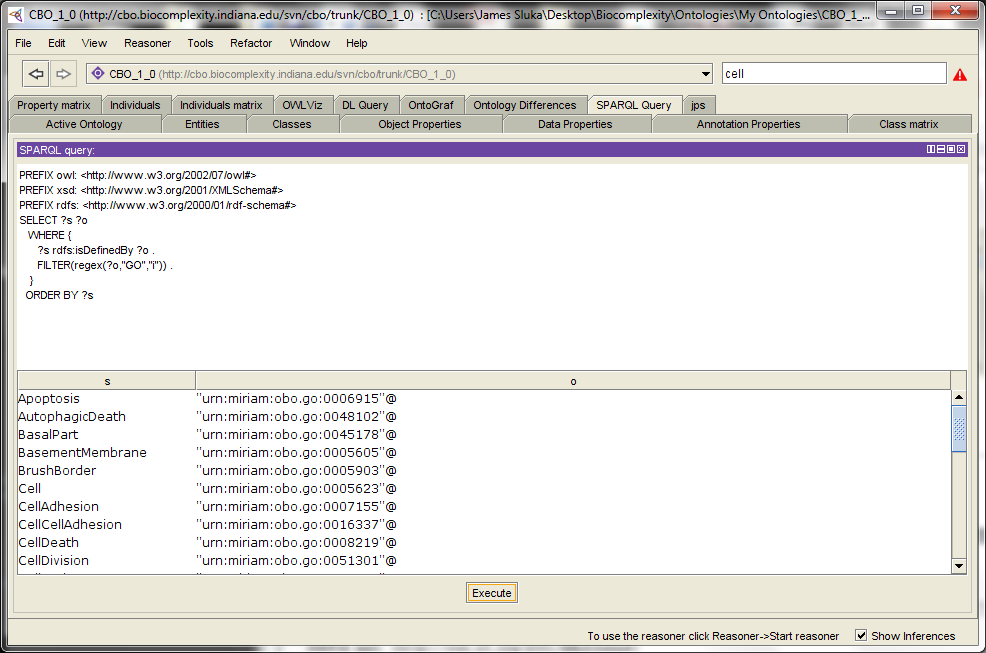


Be nice to have the ability to expand the GO terms to their "names" instead of their IDs, perhaps via BioPortal.

Typical queries for a metamodel file might include a SPARQL query to extract all of the "real" (biological) cells in the metamodel. Open the metamodel file in Protégé 4 and use the SPARQL query;

PREFIX owl: <http://www.w3.org/2002/07/owl#>

PREFIX rdfs: <http://www.w3.org/2000/01/rdf-schema#>

PREFIX CBO: <http://cbo.biocomplexity.indiana.edu/svn/cbo/trunk/CBO_1_0.owl#>

SELECT ?subject ?pred ?idVal

WHERE {

?subject rdfs:subClassOf CBO:Cell .

?subject ?pred ?idVal .

?subject owl:equivalentClass ?idVal . }

Returns the prototype cells defined in the metamodel along with their VTK ids:


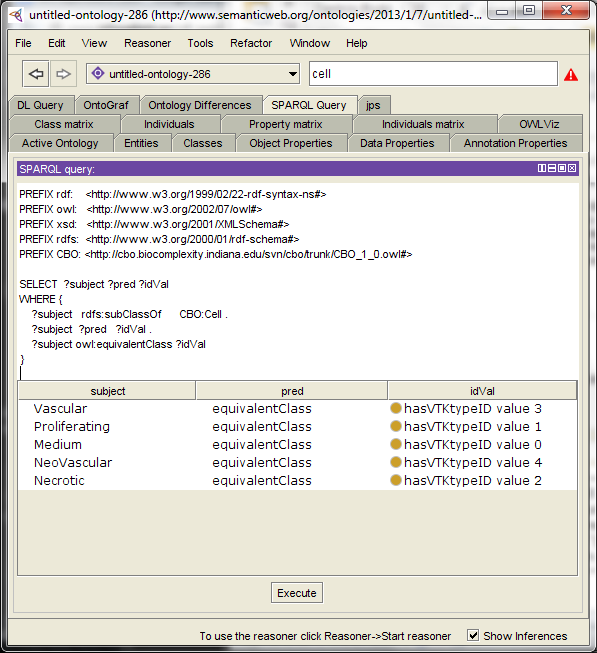


When doing SPARQL queries it is easy to get the PREFIX definitions wrong. In particular, if Protégé is importing an ontology the prefix of that ontology is sometimes not quite correct. In the case of our metamodel files the CBO classes are obtained via an import. To query using a **CBO** class name the PREFIX definition must be correct. You can get the correct PREFIX definition from either the "usage" or "OWLdoc" frame of the "Class" tab. Below is a screen shot showing the correct PREFIX (you need to drop the actual class name part, "Cell" in this case).


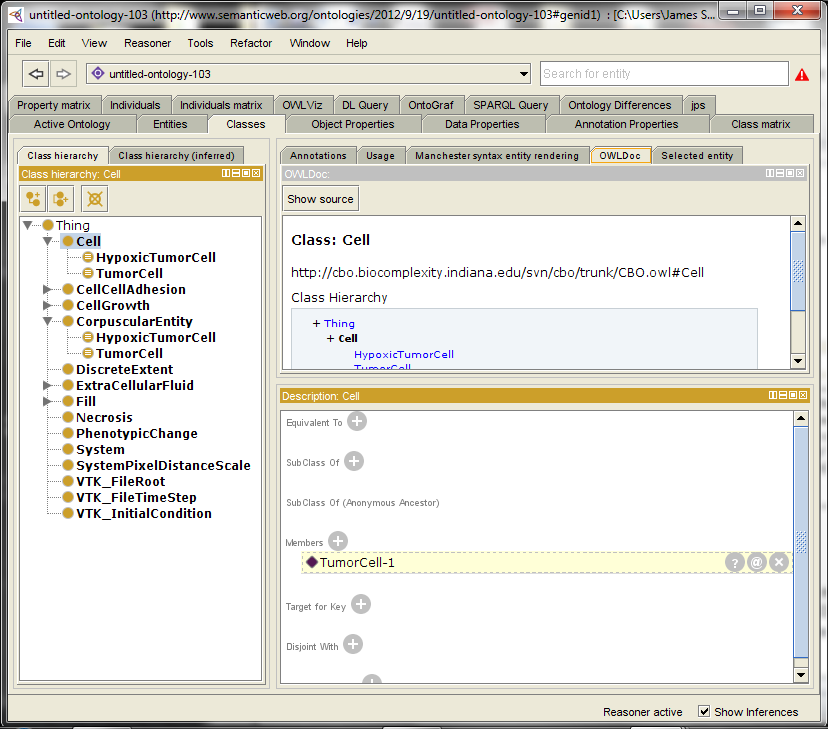


From the above the proper PREFIX command for CBO would be:
PREFIX CBO: <http://cbo.biocomplexity.indiana.edu/svn/cbo/trunk/CBO.owl#>

# Sample Python programs for extracting information

Here we present an example of extracting information from the combination of a model-specific CBO-OWL metamodel (ontology) and a set of VTK files produced when the Compucell3D (CC3D) model (that the CBO-OWL metamodel describes) is executed. The files needed can be downloaded from http://cbo.biocomplexity.indiana.edu/cbo/Step-by-Step/.

The CBO-OWL metamodel file is AngioTumor.owl. The set of VTK files is in the folder \VascularTumor\Compucell3D Files\VascularTumor_cc3d_02_18_2013_15_16_41. The VTK files are named using the step number. For example step_0000.vtk contains the cell and field data for the start of the simulation. The file Step_5400.vtk contains the cell and field data at the 5400th Monty Carlo step.

We have written a demonstration program in Python that opens the CBO-OWL file (AngioTumor.owl) and extracts the cell type IDs and cell names as well as the name of the model's diffusible fields. The program then iterates over the set of VTK files and extracts some statistics on the number and types of cells present at a particular time as well as the total amount of the three molecules that are diffusible fields in the CC3D model.

The Python script is available at http://cbo.biocomplexity.indiana.edu/cbo/Step-by-Step/­Demo_data_extract_CBO_VTK.py

Execution of the above Python script produces the output:

Owl file name= C:/Users/James Sluka/Desktop/Biocomplexity/CBO paper/Final Versions/Supplemental Material/Step_by_step/VascularTumor/AngioTumor.owl

In get_VTKdata_from_CBO_descr(OWLfile): Found 609 triples in the OWL file.

Name Spaces found in the OWL file:

http://www.semanticweb.org/ontologies/2013/1/7/untitled-ontology-286#

CBO_1_0 http://cbo.biocomplexity.indiana.edu/svn/cbo/trunk/CBO_1_0.owl#

CBO_1_02 http://cbo.biocomplexity.indiana.edu/svn/cbo/trunk/CBO_1_0#

dc http://purl.org/dc/elements/1.1/

oboInOwl http://www.geneontology.org/formats/oboInOwl#

owl http://www.w3.org/2002/07/owl#

rdf http://www.w3.org/1999/02/22-rdf-syntax-ns#

rdfs http://www.w3.org/2000/01/rdf-schema#

xml http://www.w3.org/XML/1998/namespace

xsd http://www.w3.org/2001/XMLSchema#

Searching for system parameters:

Looking up unit names:

http://purl.obolibrary.org/obo/UO_0000017 http://www.w3.org/2000/01/rdf-schema#label = micrometer

http://purl.obolibrary.org/obo/UO_0000031 http://www.w3.org/2000/01/rdf-schema#label = minute

In Main():

Cell Type ID numbers

ID Name

0 Medium

1 Proliferating

2 Necrotic

3 Vascular

4 NeoVascular

Field Names:

Glucose

VEGF1

VEGF2

System Parameters:

SystemPixelDistanceScale = 4.0

SystemPixelDistanceScale unit = micrometer

VTK_FileTimeStep = 1.0

VTK_FileTimeStep unit = minute

Finished parsing OWL file: ... p/VascularTumor/AngioTumor.owl

Processing VTK files, found 10 VTK files

Processing VTK File = Step_0000.vtk MCS= 0 Processing fields...

Processing VTK File = Step_0600.vtk MCS= 600 Processing fields...

Processing VTK File = Step_1200.vtk MCS= 1200 Processing fields...

Processing VTK File = Step_1800.vtk MCS= 1800 Processing fields...

Processing VTK File = Step_2400.vtk MCS= 2400 Processing fields...

Processing VTK File = Step_3000.vtk MCS= 3000 Processing fields...

Processing VTK File = Step_3600.vtk MCS= 3600 Processing fields...

Processing VTK File = Step_4200.vtk MCS= 4200 Processing fields...

Processing VTK File = Step_4800.vtk MCS= 4800 Processing fields...

Processing VTK File = Step_5400.vtk MCS= 5400 Processing fields...

VTK 'fields' in the VTK files:

CellId

CellType

CellVolume

ClusterId

Glucose

VEGF1

VEGF2

Finished parsing VTK files.

Total Cell Volumes by cell type (micrometer^3):

MCS minute(s) Medium Proliferating Necrotic Vascular NeoVascular

0 0.0 12645120 41600 0 96576 16704

600 600.0 12537024 154048 0 91776 17152

1200 1200.0 12494912 187648 768 92352 24320

1800 1800.0 12448512 229888 0 92800 28800

2400 2400.0 12374784 297792 0 93120 34304

3000 3000.0 12288256 372160 0 92864 46720

3600 3600.0 12139712 482496 0 93248 84544

4200 4200.0 11922816 639680 0 92352 145152

4800 4800.0 11688448 827456 0 91840 192256

5400 5400.0 11325248 1124736 0 91904 258112

Average field pixel values:

MCS minute(s) Glucose VEGF1 VEGF2

0 0.0 4.79804 0.00008 0.00003

600 600.0 8.81348 0.00717 0.00651

1200 1200.0 9.51673 0.00753 0.00710

1800 1800.0 9.26159 0.00803 0.00795

2400 2400.0 8.48257 0.00838 0.00991

3000 3000.0 7.49264 0.00908 0.00988

3600 3600.0 6.84573 0.01133 0.00821

4200 4200.0 7.33945 0.01587 0.00791

4800 4800.0 8.20651 0.01937 0.01043

5400 5400.0 7.95438 0.02361 0.01366

Cell Counts:

MCS minute(s) Proliferating Necrotic Vascular NeoVascular

0 0.0 64 0 24 4

600 600.0 64 0 24 4

1200 1200.0 63 1 24 5

1800 1800.0 87 0 24 5

2400 2400.0 102 0 24 6

3000 3000.0 111 0 24 10

3600 3600.0 184 0 24 17

4200 4200.0 209 0 24 27

4800 4800.0 308 0 24 34

5400 5400.0 386 0 24 44

# Configuring Protégé 4

Protégé 4 implements a bewildering number of tabs, views and plugins. A few hints on configuring Protégé is given below.

## Annotation Template

The Annotation Template facilitates the consistent annotation of OWL objects. With this template you can define the particular set of annotations that every object in the ontology should have.

1. Download the Annotation Template Jar file from <http://protegewiki.stanford.edu/wiki/Annotation_Template_View> and place it in the Protégé Plugins folder (typically something like C:\Program Files\Protege_4.3\plugins)
2. Start protégé and enable the view by selecting **Window | Views | Misc views | Annotation template** from the menu
3. Configure the view with **File | Preferences | Annotation template**
4. Save the tab

## Render with Label vs. Name

The CBO was developed with human readable names for the classes. Many ontologies use numeric names for classes along with a human readable label. For example, the Gene Ontology class GO:0008219 (the class name) has the label "cell death". Protégé 4 can be configured to display classes using either the class name or the class label. Go to File|Preferences then choose the "Renderer" tab. To e render entities using the rdfs:name field select "Render by annotation property" then click the "Configure" button and then click the add item icon and select the desired annotation types such as "label" (http://www.w3.org/rdf-schema#label).

## Reasoners

We have not made extensive use of OWL reasoners beyond simple consistence checking of both the CBO OWL and the metamodel files. Currently we use the Pellet reasoner for this purpose.

1. http://protegewiki.stanford.edu [↑](#footnote-ref-1)
2. http://www.compucell3d.org/ [↑](#footnote-ref-2)
3. Shirinifard, A. et al. (2009) 3D Multi-Cell Simulation of Tumor Growth and Angiogenesis. PLoS ONE, 4,e7190. [↑](#footnote-ref-3)
