# Supplementary material for: The cell behavior ontology: describing the intrinsic biological behaviors of real and model cells seen as active agents
Source: Bioinformatics. 2014 Apr 22;30(16):2367–74. doi: 10.1093/bioinformatics/btu210 (PMC4133580; doi:10.1093/bioinformatics/btu210)
Supplement: Supplementary Data [file supp_btu210_Supplemental_Material.zip › Supplemental Material/Supplement_1.docx]

**Supplement 1**

Supplementary material for "The Cell Behavior Ontology: Describing the intrinsic biological behaviors of real and model cells seen as active agents" by JP Sluka, A Shirinifard, M Swat, A Cosmanescu, R Heiland and JA Glazier

**Contents**

[Glossary of Terms with hyperlinks 2](#_Toc375562732)

[Cell Behavior Ontology (CBO) Object and Data Properties 3](#_Toc375562733)

[Object properties: 3](#_Toc375562734)

[Data properties: 4](#_Toc375562735)

[CBO annotation of the model components in Fig. 1 5](#_Toc375562736)

# Glossary of Terms with hyperlinks

| **BFO** | Basic Formal Ontology <http://www.ifomis.org/bfo> |
| --- | --- |
| **BioModels** | SBML model repository <http://www.ebi.ac.uk/biomodels-main/> |
| **BioPortal** | OWL ontology repository <http://bioportal.bioontology.org/> |
| **CBO** | Cell Behavior Ontology <http://cbo.biocomplexity.indiana.edu/cbo/> or http://bioportal.bioontology.org/ontologies/CBO |
| **CHASTE** | Cancer Heart and Soft Tissue Environment  <http://www.cs.ox.ac.uk/chaste/cell_based_index.html> |
| **ChEBI** | Chemical Entities of Biological Interest <http://www.ebi.ac.uk/chebi/> |
| **Compucell3D** | Open source modeling environment for cellular behavior  <http://www.compucell3d.org/> |
| **FieldML** | Fieled Markup Language <http://models.fieldml.org/fieldml> |
| **FMA** | Foundational Model of Anatomy <http://sig.biostr.washington.edu/projects/fm/> |
| **GO** | Gene Ontology <http://www.geneontology.org/> |
| **MIRIAM** | Minimum Information Required in the Annotation  of Models <http://www.ebi.ac.uk/miriam/main/> |
| **OBO** | Open Biomedical Ontologies <http://www.obofoundry.org/> |
| **OPB** | Ontology of Physics for Biology  <http://sbp.bhi.washington.edu/projects/the-ontology-of-physics-for-biology-opb> |
| **OpenAlea** | Software Environment for Plant Modeling  <http://openalea.gforge.inria.fr/dokuwiki/doku.php> |
| **OWL** | Web Ontology Language <http://www.w3.org/2001/sw/wiki/OWL> |
| **PATO** | Phenotypic Quality Ontology <http://obofoundry.org/wiki/index.php/PATO:Main_Page> |
| **Protégé 4** | Protégé-OWL editor <http://protege.stanford.edu> |
| **RO** | Relation Ontology <http://purl.obolibrary.org/obo/ro.owl> |
| **SBML** | Systems Biology Markup Language <http://sbml.org/Main_Page> |
| **SBO** | Systems Biology Ontology <http://www.ebi.ac.uk/sbo/main/> |
| **UO** | Unit Ontology <http://code.google.com/p/unit-ontology/> |
| **VTK** | Visualization Toolkit <http://www.vtk.org/>  <http://www.vtk.org/VTK/img/file-formats.pdf> |
| **XML** | eXtensible Markup Language |

# Cell Behavior Ontology (CBO) Object and Data Properties

## Object properties:

CBO OWL:Object Properties include the Relationship Ontology^[[1]](#footnote-1)^ plus the CBO defined relationship "has_Quality". Table 1 lists the OBO_REL (OBO version of the Relation Ontology) IDs are given in the "alt_id" column, the "Trans" and "Refl" columns indicate relationships that are transitive and reflexive, respectively.

Table 1: Object Properties in CBO.


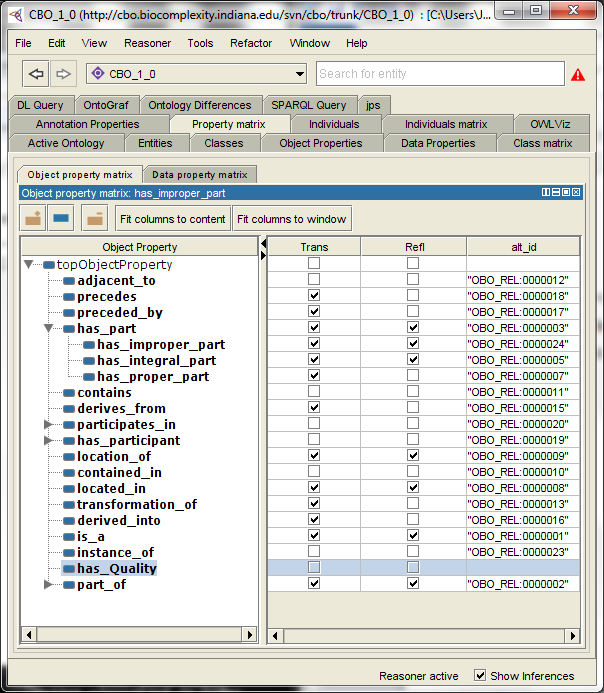


## Data properties:

CBO includes a number of OWL Data Properties. Table 2 lists the Data Properties, all of which are Functional.

Table 2: Data Properties in CBO


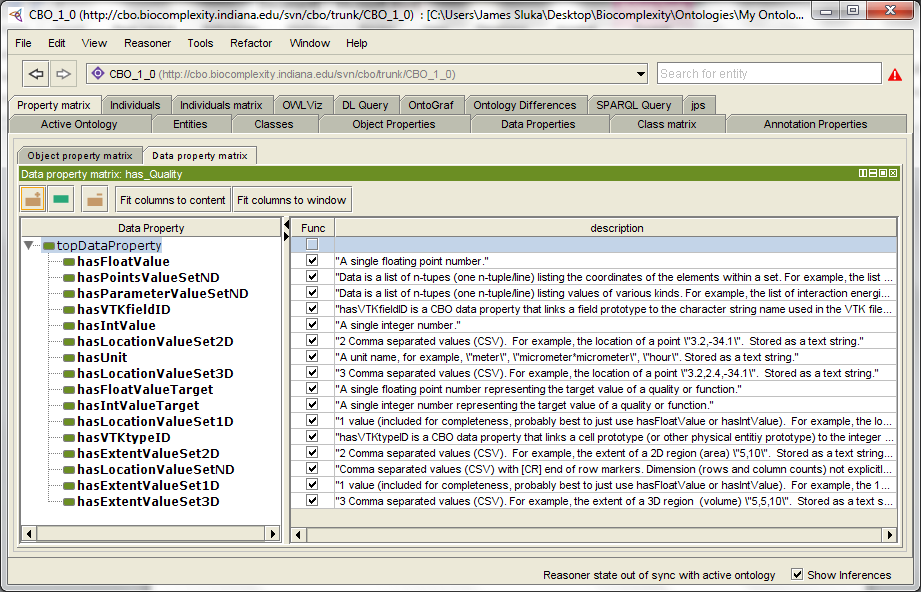


# CBO annotation of the model components in Fig. 1

Table 3 gives representative CBO annotations of the model components in Fig. 1 of the paper.

Table 3 Assignment of CBO terms to components of the model in Fig. 1 (after Shirinifard, 2009). Bold face denotes OWL Classes and italics for OWL *properties* (relations). "*isA*" is equivalent to *OWL:subClassOf* and *participates_in* is the inverse of *has_participant*.

| **Cells** | **Behaviors** | **CBO Term** |
| --- | --- | --- |
| **Tumor cells** |  | *isA* **Cell**  *isA* **CorpuscularEntity** |
| Normal | -proliferate | *participates_in* **CellGrowth**  *participates_in* **SymmetricCellDivision­Random­CleavagePlane** |
|  | -consume oxygen field | *participates_in* **MoleculeDeletion** |
|  | -change to hypoxic | *participates_in* **PhenotypicChange** |
|  | -change to necrotic | *participates_in* **PhenotypicChange** |
| Hypoxic | -proliferate | *participates_in* **CellGrowth**  *participates_in* **SymmetricCellDivision­Random­CleavagePlane** |
|  | -consume oxygen field | *participates_in* **MoleculeDeletion** |
|  | -change to normal | *participates_in* **PhenotypicChange** |
|  | -change to necrotic | *participates_in* **PhenotypicChange** |
|  | -secrete long-diffusing proangiogenic field V | *participates_in* **MoleculeDeletion** |
| Necrotic | -shrink | *participates_in* **CellVolumeChange** |
|  | -disappear | *participates_in* **Necrosis** |
| **Endothelial cells** |  | *isA* **Cell**  *isA* **CorpuscularEntity** |
| Vascular | -consume oxygen field | *participates_in* **MoleculeDeletion** |
|  | -supply oxygen field at partial pressure P | *participates_in* **MoleculeCreation** |
|  | -secrete short-diffusing chemoattractant field C | *participates_in* **MoleculeCreation** |
|  | -chemotax up gradients of field C | *participates_in* **Chemotaxis** |
|  | -elastically connect to neighboring vascular and inactive neovascular cells | *participates_in* **CellCellAdhesion**  has_part **CellPart:­CellMembranePart:­TightJunction** |
| **Fields** |  |  |
| Oxygen |  | *isA* **Molecule**, *isA* **DiffuseEntity**, *isA* **MolecularField**  *participates_in* **Diffusion**, *participates_in* **Creation**  *participates_in* **Deletion** |

1. Smith B, Ceusters W, Klagges B, Kohler J, Kumar A, Lomax J, Mungall CJ, Neuhaus F, Rector A, Rosse C "Relations in Biomedical Ontologies" Genome Biology, 2005, 6:R46. <http://purl.obolibrary.org/obo/ro.owl> [↑](#footnote-ref-1)
