# Supplementary figures and images for: The cell behavior ontology: describing the intrinsic biological behaviors of real and model cells seen as active agents
Source: Bioinformatics. 2014 Apr 22;30(16):2367–74. doi: 10.1093/bioinformatics/btu210 (PMC4133580; doi:10.1093/bioinformatics/btu210)

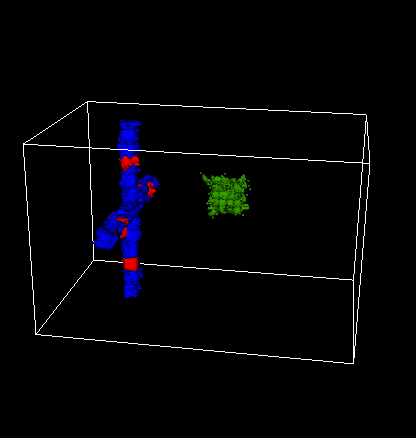

Supplement: Supplementary Data [file supp_btu210_Supplemental_Material.zip › Supplemental Material/CBO_Step_by_step/VascularTumor/Compucell3D Files/VascularTumor_cc3d_02_18_2013_15_16_41/VascularTumor_cc3d_0000.png]

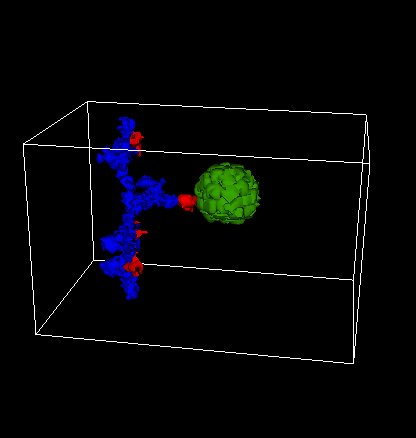

Supplement: Supplementary Data [file supp_btu210_Supplemental_Material.zip › Supplemental Material/CBO_Step_by_step/VascularTumor/Compucell3D Files/VascularTumor_cc3d_02_18_2013_15_16_41/VascularTumor_cc3d_0600.png]

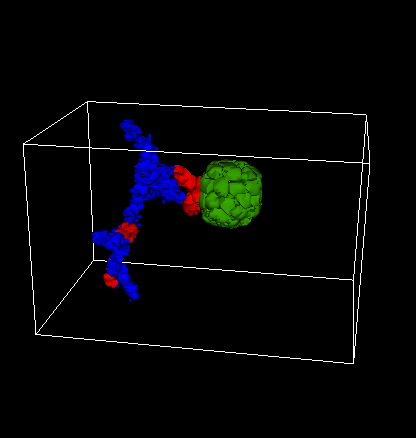

Supplement: Supplementary Data [file supp_btu210_Supplemental_Material.zip › Supplemental Material/CBO_Step_by_step/VascularTumor/Compucell3D Files/VascularTumor_cc3d_02_18_2013_15_16_41/VascularTumor_cc3d_1200.png]

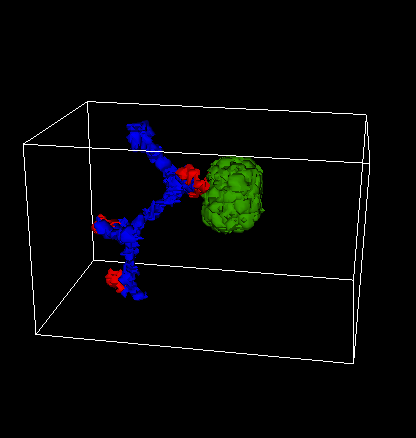

Supplement: Supplementary Data [file supp_btu210_Supplemental_Material.zip › Supplemental Material/CBO_Step_by_step/VascularTumor/Compucell3D Files/VascularTumor_cc3d_02_18_2013_15_16_41/VascularTumor_cc3d_1800.png]

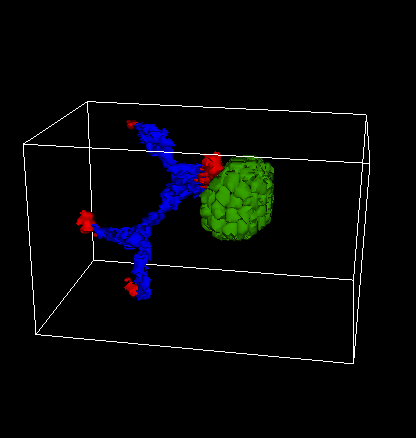

Supplement: Supplementary Data [file supp_btu210_Supplemental_Material.zip › Supplemental Material/CBO_Step_by_step/VascularTumor/Compucell3D Files/VascularTumor_cc3d_02_18_2013_15_16_41/VascularTumor_cc3d_2400.png]

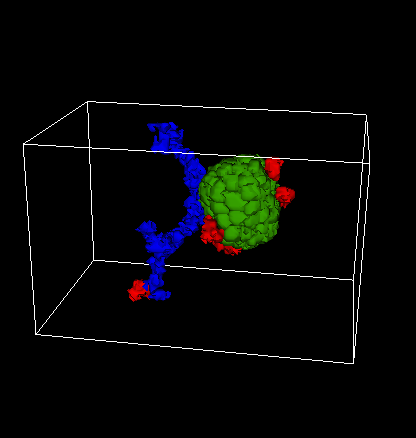

Supplement: Supplementary Data [file supp_btu210_Supplemental_Material.zip › Supplemental Material/CBO_Step_by_step/VascularTumor/Compucell3D Files/VascularTumor_cc3d_02_18_2013_15_16_41/VascularTumor_cc3d_3000.png]

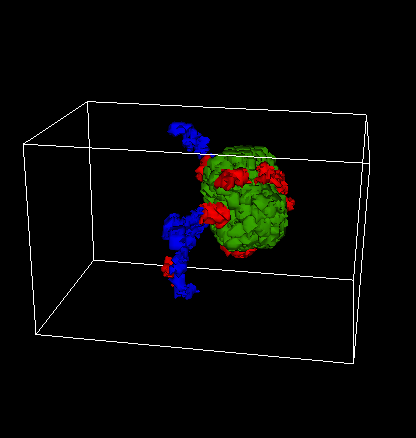

Supplement: Supplementary Data [file supp_btu210_Supplemental_Material.zip › Supplemental Material/CBO_Step_by_step/VascularTumor/Compucell3D Files/VascularTumor_cc3d_02_18_2013_15_16_41/VascularTumor_cc3d_3600.png]

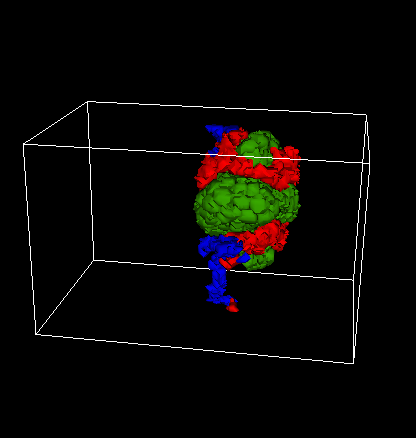

Supplement: Supplementary Data [file supp_btu210_Supplemental_Material.zip › Supplemental Material/CBO_Step_by_step/VascularTumor/Compucell3D Files/VascularTumor_cc3d_02_18_2013_15_16_41/VascularTumor_cc3d_4200.png]

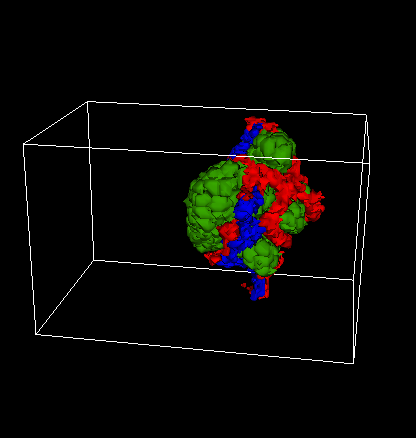

Supplement: Supplementary Data [file supp_btu210_Supplemental_Material.zip › Supplemental Material/CBO_Step_by_step/VascularTumor/Compucell3D Files/VascularTumor_cc3d_02_18_2013_15_16_41/VascularTumor_cc3d_4800.png]

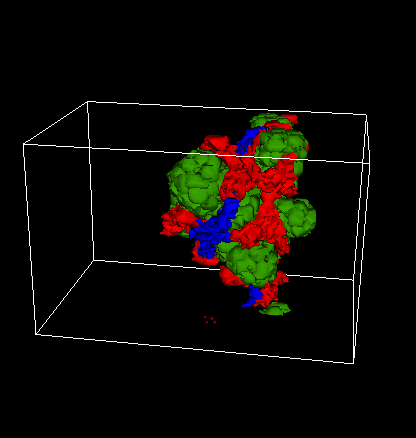

Supplement: Supplementary Data [file supp_btu210_Supplemental_Material.zip › Supplemental Material/CBO_Step_by_step/VascularTumor/Compucell3D Files/VascularTumor_cc3d_02_18_2013_15_16_41/VascularTumor_cc3d_5400.png]

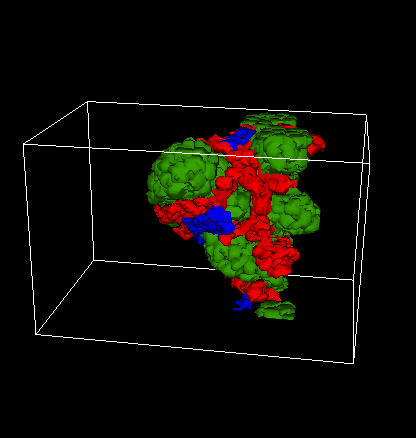

Supplement: Supplementary Data [file supp_btu210_Supplemental_Material.zip › Supplemental Material/CBO_Step_by_step/VascularTumor/Compucell3D Files/VascularTumor_cc3d_02_18_2013_15_16_41/VascularTumor_cc3d_6000.png]
